# The Epipolythiodiketopiperazine Gene Cluster in *Claviceps purpurea*: Dysfunctional Cytochrome P450 Enzyme Prevents Formation of the Previously Unknown Clapurines

**DOI:** 10.1371/journal.pone.0158945

**Published:** 2016-07-08

**Authors:** Julian Dopstadt, Lisa Neubauer, Paul Tudzynski, Hans-Ulrich Humpf

**Affiliations:** 1 Institute of Food Chemistry, Westfälische Wilhelms-Universität Münster, Corrensstraße 45, 48149 Münster, Germany; 2 Institute of Plant Biology and Biotechnology, Westfälische Wilhelms-Universität Münster, Schlossplatz 8, 48143 Münster, Germany; The University of Wisconsin—Madison, UNITED STATES

## Abstract

*Claviceps purpurea* is an important food contaminant and well known for the production of the toxic ergot alkaloids. Apart from that, little is known about its secondary metabolism and not all toxic substances going along with the food contamination with *Claviceps* are known yet. We explored the metabolite profile of a gene cluster in *C*. *purpurea* with a high homology to gene clusters, which are responsible for the formation of epipolythiodiketopiperazine (ETP) toxins in other fungi. By overexpressing the transcription factor, we were able to activate the cluster in the standard *C*. *purpurea* strain 20.1. Although all necessary genes for the formation of the characteristic disulfide bridge were expressed in the overexpression mutants, the fungus did not produce any ETPs. Isolation of pathway intermediates showed that the common biosynthetic pathway stops after the first steps. Our results demonstrate that hydroxylation of the diketopiperazine backbone is the critical step during the ETP biosynthesis. Due to a dysfunctional enzyme, the fungus is not able to produce toxic ETPs. Instead, the pathway end-products are new unusual metabolites with a unique nitrogen-sulfur bond. By heterologous expression of the *Leptosphaeria maculans* cytochrome P450 encoding gene *sirC*, we were able to identify the end-products of the ETP cluster in *C*. *purpurea*. The thioclapurines are so far unknown ETPs, which might contribute to the toxicity of other *C*. *purpurea* strains with a potentially intact ETP cluster.

## Introduction

The biotrophic plant pathogen *Claviceps purpurea* infects a broad range of grasses including economically important cereal crop plants [1]. In the sclerotia, which are the overwintering structure of the fungus, *C*. *purpurea* produces the toxic ergot alkaloids. In the Middle Ages, the consumption of rye products contaminated with *C*. *purpurea* sclerotia led to the so-called St. Anthony’s Fire epidemics and also in the 20th century this risk was still present [2]. Biochemistry and genetics of the ergot alkaloids biosynthesis have been well studied in *C*. *purpurea *[3], but apart from that, little is known about other secondary metabolites contributing to the toxicity of the ergot sclerotia.

In filamentous fungi, the biosynthetic genes for secondary metabolites are typically clustered together [4] and these clusters usually consist of at least one backbone gene such as polyketide synthases (PKSs) or nonribosomal peptide synthetases (NRPSs). The availability of the *C*. *purpurea* genome sequence allowed the identification of 9 PKS- and 18 NRPS-encoding genes through a bioinformatical screening approach, demonstrating the great potential of the reference strain *C*. *purpurea* to produce previously unknown secondary metabolites [5]. As putative secondary metabolite gene clusters are often silent under standard laboratory conditions, an activation of cryptic clusters by genetic manipulation is a common tool to identify new metabolites [6,7].

**Fig 1:**
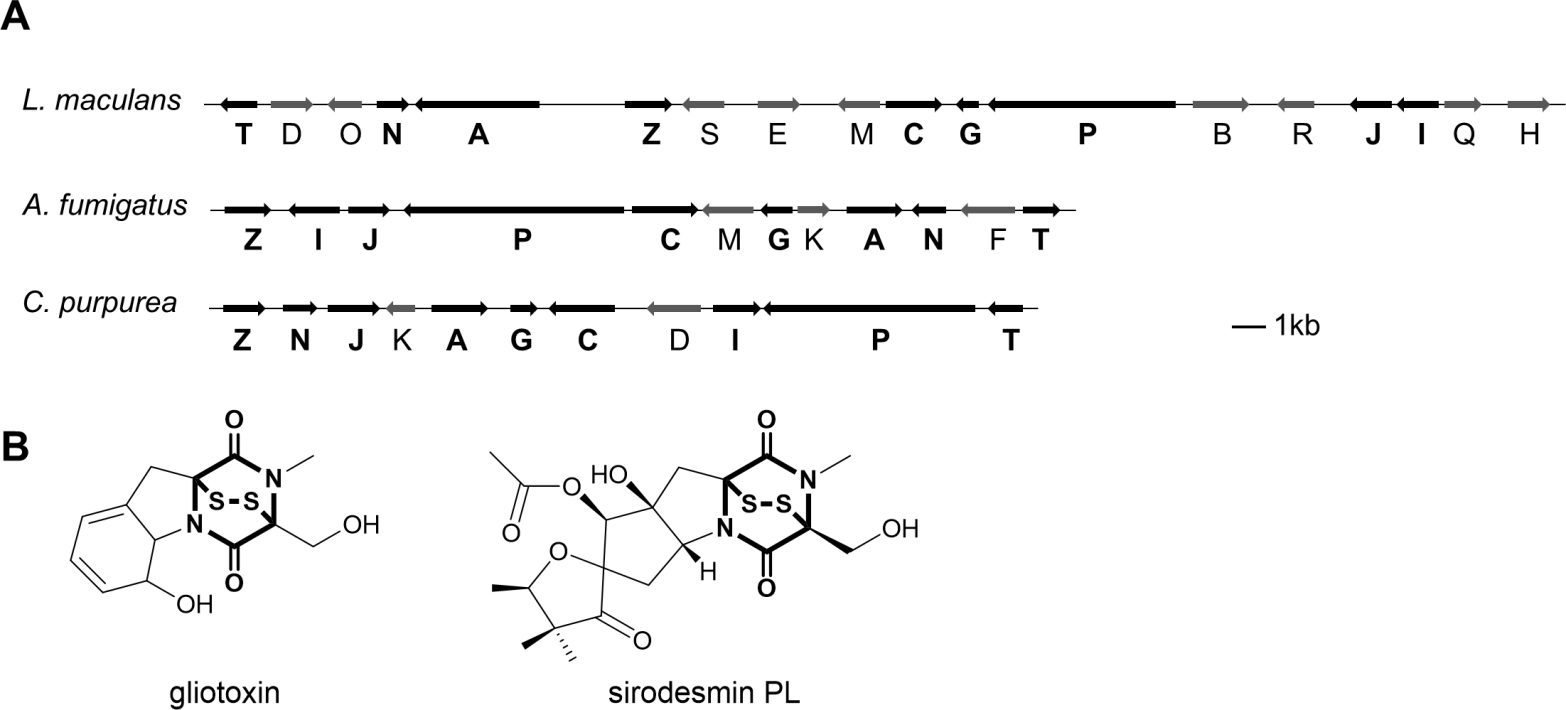
Organization of the different ETP biosynthesis gene clusters and structure of gliotoxin and sirodesmin. (A) Shown is the ETP gene cluster in *C*. *purpurea* in comparison to the gliotoxin and sirodesmin producing gene clusters from *A*. *fumigatus* and *L*. *maculans*. Orientation of the arrows indicates the direction of transcription. Genes in black are common ETP moiety genes present in all three clusters. For gene designations see Table 1. (B) Structure of gliotoxin and sirodesmin PL with the bolded characteristic diketopiperazine moiety with an internal disulfide bridge.

This paper reports the identification of a gene cluster in *C*. *purpurea* with *a* high similarity to gene clusters responsible for the formation of epipolythiodiketopiperazine (ETP) toxins in other fungi. This class of toxins is characterized by a diketopiperazine backbone (see bold structure element in Fig 1B) derived from two amino acids with an internal disulfide bridge. The disulfide bridge is responsible for the toxicity of the ETPs by inactivating proteins through thiol conjugation and the generation of reactive oxygen species via redox cycling [8–10]. ETPs are toxic to a broad range of organisms, including viruses, bacteria or fungi [9,11] and some have also been associated with mammalian diseases [12–14]. On the other hand, the cytotoxicity of the ETPs has made them attractive as potential drug candidates [15–17].

There is a great structural diversity of ETPs and so far over 100 different ETPs have been identified [18]. The diversity is due to variations in the set of amino acids which build the core ETP moiety. However, all known ETPs are derived from at least one aromatic amino acid [19]. ETPs can also differ in the amount of sulfur atoms. Most common are epidithiodiketopiperazines but epitri- and epitetrasulfide derivatives are also known [20,21]. One example for an ETP toxin is sirodesmin PL (Fig 1B). The phytotoxin contributes to the virulence of *Leptosphaeria maculans* causing yellow lesions on plant leaves [22]. Another well studied ETP is gliotoxin (Fig 1B), which plays a significant role in enabling the virulence of the human pathogen *Aspergillus fumigatus* causing invasive aspergillosis [23]. Gliotoxin was first discovered in the plant-beneficial fungus *Trichoderma virens* [24]. In 2012 the corresponding gene cluster could be identified [25] and a knock-out of the NRPS demonstrated that gliotoxin is involved in mycoparasitism of the fungus [26].

Putative ETP gene clusters are present in at least 14 ascomycete taxa including pathogens of mammals and plants [27]. The best characterized ETP clusters are the sirodesmin cluster in *L*. *maculans* [28] and the gliotoxin cluster in *A*. *fumigatus* [19]. In both clusters, genes encoding enzymes responsible for the formation of the common ETP moiety are present, like a NRPS, a cytochrome P450 monooxygenase and a methyltransferase [29]. A glutathione S-transferase is responsible for the sulfurization of the molecule by adding glutathione [30]. A further oxidoreductase mediates the formation of the disulfide bridge [31]. In *A*. *fumigatus* it was also shown that this oxidoreductase confers self-resistance of the fungus to gliotoxin [31,32]. The gliotoxin cluster is under the control of the Zn(II)_2_Cys_6_ transcription factor GliZ [33]. GliZ is also necessary for the production of several gliotoxin-related metabolites which appear to be shunt products of the gliotoxin pathway [34].

In this paper, we describe the identification of an ETP gene cluster in *C*. *purpurea* and the gene cluster activation through overexpression of the cluster specific transcription factor. We also report the identification of novel unusual metabolites as end-products of the ETP gene cluster in *C*. *purpurea* and show that due to a dysfunctional cluster gene, this *C*. *purpurea* strain is not able to produce known toxic forms of ETPs. By heterologous expression of a *L*. *maculans* sirodesmin cluster gene in *C*. *purpurea* and detailed nuclear magnetic resonance (NMR) as well as high resolution mass spectrometric (HRMS) studies, we identified so far unknown ETPs as end-products of the *C*. *purpurea* ETP cluster.

## Results

### Characteristics of the *C*. *purpurea* ETP cluster

Bioinformatic analysis revealed the presence of a *C*. *purpurea* NRPS-encoding gene (*CPUR_02680*) with significant sequence similarity to the ETP-toxin producing NRPSs in *A*. *fumigatus* and *L*. *maculans* [5]. By domain analysis using the NCBI Conserved Domain Database two AMPylation domains could be detected in the amino acid sequence of CPUR_02680 (TcpP). Analysis of the genes upstream and downstream of *tcpP* using the Basic Local Alignment Search Tool (BLAST) uncovered a cluster of 11 genes with high homology to the gliotoxin cluster genes in *A*. *fumigatus* and to the sirodesmin cluster genes in *L*. *maculans* (Fig 1, Table 1). Genes highly conserved between the three clusters encode a zinc finger transcription factor, a methyltransferase, a dipeptidase, a transporter, a glutathione S-transferase, a cytochrome P450 monooxygenase, an aminotransferase, a NRPS and an oxidoreductase. A *CPUR_02674* (*tcpK*) homolog is only present in the gliotoxin cluster and a *CPUR_02678* (*tcpD*) homolog only in the sirodesmin cluster, encoding a glutamyltransferase and a prenyl transferase, respectively. There are differences in the arrangement of the genes within the three ETP clusters and for some genes, present in the gliotoxin or the sirodesmin cluster, no homologs could be identified in the *C*. *purpurea* ETP cluster (Fig 1). These enzymes are probably involved in modifications of the side chains of the core ETP moiety, like the acetyl transferase *sirH* or the cytochrome P450 monooxygenases *gliF*, *sirB* and *sirE*.

**Table 1:** Predicted functions and homologs of the ETP gene cluster in *C*. *purpurea*.

Name	Gene Code	Homolog^a^	E-Value	Predicted Function
*tcpZ*	CPUR_02671	*gliZ*,*sirZ*	3.0e-142.7e-13	zinc finger transcription factor
*tcpN*	CPUR_02672	*gliN*,*sirN*	4.6e-372.9e-06	methyltransferase
*tcpJ*	CPUR_02673	*gliJ*,*sirJ*	1.0e-841.5e-104	dipeptidase
*tcpK*	CPUR_02674	*gliK*	4.3e-30	glutamyltransferase
*tcpA*	CPUR_02675	*gliA*	9.1e-106	transporter
*tcpG*	CPUR_02676	*gliG*,*sirG*	2.7e-392.0e-47	glutathione S-transferase
*tcpC*	CPUR_02677	*gliC*,*sirC*	1.2e-795.6e-92	cytochrome P450 monooxygenase
*tcpD*	CPUR_02678	*sirD*	3.6e-70	prenyl transferase
*tcpG*	CPUR_02679	*gliI*,*sirI*	1.0e-566.6e-77	aminotransferase
*tcpP*	CPUR_02680	*gliP*,*sirP*	3.6e-1390	NRPS
*tcpT*	CPUR_02681	*gliT*,*sirT*	1.8e-656.0e-57	oxidoreductase

^a^gli: gliotoxin cluster *A*. *fumigatus* [19], sir: sirodesmin cluster *L*. *maculans* [28].

### Expression studies

**Fig 2:**
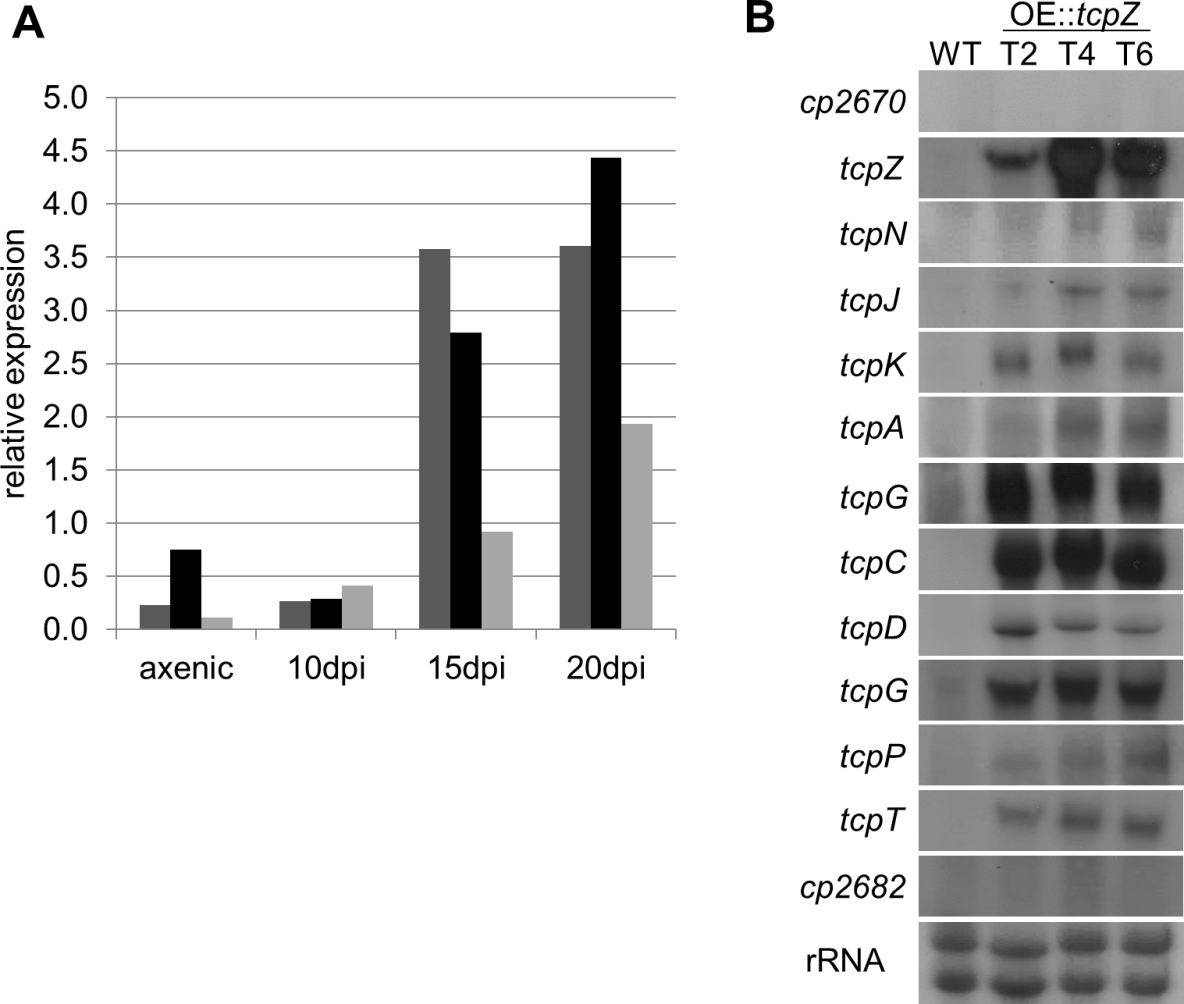
Gene expression and co-regulation of the ETP cluster genes. **(A)** *In planta* gene expression of *tcpG*. Shown are three independent experiments in the *C*. *purpurea* wild-type strain in planta 10, 15 and 20 days post-infection (dpi) and in axenic culture. Expression levels were normalized against the housekeeping genes encoding β-tubulin, γ-actin, and glyceraldehyde-3-phosphate dehydrogenase. **(B)** Co-regulation of the ETP cluster genes. The wild-type as well as three independent OE::*tcpZ* mutants were grown for seven days in liquid Mantle media and northern blot analysis was performed as described in methods.

To study if the identified gene cluster is transcribed *in planta*, expression studies of the putative ETP cluster gene *CPUR_02679* (*tcpG*) were carried out using quantitative reverse-transcription PCR (qRT-PCR) (primers can be found in S1 Table). Therefore, rye plants were infected with the *C*. *purpurea* strain 20.1, the sequenced standard wild-type strain 20.1 [5]. The *tcpG* gene expression 10, 15 and 20 days post-infection showed an increase towards late stages of infection (Fig 2A). This tendency was confirmed in three biological replicates although the data show high variability due to the heterogeneous biological material (the infection process cannot be completely synchronized).

To determine the expression in axenic culture, northern blot analyses were performed. Since no expression of any of the putative ETP cluster genes could be detected for the *C*. *purpurea* wild-type, we overexpressed *tcpZ* (*CPUR_02671*), encoding a putative cluster-specific transcription factor. For this purpose, an additional copy of the gene was introduced under the control of the strong, constitutive *A*. *nidulans* oliC promoter (S1 Fig). For three independent transformants the overexpression of *tcpZ* could be confirmed via northern blot. In all three transcription factor overexpression transformants (OE::*tcpZ*) the expression of the putative cluster genes *CPUR_02671* to *CPUR_02681* was significantly upregulated (Fig 2B). For the genes *CPUR_02670* (related to alpha-mannosidase) and *CPUR_02682* (thioredoxin-like protein), which flank the putative ETP cluster, no co-regulation with the putative cluster genes could be observed, confirming the predicted borders of the cluster. A significant influence of the *tcpZ* overexpression on the physiology of the fungus (growth, conidia formation) could not be observed (data not shown).

### Identification of pathway intermediates

We used a mass spectromic analysis of the metabolite profile of OE::*tcpZ* mutants to search for new ETP-like metabolites. Therefore, the metabolite profile of wild-type and OE::*tcpZ* strain, both cultivated in liquid Mantle media for seven days, were compared using reverse-phase–high-performance liquid chromatography–diode-array detection–high resolution mass spectrometry (RP-HPLC-DAD-HRMS). In order to evaluate the characteristic fragmentation behavior of ETPs with the used mass spectrometer, gliotoxin was used as reference compound. In this analytical procedure metabolites with a diketopiperazine (DKP) backbone and at least one sulfur bridge first show a neutral loss of both sulfur atoms and later the opening of the DKP ring, as already described in literature [35–37]. Based on this information every new peak in the mutant strain which was not present in the wild-type was analyzed by HRMS^n^. The HRMS data were further analyzed by Metworks (Thermo Scientific, Bremen, Germany), to search for compounds which are featured by the characteristic sulfur isotopic pattern and ratio. In addition, neutral loss experiments were implanted using a highly sensitive HPLC-MS/MS system to screen for minor compounds with disulfide bridges.

**Fig 3:**
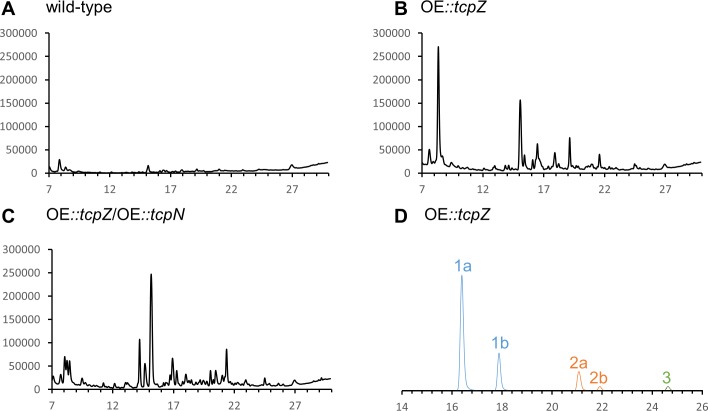
**Comparison of the HPLC-MS metabolite profile (total ion chromatograms) between the wild type of *Claviceps purpurea* strain 20.1 (A), OE::*tcpZ* (B) and OE::*tcpZ*/OE::*tcpN* (C). (D) Extracted ion signals of compounds 1a/b, 2a/b and 3 of OE::*tcpZ***.

**Fig 4:**
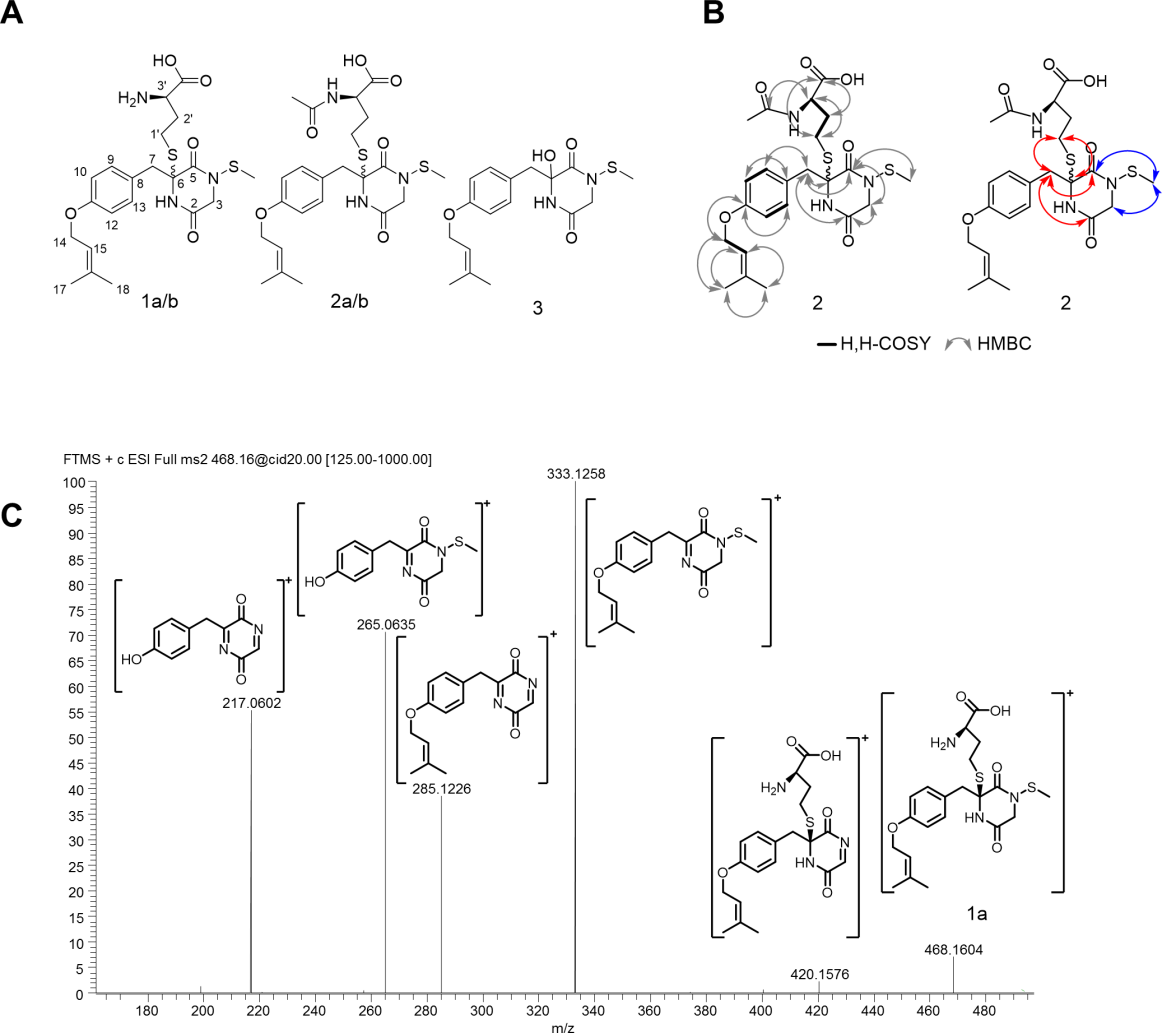
Newly identified secondary metabolites as products of an ETP gene cluster in *Claviceps purpurea*. **(A)** Compounds **1**–**3** were produced by OE::*tcpZ* strain *in planta* and in axenic culture. **(B)** Selected 2D- NMR data for **2.** Red arrows indicate the specific couplings of H-7a/b and the blue arrows those of S-CH_3_. **(C)** HRMS^n^ fragmentation of **1a** as [M+H]^+^ illustrating the rapid cleavage of the thiomethyl group and dimethylallyl group.

The HPLC-MS chromatograms of the wild-type and the OE::*tcpZ* strain showed several differences in the metabolite profile (Fig 3A and 3B). Surprisingly, the fragmentation pattern did not indicate the disulfide bridge characteristic of known ETPs. Nevertheless, based on HRMS and HRMS^n^ analysis, two TcpZ-dependent metabolites with the characteristic sulfur isotope pattern could be identified as **1a/b** (C_21_H_29_N_3_O_5_S_2_) and **2a/b** (C_23_H_31_N_3_O_6_S_2_) (Fig 4). Both compounds included two sulfur atoms and differed by the mass of a C_2_H_2_O-group indicating an acetyl group. The chromatographic separation of each metabolite in two peaks indicated the presence of diastereomers (Fig 3D). A first hint of the structure was obtained by the fragmentation pattern using HRMS^n^, as shown in Fig 4C. Most striking was the cleavage of a dimethylallyl group, whose incorporation in the biosynthetic pathway of sirodesmin in *L*. *maculans* was already demonstrated [38]. Furthermore, the cleavage of homocysteine, respectively the acetylated homocysteine and the loss of a thiomethyl group as for example from *m/z* 333 to *m/z* 285 were characteristic. Compared to the elucidated fragmentation of gliotoxin the information from the HRMS^n^ experiments implied that both compounds were build out of tyrosine and dimethylallyl pyrophosphate and secondly glycine as the corresponding amino acid. The resulting DKP was then *N*-thiomethylated and finally homocysteine (or its acetylated form) was added during biosynthesis. Apart from **1** and **2** we noted the presence of a similar metabolite **3** (C_17_H_22_N_2_O_4_S) (Figs 3D and 4A). HRMS^n^ experiments clarified that instead of homocysteine a hydroxyl group was added at position C-6.

To provide security for the predicted structures, the compounds were isolated by solid phase extraction followed by preparative HPLC. The structures of **1a** (0.3 mg), **1b** (0.4 mg), **2a** (1.9 mg), **2b** (1.8 mg) and **3** (1.0 mg) were confirmed by detailed 1D- and 2D-nuclear magnetic resonance (NMR) experiments (see Fig 4B for selected 2D-NMR data of **2**, the complete NMR data of **1a/b**, **2a/b** and **3** can be found in the supporting information. The structures of **1a/b** were confirmed by comparing the ^1^H and ^13^C data to those of **2a/b**). ^1^H-NMR analysis of compound **2**, for example, clearly revealed the presence of a dimethylallyl moiety, which was characterized by the signals at δ = 5.42 (tdq, J = 7.3, 3.0, 1.4 Hz, H-15), 4,50 (m, H-14), 1.77 (d, J = 1.4 Hz, H-17) and 1.73 (d, J = 1.3 Hz, H-18) ppm and is described in previous studies [38,39]. Phase sensitive gradient Heteronuclear Single Quantum Correlelation (gHSQC) experiments established one-bond correlations between these protons and the corresponding carbon atoms at δ = 65,80 ppm (C-14), 120.90 ppm (C-15) and the methyl groups at δ = 25.74 ppm (C-17) and 18.19 ppm (C-18). Furthermore, the ^1^H NMR spectrum revealed four aromatic protons (H-10,12 δ = 6,9 ppm and H-9,13 δ = 7.16 ppm), roofed to each other and spit into doublets as an AA’BB’ system with the same chemical shift indicating a 1,4 di-substituted benzene ring. These protons could be assigned to the carbon signals of the aromatic signals at 116.12 ppm (C-10,12) and δ = 132.81 ppm (C-9,13). Heteronuclear Multiple Bond Correlated Spectroscopy (HMBC) couplings between the carbon atoms C-10 and C-12 and the proton H-14 demonstrated that the aromatic ring was substituted at position 4 by the dimethylallyl group. In the same way the carbon atoms C-9 and C-13 showed strong couplings to a methylene group; showing a noticeable splitting of the chemical shift (H-7a δ = 2.66 ppm and H-7b δ = 3.65 ppm). The DKP moiety was indicated by HMBC couplings between H-7a/b and the carbon atoms at δ = 164.14 and 165.05 ppm as well as couplings to a quaternary carbon at δ = 65.30 ppm. The expected carbonyl groups of the DKP core could be assigned to the carbons of C-2 (δ = 164.14 ppm) and C-5 (δ = 165.05 ppm). Further HMBC couplings between the protons of δ = 3.44/4.19 ppm and the carbon atoms C-2 and C-5 correspond to a coupling to H-3a/b and confirm the predicted DKP moiety. The ^13^C NMR signal at δ = 13.65 ppm could be matched to a thiomethyl group. Due to exclusive HMBC couplings (Fig 4B, blue arrows) to C-3 and C-5 the unusual thiomethyl group could be located at N-4. H,H-COSY correlations and HMBC couplings between the protons at δ = 2.88, 2.10 and 3.62 ppm and their corresponding carbon atoms at δ = 26.90, 32.09 and 55.32 ppm give connectivity information about the substituted homocysteine and the acetyl group. HMBC measurements established the linkage between the DKP core and the substituted homocysteine. The couplings between the protons of H-7a/b and the carbons C-2, C-5, C-1’ make the connection obvious (Fig 4B, red arrows). The NMR data of **3** only differed in the lack of signals corresponding to the homocysteine residue and a chemical shift of the carbon atom of C-6 from δ = 65.30 ppm to δ = 75.90 ppm.

As in the OE::*tcpZ* strain no metabolites were detectable with a hydroxylated α-carbon of glycine, we hypothesized that in *C*. *purpurea* instead of the carbon the nitrogen of the glycine part is oxidized. In *A*. *fumigatus* a cytochrome P450 monooxygenase is responsible for the bishydroxylation of the DKP during gliotoxin biosynthesis and thus enables the addition of glutathione at the α-carbon of each amino acid [30]. Previous studies of the gliotoxin pathway in *A*. *fumigatus* already indicated the possible feature of the cytochrome P450 enzyme to form *N*-oxidized shunt products [34,40]. Another example is the cytochrome P450 CypX in *Bacillus subtilis*, which is known to mediate the *N*-oxidation of diketopiperazines [41]. A subsequent addition of glutathione followed by the degradation to a thiol and finally a methylation could explain the unique *N*-thiomethyl structure in *C*. *purpurea*. In *A*. *fumigatus*, *gliN*, is responsible for the methylations during the ETP biosynthetic pathway [42] leading to the stabilization of the DKP [43]. To get further insights into the biosynthetic pathway in *C*. *purpurea*, we overexpressed the putative methyltransferase-encoding gene *tcpN* (*CPUR_02672*) in the OE::*tcpZ* background by introducing an additional copy of the gene under the control of the strong constitutive *A*. *nidulans* oliC promoter (S2 Fig) in order to obtain a *N*-methoxy diketopiperazine core.

**Fig 5:**
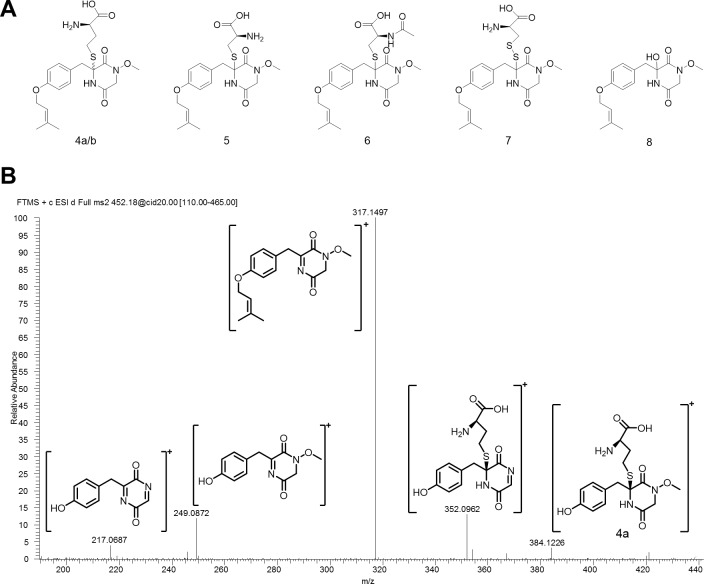
Identified structures of the OE::*tcpZ*/OE::*tcpN* strain. (**A**) **4-8** occur in axenic culture. (**B**) HRMS^n^ analysis of **4a** as [M+H]^+^ clarifies the characteristic fragmentation and underlines the differences concerning the N-4 substitution. This characteristic fragmentation allows structure elucidation of other unknown intermediates based on HRMS^n^.

Compositions of the culture filtrates of the wild-type, the OE::*tcpZ* strain and the OE::*tcpZ*/OE::*tcpN* strain were compared by HPLC-DAD-HRMS and HRMS^n^. On the one hand this analysis revealed that the two major compounds **1a/b** and **2a/b** as well as **3** were missing in the extract from the OE::*tcpZ*/OE::*tcpN* strain. On the other hand, several other metabolites (**4**-**6**; Figs 3C and 5A) with the same carbon skeleton and the molecular formulas **4a** (C_21_H_29_N_3_O_6_S, 1.1 mg), **4b** (C_21_H_29_N_3_O_6_S, only traces) **5** (C_20_H_27_N_3_O_6_S, 1.3 mg) and **6** (C_22_H_30_N_4_O_7_S, 1.5 mg), indicating the substitution of one sulfur atom by oxygen, could be detected. Fig 5B illustrates that the fragmentation pathway confirms the *N*-methoxylated compounds **4-6**, as we could clearly characterize the cleavage of a methoxy group at N-4, for example from m/z 249 to m/z 217. The *N*-methoxy group could be confirmed by comparing the ^13^C NMR chemical shift of **2** and **4**. Whereas the carbon of the methoxy group in **4a** showed a chemical shift of 64.80 ppm (S10B Fig), the thiomethyl group in **2a/b** differs with a typical shift of 13.65 ppm (S7B and S8B Figs) which is in excellent agreement with literature data [44] and previous NMR data concerning the nitrogen-methoxy-group [34]. The ^1^H NMR spectra further showed a shift of the corresponding protons from δ = 3.32 ppm (S7B and S8B Figs) (**2**) to δ = 2.20 ppm (S10B Fig) (**4**). Apart from the described chemical shifts the other ^1^H- and ^13^C NMR signals of **2a/b** and **4a** were comparable.

In addition to **4**–**6** the metabolites **7** (C_20_H_27_N_3_O_6_S_2_) and **8** (C_17_H_22_N_2_O_5_) shown in Fig 5A were also identified in the OE::*tcpZ*/OE::*tcpN* mutant by HRMS. Metabolite **8** is in accordance with the known biosynthetic pathway [40] as it represents the hydroxylated intermediate of the α-carbon of tyrosine. Due to the low concentration **8** could only be characterized by HRMS^n^. Interestingly, the OE::*tcpZ*/OE::*tcpN* mutants also produced metabolite **7,** containing again two sulfur atoms. The fragmentation revealed the characteristic pattern containing the methoxy group at N-4 as shown before. Conclusively a group at C-6 with a pseudo molecular formula of C_3_H_5_O_2_NS_2_ was cleaved during fragmentation resulting in the structure of **7**, corresponding to cysteine bound via a disulfide bridge. In order to confirm this predicted structure 1D- and 2D- NMR experiments were performed. The ^1^H NMR of **7** (0.6 mg) (S13A Fig) revealed that most signals were similar to that of **5** (S11A Fig). Unfortunately, the signals in the ^13^C NMR were limited due to the small amount of the isolated compound. Nevertheless, HMBC couplings between C-6 and H-7a/b demonstrated a downfield shift of the carbon, which confirmed the predicted structure.

### TcpC as the critical enzyme during ETP biosynthesis

In all analyzed mutants, we identified neither sulfur bridged ETP end-products nor intermediates, where the α-carbon of glycine is hydroxylated or glutathione is attached. Therefore, we concluded that bishydroxylation of the DKP is the critical step during ETP biosynthesis in *C*. *purpurea* and that TcpC oxidizes only the α-carbon of tyrosine, but not the second one of glycine. Thus, the second sulfur molecule could not be incorporated and the formation of a DKP with an internal disulfide bridge is not possible.

**Fig 6:**
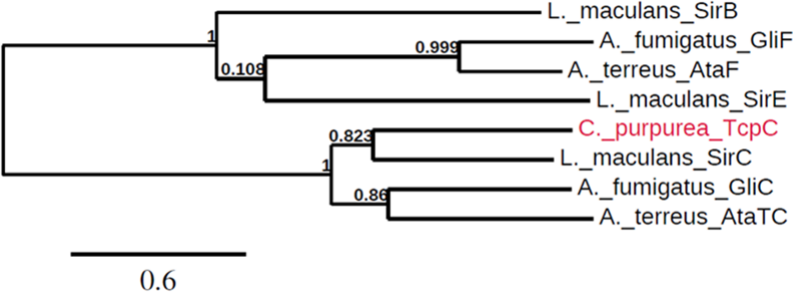
Phylogenetic analysis of TcpC and its homologs from other fungal ETP clusters. Amino acid sequences were obtained from GenBank database (GliC EDP49542.1, GliF AAW03300.1, SirE AAS92549.1, SirB XP_003842422.1, SirC AAS92547.1, ataF XM_001212649.1 ataTC XM_001212652.1). Sequences were aligned with MUSCLE (v3.8.31) and the phylogenetic tree was constructed with the maximum likelihood method using Phylogeny.fr [83]. Branch support values are indicated.

The *A*. *fumigatus* gliotoxin gene cluster contains two cytochrome P450 monooxygenase genes. GliC is reported to be responsible for the bishydroxylation of the DKP during gliotoxin biosynthesis, whereas the second cytochrome P450 enzyme, GliF, is presumably involved in the modification of the DKP side chain [30]. In *L*. *maculans*, besides *sirC*, two additional cytochrome P450-encoding genes, *sirB* and *sirE*, are located in the sirodesmin cluster and the *Aspergillus terreus* acetylaranotin cluster contains two cytochrome P450 genes: *ataCT* and *ataF* [45]. In the *C*. *purpurea* ETP cluster only one cytochrome P450 monooxygenase gene (*CPUR_02677*) is present (Table 1). In a phylogenetic tree, *CPUR_02677* (*tcpC*) groups well with *gliC*, *sirC* and *ataTC* (Fig 6), indicating that the enzyme should be responsible for the bishydroxylation of the DKP during ETP biosynthesis in *C*. *purpurea*.

**Fig 7:**
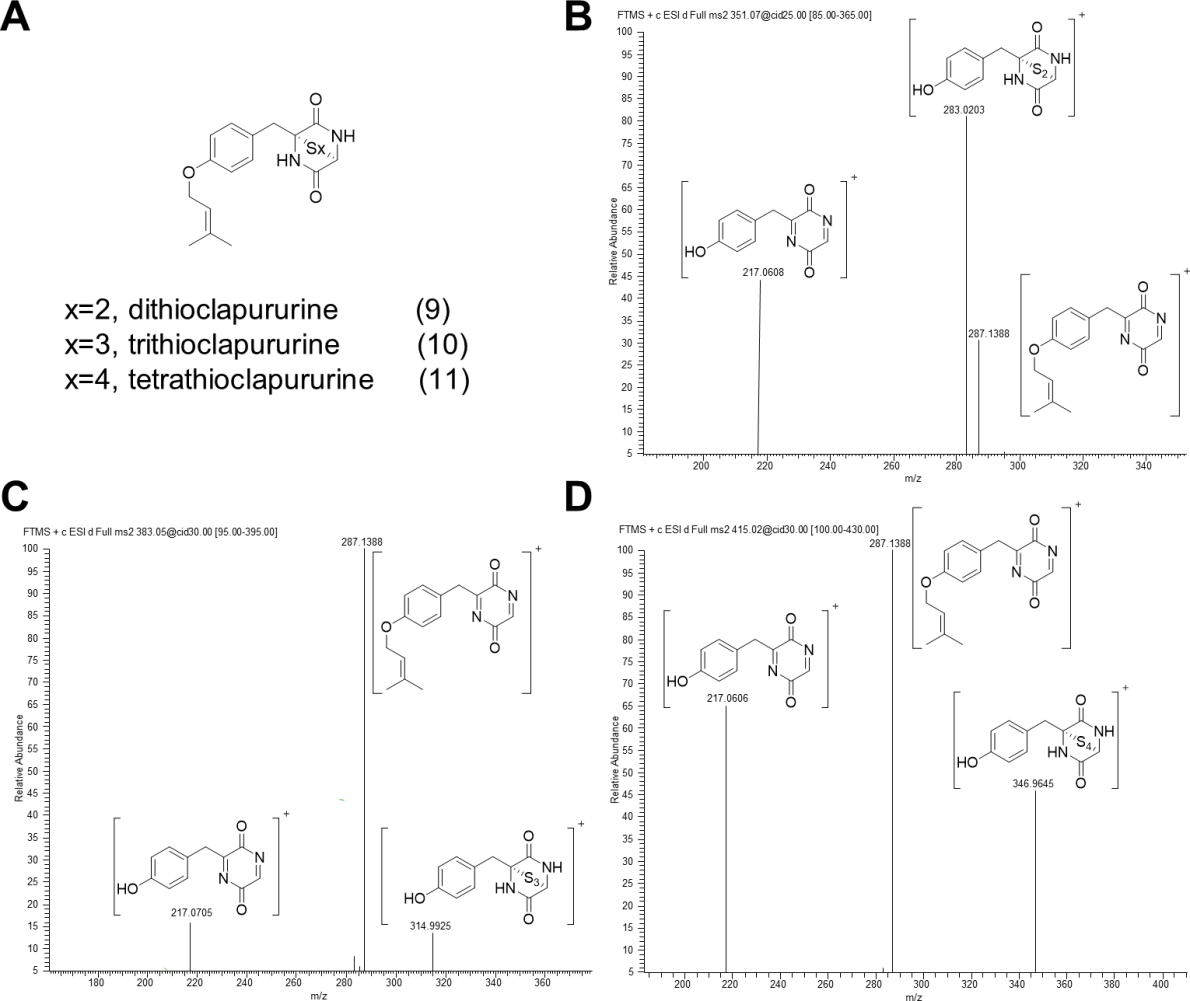
The characteristic MS/MS spectra of the identified clapurines as end-products of the ETP gene cluster in *C*. *purpurea*. **(A)** The clapurines are so far unknown ETPs and were characterized in the OE::*tcpZ*/OE::*sirC* strain. The HRMS^n^ experiments show a neutral loss of the sulfur groups and the common cleavage of the dimethylallyl group for dithioclapurine **(B)**, trithioclapurine **(C)** and tetrathioclapurine **(D)** as [M+H]^+^.

To test if TcpC is the critical enzyme during ETP biosynthesis in *C*. *purpurea*, we expressed the *L*. *maculans* P450 monooxygenase gene *sirC* in the *C*. *purpurea* OE::*tcpZ* strain. Therefore, the *sirC* gene was amplified by PCR from genomic *L*. *maculans* DNA and fused to the strong constitutive *A*. *nidulans* oliC promoter. After transformation in the *C*. *purpurea* OE::*tcpZ* strain, ectopic integration of the vector was checked by PCR and expression of the gene was verified via northern analyses (S3 Fig). The OE::*tcpZ*/OE::*sirC* mutant was cultured in modified Mantle media and the culture filtrate as well as the fungal mycelium was analyzed in the same way as before by HPLC-HRMS. HRMS in positive mode indicated the presence of three new metabolites with the characteristic isotopic sulfur pattern and a molecular formula of **9** (C_16_H_18_N_2_O_3_S_2_), **10** (C_16_H_18_N_2_O_3_S_3_) and **11**°(C_16_H_18_N_2_O_3_S_4_) (Fig 7A).

The molecular formula indicated that the new products only differed in the number of sulfur atoms and the carbon skeleton seemed to be similar to that of *cyclo*-L-tyrosine-L-glycine and a dimethyllalyl group (C_16_H_20_N_2_O_3_). Fig 7B–7D illustrates the HRMS^n^ experiments characterizing the new products. Each metabolite showed the characteristic cleavage of a polysulfur group. The resulting fragment *m/z* 287 with a pseudomolecule formula of C_16_H_18_N_2_O_3_ was in perfect agreement with the already described fragmentation pathway of **1-8** and the HRMS experiments confirmed that the expected sulfur bridge was formed. Thus, by heterologous expression of the cytochrome P450 gene *sirC*, the actual end-products were identified and named as dithioclapurine (**9**), trithioclapurine (**10**) and tetrathioclapurine (**11**). To confirm the predicted structures, the new clapurines were isolated out of the fungal mycelium. Therefore, the fungal mycelium was extracted and the extract directly purified by preparative HPLC. Unfortunately, the concentrations of the metabolites were too low to measure ^13^C spectra. Nevertheless, ^1^H NMR spectra of tetrathioclapurine **11** (0.25 mg) (S14A Fig) exclusively shows the signals of the carbon skeleton as described for **2** above. The proton H-3 could be therefore assigned to the singulet at δ = 5,2 ppm. The more sensitive HSQC experiment assigned the ^13^C signal of C-3 as δ = 60.01 ppm and HMBC couplings between H-7a/b characterized the carbon atom C-6 at δ = 72.05 ppm (S14C Fig).

### *In planta* analysis of the new metabolites

To determine the relevance and occurrence of the new metabolites *in planta*, rye was infected with the *OE*::*tcpZ* and the OE::*tcpZ*/OE::*sirC* strain as well as the *C*. *purpurea* wild-type. No influence of the overproduced metabolites on the infection process of the fungus could be observed. Eight to nine days post-infection first signs of the infection (honeydew production) were visible and after approximately 15 days sclerotia, the resting structure of the fungus, were formed. After three weeks the sclerotia were extracted and analyzed by HPLC-HRMS. The metabolites were identified by their retention time, exact mass and HRMS^n^ data. The spectrum of metabolites in sclerotia was identical to that in axenic culture, including **1a/b**, **2a/b** and **3**. Interestingly, these metabolites were also detectable in wild-type infected sclerotia, but in much lower concentrations. Apart from that, other shunt products were detectable, which did not seem to be connected to the dysfunctional P450 enzyme. In sclerotia of the OE::*tcpZ*/OE::*sirC* strain, additionally dithioclapurine (**10**) was detectable.

In summary, we could show that the standard *C*. *purpurea* strain is not able to produce ETPs with the characteristic disulfide bridge although all genes required are present in the genome. Instead of the expected ETPs we isolated and purified several new, unusual metabolites of the ETP gene cluster in axenic culture and *in planta*. By expressing the *L*. *maculans* P450 monooxygenase-encoding gene *sirC* in *Claviceps* we could demonstrate that this is the critical step in the ETP pathway in *C*. *purpurea*: the actual end-products of the ETP-like gene cluster were identified and characterized by HRMS^n^. The so called clapurines are previously unknown ETPs, which might contribute to the toxicity of other *C*. *purpurea* strains with an intact and functional cytochrome P450 enzyme.

## Discussion

### The ETP gene cluster in *C*. *purpurea*

Fungi are well known for producing a multitude of secondary metabolites. These natural products are of special interest, on the one hand as drugs for the treatment of cancer and infectious diseases, as immunosuppressant’s or as a source of new therapeutic agents [46]. On the other hand, mycotoxins are a risk to human health worldwide and consequently a serious problem for the food and feed industry [47]. The filamentous fungus *C*. *purpurea* is well known for the production of toxic ergot alkaloids. Apart from that, not much is known about other secondary metabolites produced by this important plant pathogen. For this reason, we were interested in exploring the potential of *C*. *purpurea* to produce other secondary metabolites, which may contribute to the toxicity of the fungus. Inspired by their outstanding structure, their strong therapeutic potential and their high toxicity [18] we focused on the putative ETP gene cluster. This gene cluster shows high similarity to the gliotoxin cluster in *A*. *fumigatus* and the sirodesmin cluster in *L*. *maculans* (Table 1). All common ETP moiety genes are highly conserved between the three clusters. However, there are some differences in the genes probably responsible for modification of the ETP side chains, suggesting that the product of the *C*. *purpurea* ETP cluster differs from the so far known ETPs. Putative ETP clusters are present in a diverse range of filamentous fungi, but the presence of such clusters does not always correlate with metabolite production [27]. *C*. *purpurea* is not known as a producer of any ETPs and we were not able to detect any structures with the characteristic ETP disulfide bridge produced by our wild-type reference strain under standard laboratory conditions. Genetic manipulation of global or cluster specific regulators is a common tool to activate silent gene clusters [6,7]. By overexpressing the transcription factor of the ETP cluster in *C*. *purpurea* we were able to activate the whole cluster and to confirm the predicted borders.

### Intermediates of the ETP pathway in *C*. *purpurea*

Uncovering the full chemical potential of a gene cluster is a challenging project, as a single cluster may be responsible for the production of several intermediates and end-products, leading to a complex pool of diverse molecules [48]. In *A*. *fumigatus*, the transcription factor GliZ, is responsible for the production of several gliotoxin related metabolites [34] and also the biosynthesis of other metabolites seems to be influenced by GliZ [33]. Therefore, as expected, the metabolite profile of the *C*. *purpurea* OE::*tcpZ* mutants was quite different from that of the wild-type (Fig 3A and 3B). However, we were able to identify several structures with the expected DKP backbone.

In *A*. *fumigatus* a four-enzyme cascade is responsible for the incorporation of sulfur into gliotoxin [49]. The glutathione S-transferase GliG thiolates the DKP via addition of glutathione [30,50] and GliK, GliJ, and GliI catalyze the successive cleavage of the glutathione molecule [51]. Afterwards, the oxidoreductase GliT mediates the formation of the disulfide bridge [31]. In cultures of the *C*. *purpurea* OE::*tcpZ* mutant, we were not able to detect metabolites with the expected disulfide bridge. Nevertheless, we were able to identify intermediates with homocysteine attached to the α-carbon of the tyrosine part of the DKP (Fig 4, **1**-**2**). Surprisingly, at the carbon of the glycine part of the DKP no glutathione was attached. Instead, the structure contains a methylated nitrogen-sulfur bond. These intermediates provide an explanation for the lack of sulfur bridged metabolites. As the second sulfur at carbon C-3 of the DKP ring is missing, no disulfide bridge can be formed. Probably due to steric effects no formation of a sulfur bridge between the α-carbon and the nitrogen is thermodynamically preferred. Interestingly, when we additionally overexpressed the ETP cluster methyltransferase-encoding gene *tcpN*, the fungus produces structures with a methoxy group at the nitrogen (Fig 5, **4**-**8**). In *A*. *fumigatus* it was shown that the DKP has first to be hydroxylated so that in the next step the sulfur can be incorporated by the attachment of glutathione [40,50]. We thus concluded that in *C*. *purpurea*, the nitrogen of the DKP is hydroxylated. As a result of the high *tcpN* expression in the OE::*tcpZ*/OE::*tcpN* strain (S2C Fig), the hydroxyl group is immediately methylated so that no glutathione can be attached anymore.

In *A*. *fumigatus* the homolog of this methyltransferase (GliN) is responsible for the *N*-methylation of gliotoxin whereas another SAM-dependent methyltransferase, located outside of the ETP cluster, mediates the *S*-methylation of free thiol groups [42,52]. Homologs of those methyltransferase are present in most filamentous ascomycete genomes [52]. However, there seems to be no homolog in *C*. *purpurea*. This would explain the fact that we were not able to detect any substances with methylated sulfur at the α-carbon of the DKP. Scharf et al. (2014) conclude that *S*-methylation is a detoxification strategy to inactivate gliotoxin [42]. The inability of *C*. *purpurea* to methylate the highly reactive thiol group at the α-carbon might explain the S-S bond of compound **7**, where the reactive thiol group formed a disulfide with glutathione. The ability of gliotoxin to form mixed disulfides with thiol groups from proteins or antioxidants like glutathione has already been described in literature [53,54]. The effort to avoid the formation of a free thiol group at C-6 further explains the incomplete glutathione degradation in **5** and **6**. Furthermore, the compounds **1–3** show a *S*-methylation of the nitrogen. The absence of another methyltransferase belonging to the *C*. *purpurea* ETP cluster, implicates that the function of TcpN cannot be limited to *N*-methylation as described for the *A*. *fumigatus* homologe GliN [42] and that the *S*-methylation of the nitrogen can be carried out by TcpN as well. Furthermore, no metabolites show the expected *N*-methylation. That implies that TcpN is singularly specific to methylate the substituted nitrogen.

### The key role of the P450 monooxygenase TcpC

In *A*. *fumigatus* GliC, a cytochrome P450 monooxygenase, is responsible for the bishydroxylation of the DKP so that in the next step the two glutathione molecules could be attached [40]. In *C*. *purpurea* we were able to detect structures with a hydroxyl group at the α-carbon of tyrosine, but never the expected bishydroxylated DKP. Consequently, the second glutathione could not be incorporated and the formation of a disulfide bridge is not possible. We thus conclude that hydroxylation is the critical step during the ETP formation in *C*. *purpurea* and that the functionality of TcpC, the GliC/SirC homolog, is defective. When we expressed the *L*. *maculans sirC* gene in the *C*. *purpurea* OE::*tcpZ* strain, we were able to identify structures with the expected sulfur bridge in axenic culture as well as *in planta*. This clearly shows that bishydroxylation of the DKP is the critical step during ETP biosynthesis in *C*. *purpurea*. In contrast to the *A*. *fumigatus* and *L*. *maculans* ETP clusters, the *C*. *purpurea* cluster contains only one cytochrome P450 monooxygenase gene. However, in a phylogenetic tree (Fig 6), this gene groups well with *gliC* and *sirC*, indicating that the gene should also be responsible for the bishydroxylation of the DKP at the α-carbon positions. Although there are no obvious mutations in the encoding gene sequence leading, for example, to a truncated enzyme, we conclude that the functionality of TcpC is defective due to a mutation in the encoding gene. As a result, TcpC is not able to bishydroxylate the DKP at both α-carbon positions, but hydroxylates the α-carbon of the tyrosine part and the nitrogen of the glycine part. Extensive HPLC-HRMS^n^ measurements at different time stages of growing never showed a DKP molecule with only one substitution at either the α-carbon of tyrosine or the nitrogen, indicating that TcpC is responsible for both hydroxylations simultaneously.

Nevertheless, we cannot rule out the possibility that in *C*. *purpurea* a second cytochrome P450 enzyme, located outside of the ETP cluster and not regulated by TcpZ, is responsible for attaching the hydroxyl group to the second α-carbon. Although there are examples that single genes of a secondary metabolite cluster are located somewhere else in the genome, this possibility is very unlikely. On the one hand because it had been shown in *A*. *fumigatus* that although there is another P450 gene in the cluster, GliC is responsible for the bishydroxylation of the DKP [30]. On the other hand, when a second P450 gene is involved, it has to be somehow co-regulated with the other cluster genes, like e.g. the trichothecene biosynthesis in *Fusarium* species. In this pathway three genes are located outside the cluster but are still under the control of the transcriptional activators Tri10 and Tri6 [55]. The methyltransferase GtmA, located outside of the gliotoxin cluster in *A*. *fumigatus*, is also expressed in the wild-type [52]. *In planta* the *C*. *purpurea* ETP cluster is expressed in the late stages of infection, even in the wild-type. At least under these conditions a hypothetical P450 gene located outside the cluster should be activated. Nevertheless, we were only able to detect small amounts of the main ETP cluster products **1a/b**, **2a/b**, and **3** but no bishydroxylated metabolites. These results additionally argue against a second P450 enzyme involved in the ETP biosynthesis in *C*. *purpurea*.

In *A*. *fumigatus*, it is proposed that GliF, the second P450 enzyme, is involved in the ring closure of gliotoxin. However, the last steps of the biosynthesis are still ambiguous and it has not yet been proven, if GliC or GliF is responsible for this step. The ETP cluster in *C*. *purpurea* contains only one P450 gene and none of the identified cluster products shows a cyclized structure, even when we expressed the *sirC* gene in *Claviceps*. These results give a clear hint that a P450 enzyme other than GliC/SirC is responsible for the ring closure during gliotoxin/sirodesmin biosynthesis.

The loss of function of a secondary metabolite cluster gene is not unusual. In the biotrophic lifestyle of *C*. *purpurea* the production of phytotoxins is not required or even counterproductive for a successful infection process, as the fungus has to avoid host defense reactions. The loss of functionality of genes belonging to a toxin cluster is therefore reasonable from an evolutionary point of view. Furthermore, the fact that the *C*. *purpurea* strain used in this study is not able to produce sulfur bridged end-products does not rule out the possibility that other strains might have an intact ETP cluster. Thus it is possible that some naturally occurring *C*. *purpurea* strains produce toxic ETPs, especially in the late stages of infection when the ETP cluster is expressed (Fig 2A). As in the sclerotia the toxic ergot alkaloids are produced, the co-occurrence of these mycotoxins may lead to an additive or synergistic toxic effect. The knowledge about such additive effects between mycotoxins is still limited, but the increased hazard was already shown, for instance for ochratoxin A and fumonisin B1 [56,57] or ochratoxin A and citrinin [58].

By heterologous expression of *sirC* we were finally able to identify the end-products of the common ETP pathway in *C*. *purpurea*. Surprisingly, *C*. *purpurea* does not only produce the disulfide bridged dithioclapurine but also epitri- and epitetrasulfide (Fig 7A). Such polythio-derivates have already been isolated from fungi. The epidithiodiketopiperazine hyalodendrin was originally isolated from *Hyalodendron* sp. [59], later it was shown that the same fungus also produces tetrathiohyalodendrin [60]. Further examples are the emethallicins A, B and D produced by *Emericella heterothallica* [61,62] or sirodesmin A, B and C, which were first isolated from *Sirodesmium diversum* [63]. Recently an ETP gene cluster in *Aspergillus terreus* was characterized and epitetrasulfides were described as shunt products of the acetylaranotin biosynthesis [45]. An ETP with high similarity to the clapurines is dithiosilvatin, isolated from *Aspergillus silvaticus* [64]. However, in contrast to dithiosilvatin, the clapurines are not *N*-methylated. This is another hint that TcpN is specific to methylate only the substituted nitrogen.

### A proposed biosynthetic pathway

**Fig 8:**
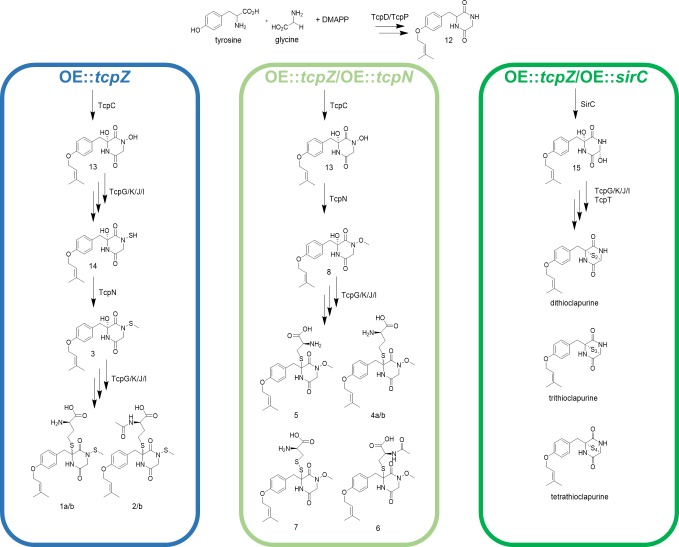
Proposed biochemical pathway for the formation of diketopiperazine metabolites 1-8 and the clapurines. Compounds **12**–**15** are hypothesized intermediates.

In conclusion, the unequivocal structure elucidations of new end-products and intermediates of the ETP-like gene cluster in *C*. *purpurea* are leading to the proposed biosynthetic pathway presented in Fig 8.

Firstly, L-tyrosine is prenylated, as described already for sirodesmin PL [38] then undergoes condensation with L-glycine in a reaction catalyzed by the NRPS TcpP leading to the DKP **12.** However, it is not clear if L-tyrosine is first transferred to the NRPS and later prenylated or vice versa. Afterwards the α-carbon of tyrosine is oxidized by the cytochrome P450 TcpC to form a hydroxyl group. The latter one further catalyzes the hydroxylation of the nitrogen N-4 to form the hypothetical intermediate **13** in the next step. In the OE::*tcpZ* strain the hydroxyl group at N-4 is protonated to form a perfect leaving group which is enzymatically substituted by glutathione. Then TcpI catalyzes an α,β-elimination reaction to afford a free thiol group (**14**) in the same way as described in the biosynthesis of gliotoxin [30,49]. The highly reactive thiol group might be rapidly methylated by the methyltransferase TcpN to produce **3**. In the OE::*tcpZ*/OE::*tcpN* strain the hypothesized hydroxyl group of the intermediate **13** is rapidly methylated by the overexpressed methyltransferase TcpN to form **8**. As a result of that, the formation of the unique nitrogen-sulfur bond is inhibited. Furthermore, the overexpressed methyltransferase seems to be involved in the glutathione and homocysteine metabolism. Therefore, homocysteine (**4**) as well as glutathione residues (**5**, **6** and **7**) are occurring. In case of the biosynthesis of the clapurines, the heterologously expressed enzyme SirC is responsible for the hydroxylation of **12** producing the previously described intermediate analogue **15 **[30] which is further converted to form the clapurines **9**–**11**. To elucidate the full biosynthetic pathway of these unusual new metabolites further knock-outs of cluster genes were useful, but due to the low gene targeting efficiency (about 1-2%) [65] gene deletion is rather challenging and time consuming in *C*. *purpurea*.

**Fig 9:**
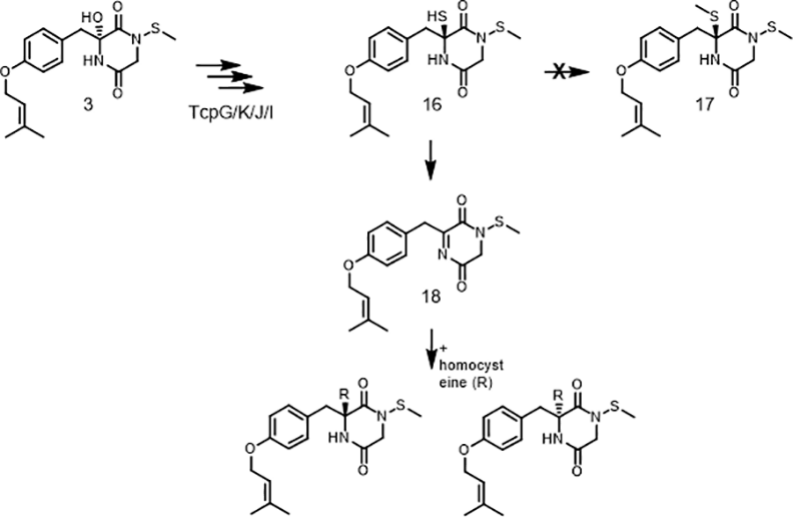
Tentative biochemical pathway for the diastereomers 1a/b and 2a/b. Compounds **16** and **18** are hypothesized intermediates.

The metabolites **1a/b**, **2a/b** and **4a/b** are differing from all other metabolites of OE::*tcpZ* as well as OE::*tcpZ*/OE::*tcpN* strains in their occurrence as diastereomers at C-6. This indicates that no enzymatic reaction takes place. For many ETPs a biologically inactive bisthiomethylated derivate is known [18,66,67], showing no toxicity anymore [68]. Furthermore, in case of gliotoxin it is shown that the methylated compound strongly suppresses the biosynthesis of gliotoxin [52]. However, the necessary methyltransferase to form the hypothesized metabolite **17** is missing in *C*. *purpurea*. Therefore, a plausible biosynthetic pathway (Fig 9) for the homocysteine metabolites is firstly the formation of **3** in the already described way (Fig 8). Next, catalyzed by the four enzyme cascade tcpG/K/J/I [49] the highly reactive and unstable metabolite **16** is formed. A subsequent rapid elimination of H_2_S leads to an imine intermediate (**18**), as described in literature for gliotoxin [30,69]. Consequently, a nucleophilic substitution by homocysteine explains the presence of diastereomers at C-6.

## Conclusions

Our results illustrate the key role of the cytochrome P450 monooxygenase TcpC for the biosynthesis of ETPs in the reference strain of *C*. *purpurea*. Due to a dysfunctional enzyme the fungus is not able to produce these toxic metabolites. Instead, rather unexpected diketopiperazine intermediates with an unusual nitrogen-sulfur bond were formed. By heterologous expression of the *L*. *maculans sirC* gene we were able to identify the ETP cluster end-products in *C*. *purpurea*; the so far unknown ETPs di-, tri- and tetrathioclapurine. Our results demonstrate how a mutation of one cluster gene can lead to the accumulation of probably nontoxic metabolites without a reactive thiol group instead of the production of toxic ETPs.

## Materials and Methods

### Strains and culture conditions

The wild-type *Claviceps purpurea* (Fr.) Tul strain 20.1 [70] used in this study, is a benomyl-treated putative haploid derivative of the standard field isolate T5 (Fr.:Fr.) Tul., isolated from *Secale cereale* (Hohenheim, Germany). For DNA isolation mycelia were grown on complete medium BII [71] and for harvesting conidia on Mantle medium [72]. For RNA isolation and chemical analyses strains were cultivated in 50 mL liquid Mantle medium [72] on a rotary shaker at 26°C and 180 rpm. For culturing strain OE::*tcpZ*/OE::*sirC* Mantle medium supplemented with 0.01 g/L glutathione and 0.05 g/L pyridoxal phosphate was used. For upscaled cultures, strains were cultivated in 100 mL medium at 26°C for 12 days and the culture filtrates were combined afterwards.

Vector construction using the yeast recombinational method was performed in the yeast strain FY834 [73]. Yeast strains were incubated at 30°C in yeast extract-peptone-dextrose (YPD) or in synthetic dextrose (SD) medium lacking the selecting amino acids.

### Chemical and Materials

All chemicals were purchased from Sigma-Aldrich GmbH (Seelze, Germany), Carl Roth GmbH + Co. KG (Karlsruhe, Germany) or VWR International GmbH (Darmstadt, Germany). Solvents were obtained in gradient grade quality. Water for HPLC was purified by a Milli-Q Gradient A 10 system (Millipore, Schwalbach, Germany).

### Nucleic acid extraction and analysis

Genomic fungal DNA was prepared from lyophilized mycelium as described by Cenis [74]. For Southern blot analysis, 5 to 10 μg of digested genomic DNA were separated via gel electrophoresis in a 1% agarose gel with salt-free buffer [75] and transferred to a nylon membrane (Nytran SPC; Whatman). 32P-labeled probes were generated using the random oligomer-primer method and hybridized to the membranes overnight at 65°C. For the isolation of RNA the RNAgents total RNA isolation kit (Promega GmbH, Mannheim, Germany) was used. For northern blotting samples of 20 mg were used for the separation on a 1% (w/v) agarose gel containing 1% (v/v) formaldehyde. The separated RNA was transferred to nylon membranes (Nytran SPC; Whatman). Using the random oligomer-primer method, 32P-labeled probes were generated and hybridized to the membranes overnight at 65°C. PCR reactions were performed using the BioTherm Taq DNA Polymerase (GeneCraft, Germany) for diagnostic applications and the proof reading Phusion DNA polymerase (Finnzymes, Finland) for amplification of overexpression vector fragments. Primers were synthesized by Biolegio (Nijmegen, Netherlands).

### Vector construction

Vectors were constructed based on the described vector system [76,77] using the yeast recombinational cloning method [78]. For construction of the *tcpZ* overexpression vector, the *tcpZ* gene (*CPUR_02671)* was amplified with Phusion polymerase with the primers OE_Cp2671_F and OE_Cp2671_R from genomic DNA and recombined with the *Not*I-*Nco*I-digested pNAH-OGG vector [77]. For construction of the *tcpN* overexpression vector, *tcpN* (*CPUR_02672*) was amplified with the primers OE_Cp2672_F and OE_Cp2672_R from genomic DNA using Phusion polymerase and recombined with the *Not*I-*Nco*I-digested pNDB-OGG vector [77]. For construction of the *sirC* overexpression vector, the sirC gene was amplified with the primers OE_sirC_F and OE_sirC_R from genomic *L*. *maculans* JN3 DNA using Phusion polymerase and recombined with the *Not*I-*Nco*I-digested pNDB-OGG vector [77].

For construction of the *tcpT* replacement vector, the flanking regions of *tcpT* (*CPUR_02681*) were amplified with the primers 2681_5F and 2681_5R for the 5′ flank as well as 2681_3F and 2681_3R for the 3′ flank, containing overlapping sequences toward the yeast shuttle-vector pRS426 or the phleomycin resistance cassette. The phleomycin resistance cassette was amplified with the primers CpBle1F and CpBle1R from pRS426CpBle. The yeast shuttle vector pRS426 [78] was linearized by restriction with *Xho*I and *Eco*RI.

For homologous recombination the vector fragments were transformed into yeast strain FY834. The resulting vectors were selected on SD medium lacking uracil. DNA was isolated from yeast cells using the SpeedPrep yeast plasmid isolation kit (DualSystems) and transformed into *Escherichia coli* TOP10’ for amplification.

### Fungal transformation

Protoplasts of *C*. *purpurea* generated with lysing enzymes from *Trichoderma harzianum* (Sigma-Aldrich, St. Louis) were transformed with 10 μg of vector DNA as described by Jungehülsing et al. [79]. For selection either phleomycin was directly applied to the protoplasts (33 μg/mL) or hygromycin was applied to regenerated protoplasts 24 h after transformation by overlay agar at a concentration of 1.5 mg/mL. Resistant colonies were transferred to fresh selective medium (BII, pH 8, with phleomycin at 100 μg/mL or hygromycin at 0.5 mg/mL). Resulting resistant transformants were checked for the integration of the vector by PCR using specific primers as indicated.

### Pathogenicity assays

Male sterile rye plants (*Secale cereale*) were cultivated in growth chambers as described by Smit and Tudzynski [80]. Florets of blooming ears (30 to 40 florets per ear) were inoculated with 5 μL of a suspension containing 2 × 10^6^ conidia/mL collected from Mantle agar, as described by Tenberge and colleagues [81]. To avoid cross contaminations, the ears were covered with paper bags equipped with cellophane windows directly after inoculation.

### qRT-PCR

RNA was isolated by using the RNAgents total RNA isolation kit (Promega GmbH, Mannheim, Germany). For reverse transcription of the RNA template, Superscript II reverse transcriptase (Invitrogen, Darmstadt, Germany) was used. Real-time qPCR reactions were performed with the Bio-Rad iQ SYBR Green Supermix and the iCycler Thermal Cycler (Bio-Rad, Hercules, CA, U.S.A.). Programming, data collection, and analyses were performed with the iCycler iQ Real-Time Detection System Software (version 3.0; Bio-Rad). Expression of *CPUR_02679* was detected by the primers RTq_LN3_F2 and RTq_LN3_R2. The expression of all analyzed genes was normalized to the expression of the housekeeping genes encoding β-tubulin (CCE34429.1), γ-actin (AEI72275.1), and glyceraldehyde-3-phosphate dehydrogenase (X73282.1) [82] using primers Actin_uni and Actin_rev, Tub_uni and Tub_rev, and Gpd_uni and Gpd_rev. Expression was verified in three independent biological replicates.

### Identification of new intermediates by RP-HPLC-DAD-HRMS

In order to identify biosynthetic pathway intermediates, the culture filtrates were measured by RP-HPLC-DAD-HRMS. For the HRMS measurement, an Accela LC 60057-60010 system (Thermo Fisher Scientific, Bremen, Germany) was linked to a LTQ Orbitrap XL mass spectrometer (Thermo Fisher Scientific). A SPD-M20A Shimadzu PDA Detector (Shimadzu, Duisburg, Germany) was coupled to the MS spectrometer. Data acquisition was performed with Xcalibur 2.07 SP1 (Thermo Scientific). Separation was carried out on a 150 x 2.1, 3 μm, Nucleodur phenyl-hexyl column (Macherey-Nagel, Düren, Germany) using a binary gradient at a column temperature of 40°C. The injection volume was 15 μL and the autosampler was cooled to 7°C. The flow rate was set to 250 μL/min. Solvent A was MeOH with 1% of formic acid (v/v) and solvent B was water with 1% of formic acid (v/v). The HPLC was programmed as follows: in the first 5 minutes isocratic 10% of A, afterwards a binary gradient to 100% in 35 min. Then the column was washed with 100% A and equilibrated at starting conditions. For the detection of intermediates, a total ion scan of a mass range from *m/z* 100–800 with a resolution of 60 000 in the positive and negative ion mode was used. All observed ions showed errors less than 5 ppm. The HRMS data were analyzed by Metworks (Thermo Fisher Scientific, Bremen, Germany) in order to screen for the characteristic sulfur isotopic pattern. To get information on the structure of the novel metabolites, the new substances were analyzed by stepwise mass spectrometric fragmentation experiments in the positive mode. The experiments included collision-induced dissociation (CID) with a relative energy of 20% to 45%, depending on the ionization and an isolation width of *m/z* 1.5. The fragments were analyzed with the Orbitrap detector at a resolution of 60 000.

### Analysis of sclerotia

After three weeks the sclerotia were ground and extracted with a mixture of ethyl acetate and water as well as acidified ethyl acetate. 500 μL water and 500 μL of organic solvent were added to the ground sclerotia in a 1.5 mL E-cup and shaken for 15 min. In the next step, phases were separated and the solvent evaporated. Afterwards resolved in 500 μL methanol/water (1+9, v/v) and 10 μL used for injection. The three extracts (ethyl acetate/ acidified ethyl acetate/ water) were analyzed in the same way as in the axenic cultures by RP-HPLC-DAD-HRMS. The metabolites were identified by their retention time, exact mass and HRMS^n^ data.

### Isolation

The liquid fungal culture was filtrated through Miracloth (Calbiochem, Merck, Darmstadt, Germany) to remove the mycelium. 1 L of this filtrate was fractionated on a Strata C18-E 55 μm, 70 Å, 10 g/60 mL solid phase extraction cartridge (Phenomenex, Aschaffenburg, Germany). First, the cartridge was activated by use of 150 mL methanol and 200 mL water. All steps were performed under vacuum at the column outlet. The culture filtrate was applied to the cartridge and to prevent plugging of the column 100 mL methanol/water (5+95, v/v) were applied in between to wash off salts and sugars and other polar compounds. After application of the whole amount of filtrate it was again washed with 200 mL of water. Afterwards five fractions with different amounts of methanol/water were collected (each 200 mL). The fractions were as follows: 20%, 40%, 60%, 80% and 100% methanol/water (v/v). An aliquot of the five fractions were diluted 1:10 with water and used for fraction control via HPLC-HRMS. Therefore, the same MS-parameters were used as described above. For further steps only the 60% and 80% fractions containing the relevant metabolites were used. The solvent of these fractions was removed using a rotary evaporator (Büchi Rotavapor R-110 with Büchi Waterbath B-480, Büchi Labortechnik, Essen, Germany) at a temperature of 40°C under vacuum. Then the samples were dried using a freeze-drier LyoVac GT2 (AMSCO Finn-Aqua, Hürth, Germany) and resolved in 15 mL methanol/water (40+60, v/v). In the next step the fractions were further purified using preparative HPLC-UV. All laboratory work was carried out under reduced light conditions and every flask or tube was wrapped with aluminum foil. All samples or fractions were stored at -25°C.

### Extraction and Isolation of the fungal mycelium

The biomass was suspended in 200 ml of water and cell disruption was carried out with an Ultra-Turrax (Jahnke and Kunkel, Germany) under cooling with ice. The rotor speed was 12 000 rpm. Afterwards the mycelium was extracted several times with a mixture of n-pentan and methyl-t-butyl ether. The organic solvents were evaporated, and the extract directly purified by preparative HPLC-UV.

### Preparative HPLC-UV

For the isolation and purification a Jasco HPLC-UV system (Jasco, Groß-Umstadt, Germany) was used. The system consists of PU-2087 pumps (Jasco), a dynamic mixing chamber (Knauer, Berlin, Germany) and a degasys populaire DP 4010 degasser (Uniflows, Tokyo, Japan) and system controller LC-NetII/ADC (Jasco). Injection was performed manually via rheodyne model 7125 with a 2 mL sample loop. The detector was an UV-2075 UV/Vis detector (Jasco), the installed software was Jasco-Borwin, Version 1.5 (Jasco). The column used was a 250 mm x 10 mm, 5 μm Nucleodur phenyl-hexyl column (Macherey-Nagel, Düren, Germany) with a 4.0 mm x 2.0 mm phenyl-hexyl guarding column (Phenomenex, Aschaffenburg, Germany). Solvent A was methanol with 1% of formic acid (v/v) and solvent B was water with 1% of formic acid (v/v). The gradient was as follows: 10 min at 40% of A and afterwards a binary gradient to 80% of A during 40 min. Afterwards the column was washed with 100% A and equilibrated at starting conditions. The injection volume was 500 μL and the detection wavelength was set to 260 nm.

### Mass spectrometric structure elucidation

An aliquot of 20 μL of each isolate obtained after preparative HPLC was 1:10 diluted with methanol/water (1+9, v/v) and analyzed via RP-HPLC-HRMS. The column and solvent were the same as described above. The HPLC-parameters were as follows: 2 min with 20% of A isocratically. Then a binary gradient for 8 min to 100% of A. Afterwards the column was washed with 100% A and then equilibrated. The flow rate was set to 250 μL/min. To get information on the structure of the novel metabolites, the new substances were analyzed by stepwise mass spectrometric fragmentation experiments in the positive mode. The experiments included collision-induced dissociation (CID) with a relative energy of 20% to 48%, depending on the ionization of the ions, and an isolation width of *m/z* 1.5. The fragments were analyzed with the Orbitrap detector at a resolution of 60 000. The molecular composition was assigned on base of the accurate mass of the [M+H]^+^- and [M+Na]^+^ ions with a mass tolerance of 5.0 ppm or less.

### Mass spectrometer and DAD parameters

The LTQ Orbitrap XL was used with a heated electrospray ionization technique. The sheat gas flow was 40 arbitrary units, the aux gas flow 20 arbitrary units and the sweep gas flow 5 arbitrary units. In the positive mode, vaporizer temperature was set to 350°C and capillary temperature to 285°C. The source voltage was 3.5 kV, capillary voltage 43 V and Tube Lens 145 V. In the negative mode, vaporizer temperature was set to 300°C and capillary temperature to 285°C. The source voltage was 3 kV, the capillary voltage -35 V and the Tube Lens -110 V.

The Shimadzu PDA-Detector had the following parameters: starting wavelength 200 nm, ending wavelength 600 nm, with a wavelength step of 4 nm. The sampling frequency was consequently 4.16 Hz.

### Neutral loss experiments by HPLC-MS/MS

The mass spectrometer used was an AB SCIEX QTRAP 5500 (Darmstadt, Germany) with ESI. The curtain gas was set to 40 psi, the collision activated dissociation gas to medium, GS1 to 30 psi, and GS2 to 45 psi. The source temperature was 450°C. The ion spray voltage was 5500 V in positive mode and 4500 V in negative mode. Entrance potentials of 10 V and -10 V were used in positive and negative modes, respectively. Unit resolution was applied and a neutral loss of 64 Da measured. The scan rate was set to 200 Da/s. The same chromatography as for the HRMS screenings was used.

## Supporting Information

S1 Fig Verification of genomic presence of the *tcpZ* overexpression construct. Transformants with ectopic integration of the overexpression vector (A) were identified via PCR (B) using primer pair PoliC_F and Tgluc_R.
(PDF)

S2 Fig Verification of genomic presence of the *tcpN* overexpression construct. To generate double overexpression transformants of *tcpZ* and *tcpN*, the *tcpN* overexpression vector (A) was transformed into the OE::tcpZ transformant T2. Transformants with ectopic integration of the *tcpN* overexpression vector were identified via PCR (B) using primer pair PoliC_F and cp2672_R1 (5´), and Tgluc_R and cp2672_F1 (3´). Overexpression of *tcpC* as well as *tcpN* was verified by northern analysis (C).
(PDF)

S3 Fig Generation of *sirC* overexpression mutants. To generate double overexpression transformants of *tcpZ* and *sirC*, the *sirC* overexpression vector (A) was transformed into the OE::*tcpZ* transformant T2. Overexpression of *sirC* was verified by northern analysis (B).
(PDF)

S4 Fig Molecular structures of compounds 1–11. (PDF)

S5 Fig NMR analysis of compound 1a. (A) ^1^H-NMR, (B) H,H-COSY, (C) HSQC.
(PDF)

S6 Fig NMR analysis of compound 1b. (A) ^1^H-NMR, (B) H,H-COSY, (C) HSQC.
(PDF)

S7 Fig NMR analysis of compound 2a. (A) ^1^H-NMR, (B) ^13^C-NMR, (C) H,H-COSY, (D) HSQC, (E) HMBC.
(PDF)

S8 Fig NMR analysis of compound 2b. (A) ^1^H-NMR, (B) ^13^C-NMR, (C) H,H-COSY, (D) HSQC, (E) HMBC.
(PDF)

S9 Fig NMR analysis of compound 3. (A) ^1^H-NMR, (B) ^13^C-NMR, (C) H,H-COSY, (D) HSQC, (E) HMBC.
(PDF)

S10 Fig NMR analysis of compound 4a. (A) ^1^H-NMR, (B) ^13^C-NMR, (C) H,H-COSY, (D) HSQC, (E) HMBC.
(PDF)

S11 Fig NMR analysis of compound 5. (A) ^1^H-NMR, (B) ^13^C-NMR, (C) H,H-COSY, (D) HSQC, (E) HMBC.
(PDF)

S12 Fig NMR analysis of compound 6. (A) ^1^H-NMR, (B) ^13^C-NMR, (C) H,H-COSY, (D) HSQC, (E) HMBC.
(PDF)

S13 Fig NMR analysis of compound 7. (A) ^1^H-NMR, (B) HMBC.
(PDF)

S14 Fig NMR analysis of compound 11. (A) ^1^H-NMR, (B), H-H COESY, (C) HSQC, (D) HMBC.
(PDF)

S15 Fig HRMS^n^ spectra of compounds 2a/b, 3, 5, 6, 7, and 8 as [M+H]^+^ or [M+Na]^+^. (PDF)

S1 Table Oligonucleotide primers used in this study. (PDF)

S2 Table Comparison of the ^13^C NMR signals of compound 1/ab, 2a/b, 3, 4a, 5,6, 7 and tetrathioclapurine (11). The structures are depicted in S4 Fig.
(PDF)

S1 File NMR spectroscopy. (PDF)
