# Supplementary figures and images for: The Epipolythiodiketopiperazine Gene Cluster in Claviceps purpurea: Dysfunctional Cytochrome P450 Enzyme Prevents Formation of the Previously Unknown Clapurines
Source: PLoS One. 2016 Jul 8;11(7):e0158945. doi: 10.1371/journal.pone.0158945 (PMC4938161; doi:10.1371/journal.pone.0158945)

**A**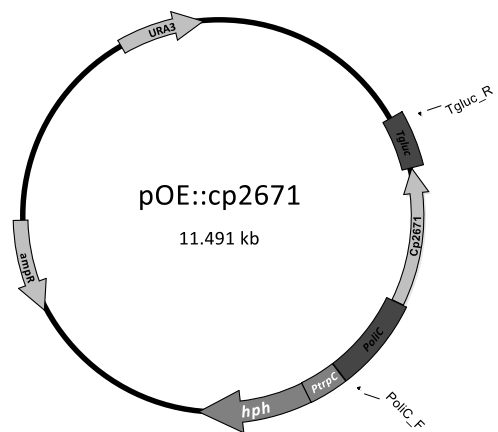**B**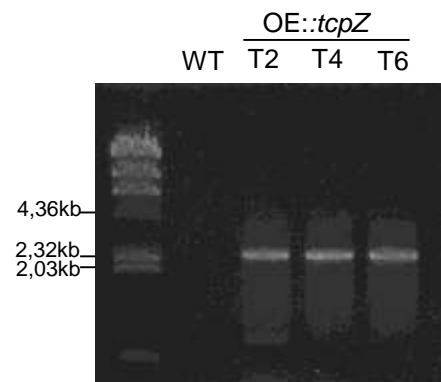

Supplement: S1 Fig — Transformants with ectopic integration of the overexpression vector (A) were identified via PCR (B) using primer pair PoliC_F and Tgluc_R. (PDF) [file pone.0158945.s001.pdf]

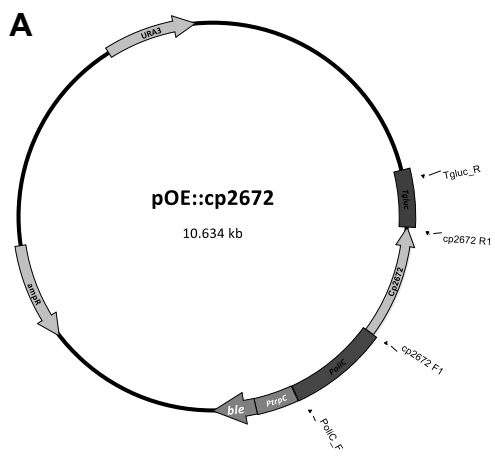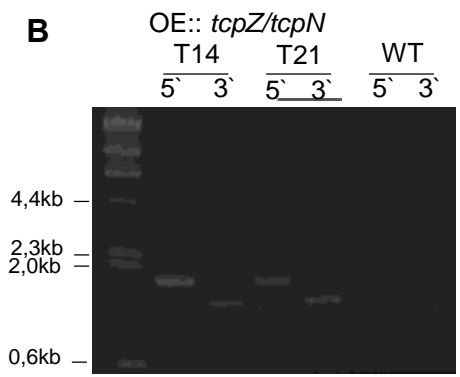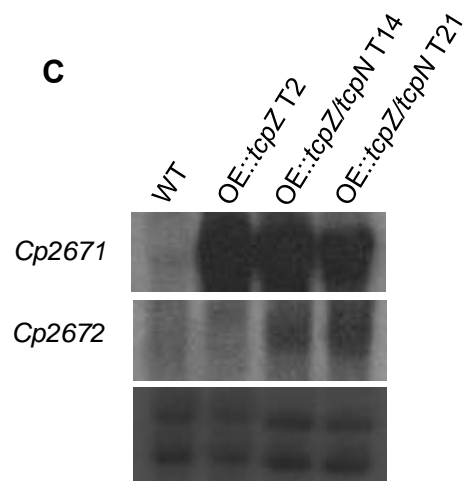

Supplement: S2 Fig — To generate double overexpression transformants of tcpZ and tcpN, the tcpN overexpression vector (A) was transformed into the OE::tcpZ transformant T2. Transformants with ectopic integration of the tcpN overexpression vector were identified via PCR (B) using primer pair PoliC_F and cp2672_R1 (5´), and Tgluc_R and cp2672_F1 (3´). Overexpression of tcpC as well as tcpN was verified by northern analysis (C). (PDF) [file pone.0158945.s002.pdf]

**A**

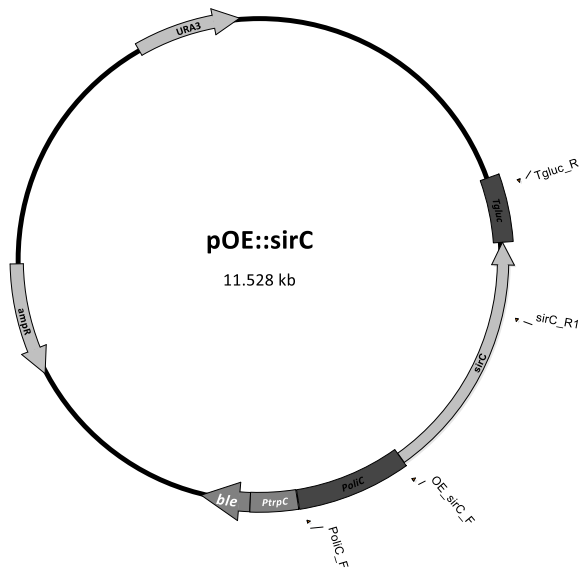

**B**

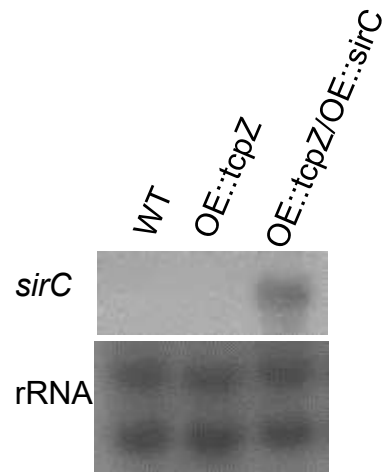

Supplement: S3 Fig — To generate double overexpression transformants of tcpZ and sirC, the sirC overexpression vector (A) was transformed into the OE::tcpZ transformant T2. Overexpression of sirC was verified by northern analysis (B). (PDF) [file pone.0158945.s003.pdf]

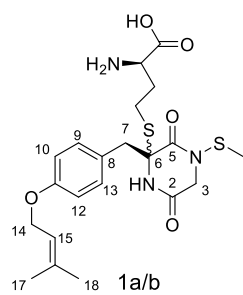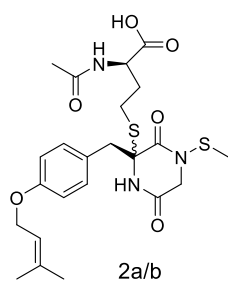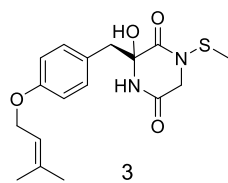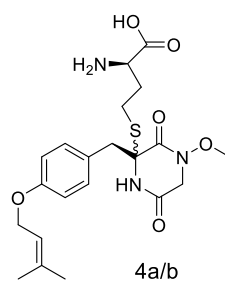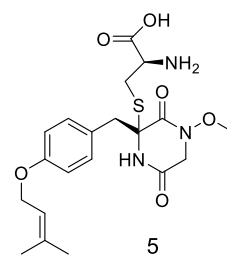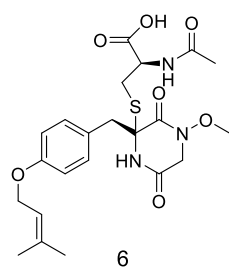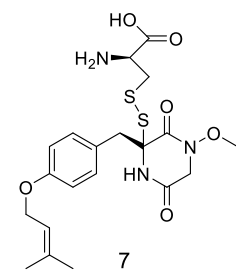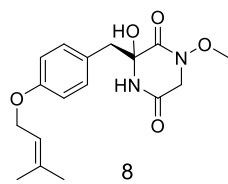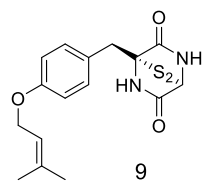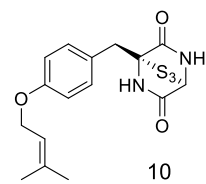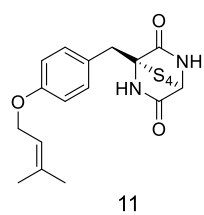

Supplement: S4 Fig — (PDF) [file pone.0158945.s004.pdf]

**A**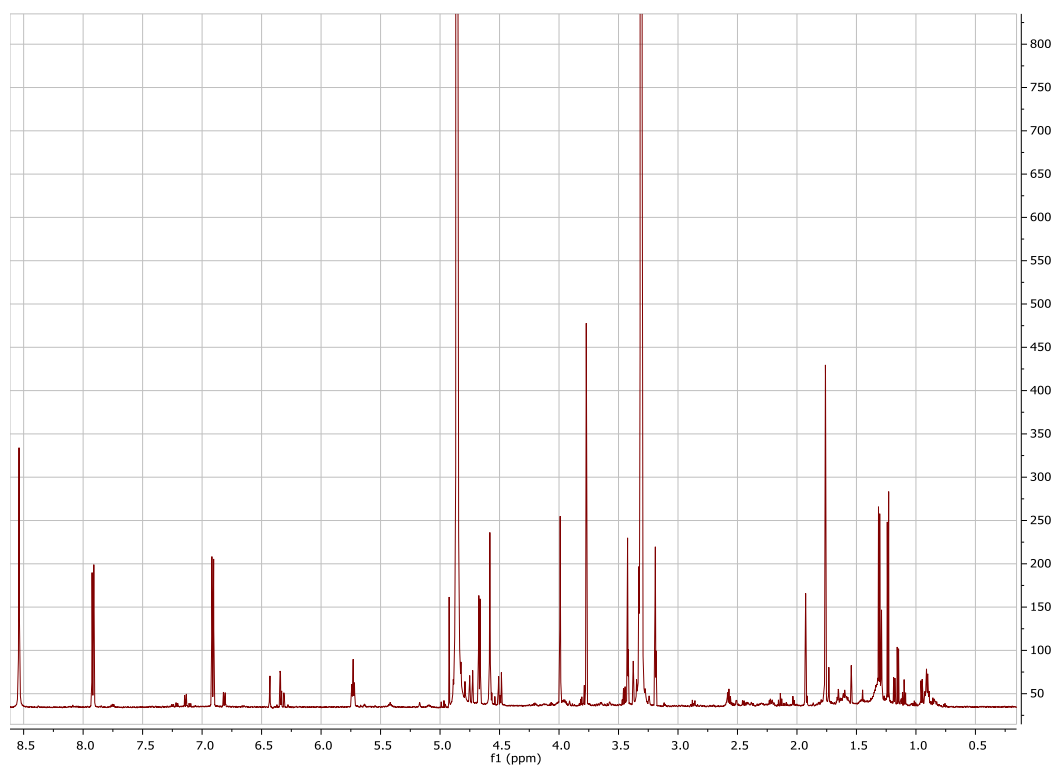**B**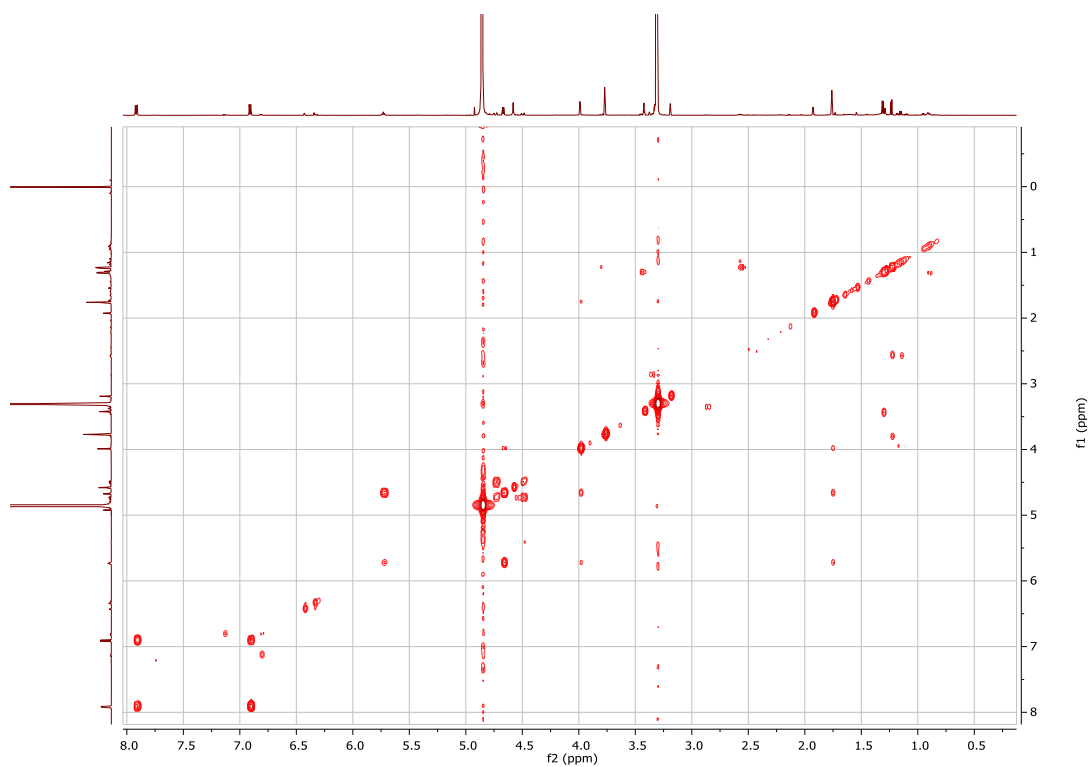

**c**

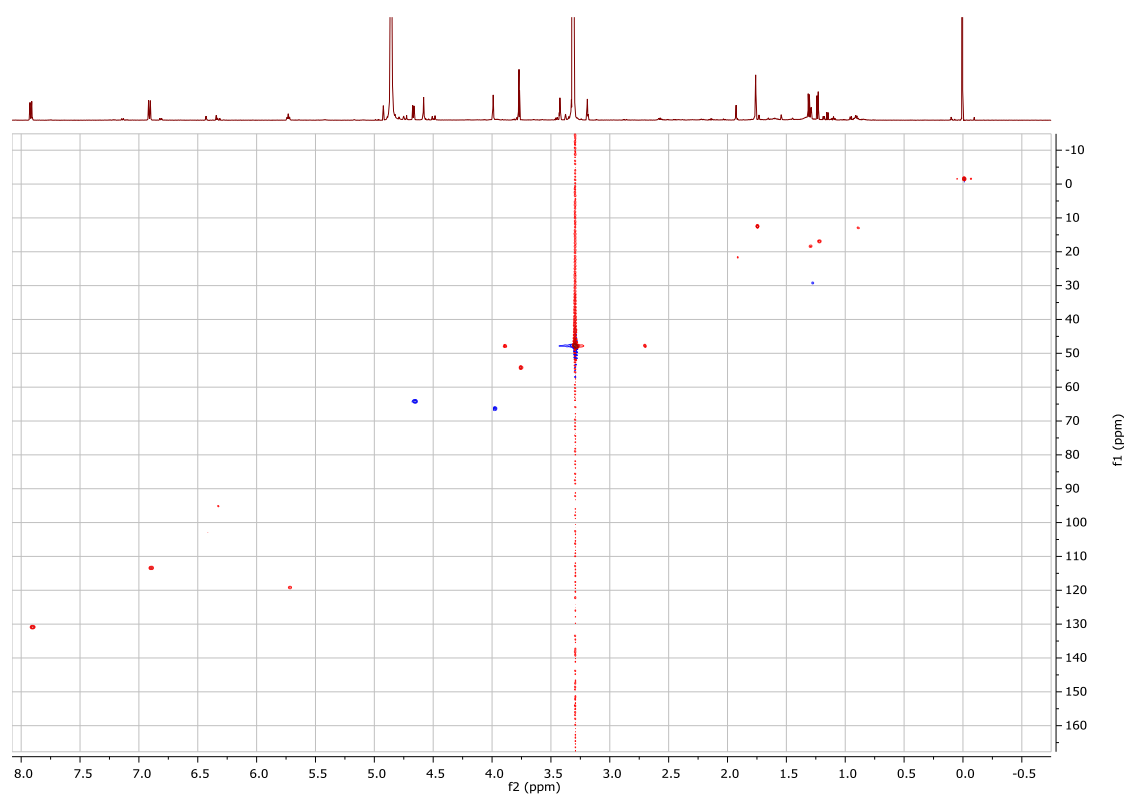

Supplement: S5 Fig — (A) 1H-NMR, (B) H,H-COSY, (C) HSQC. (PDF) [file pone.0158945.s005.pdf]

**A**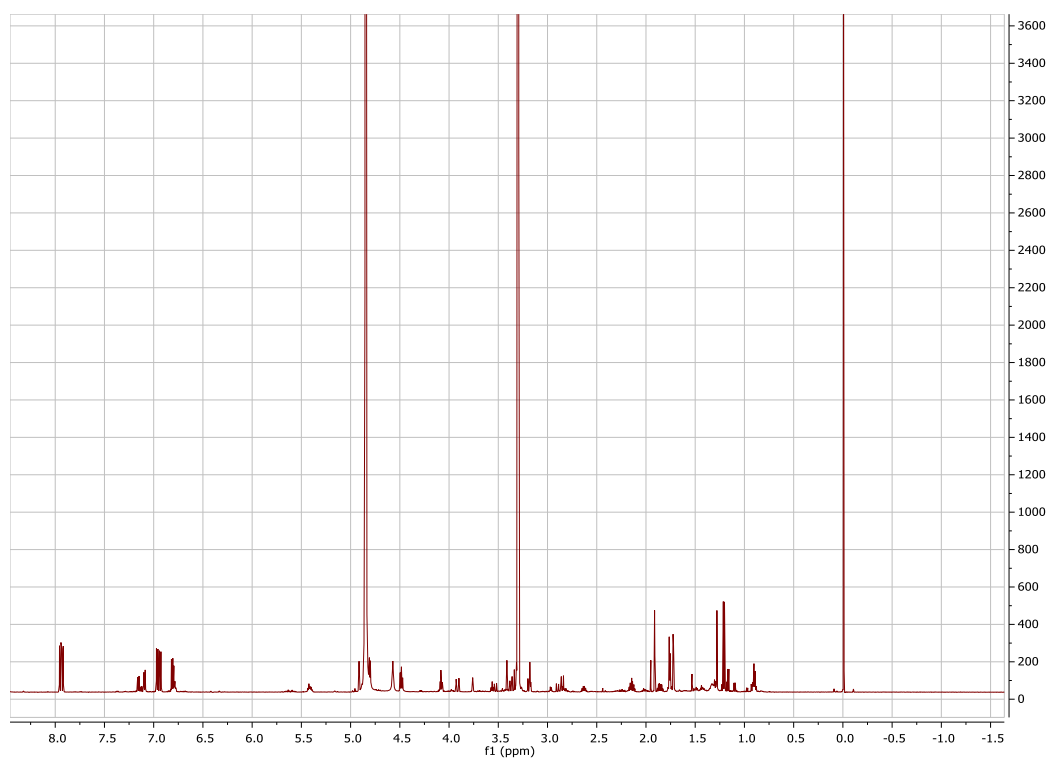**B**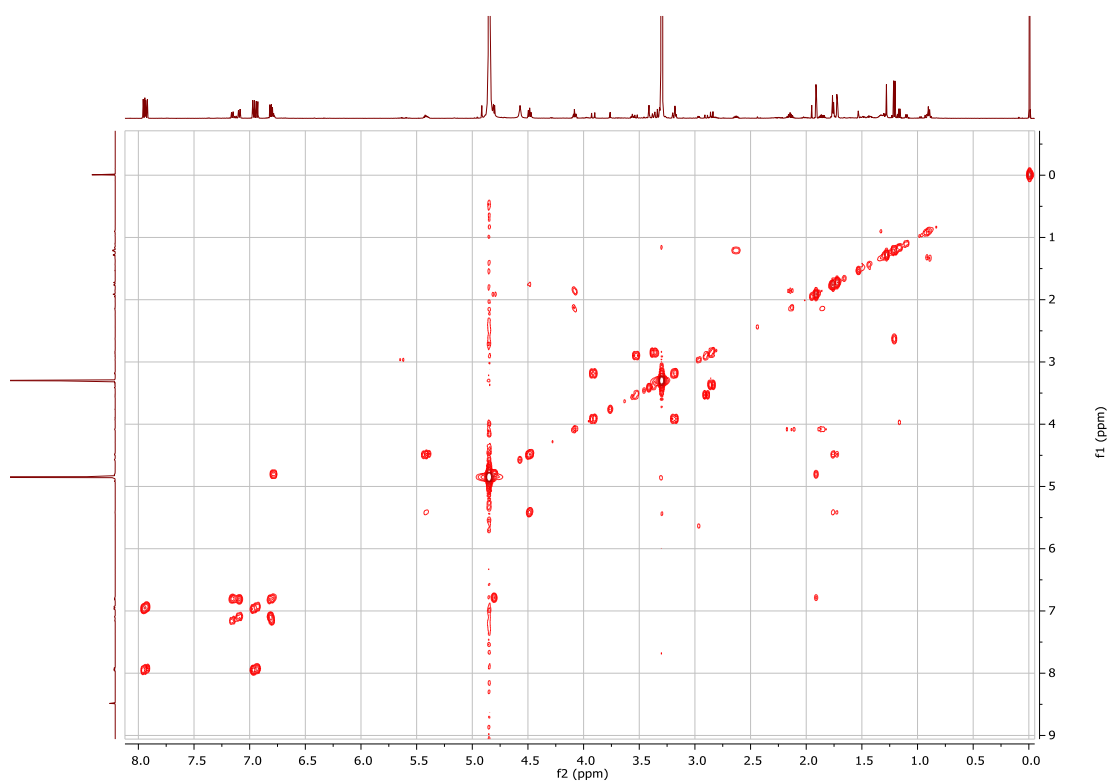

**C**

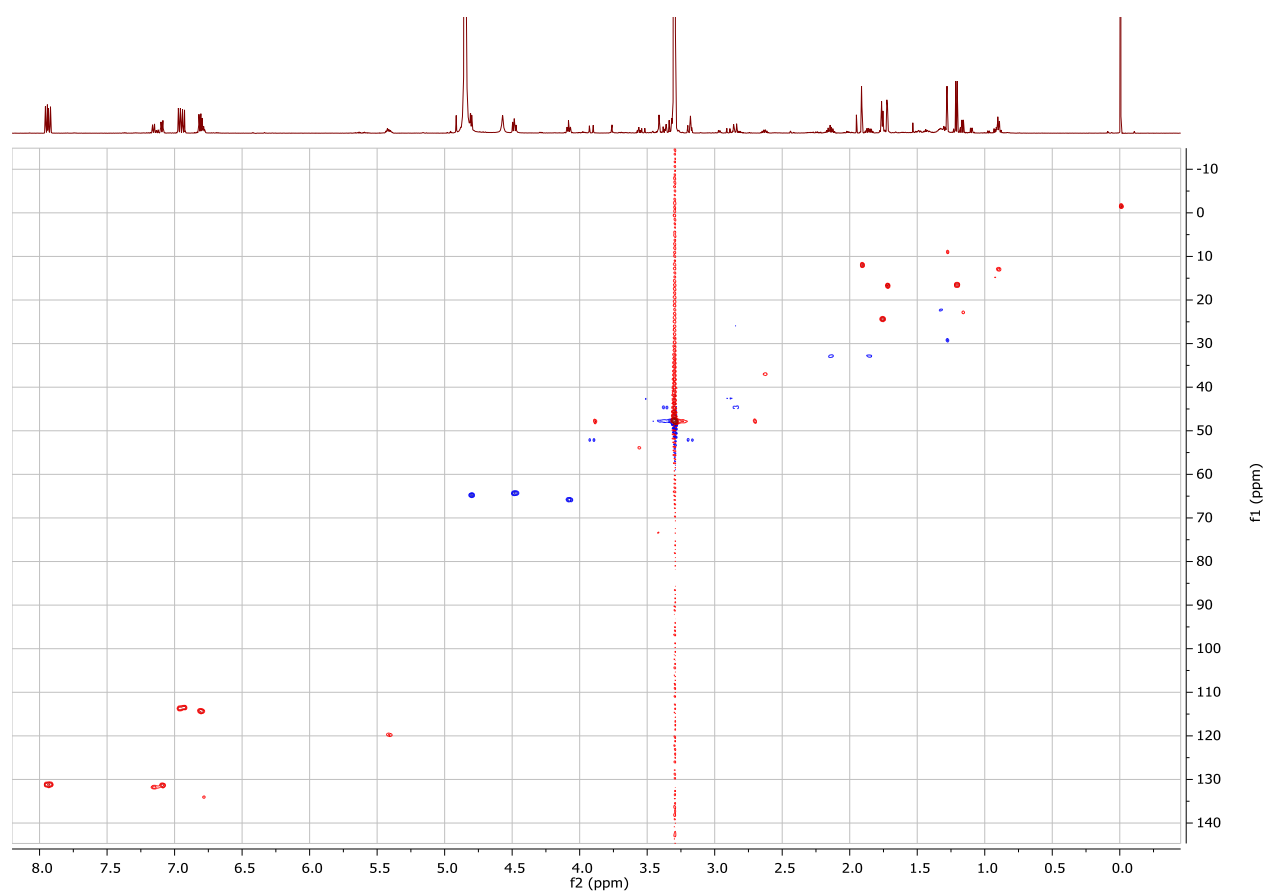

Supplement: S6 Fig — (A) 1H-NMR, (B) H,H-COSY, (C) HSQC. (PDF) [file pone.0158945.s006.pdf]

**A**

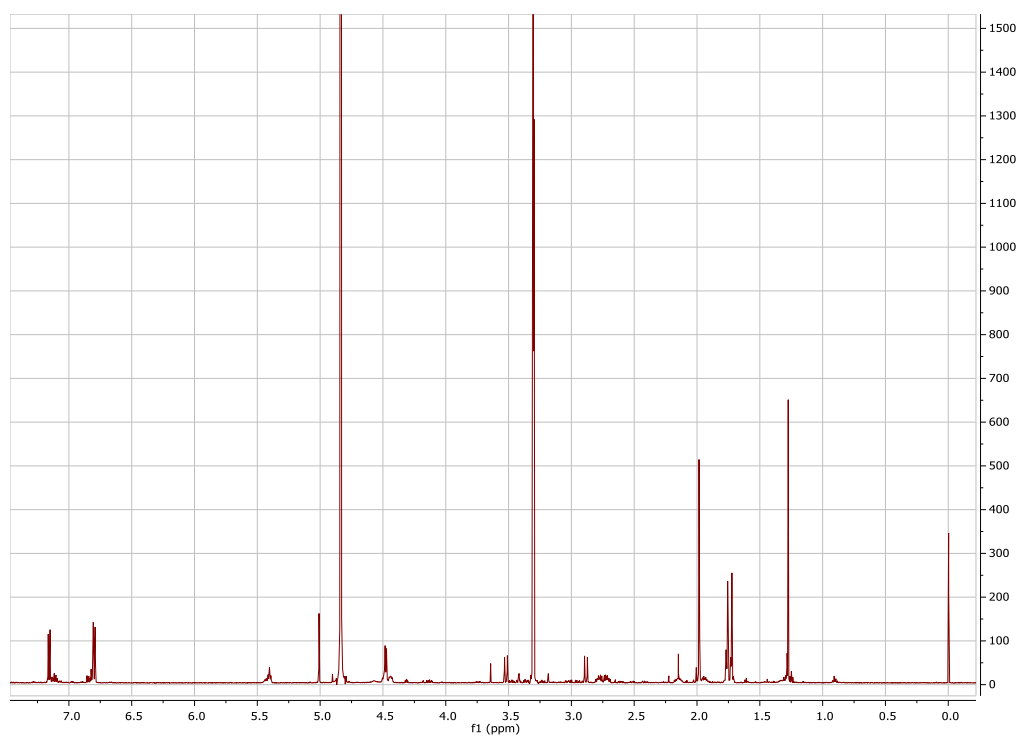

**B**

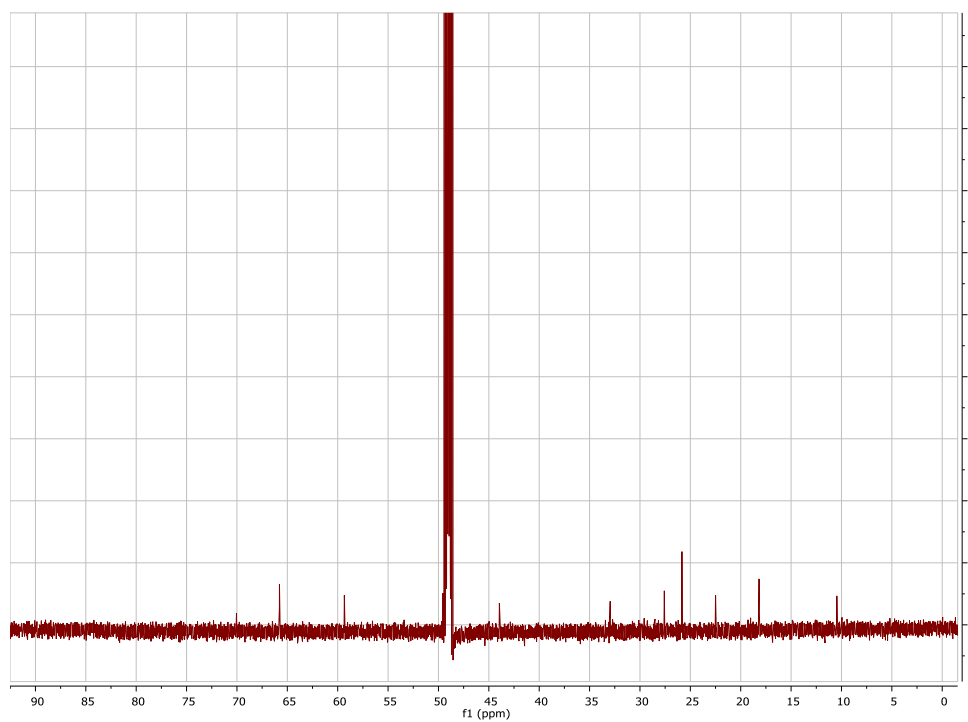

**C**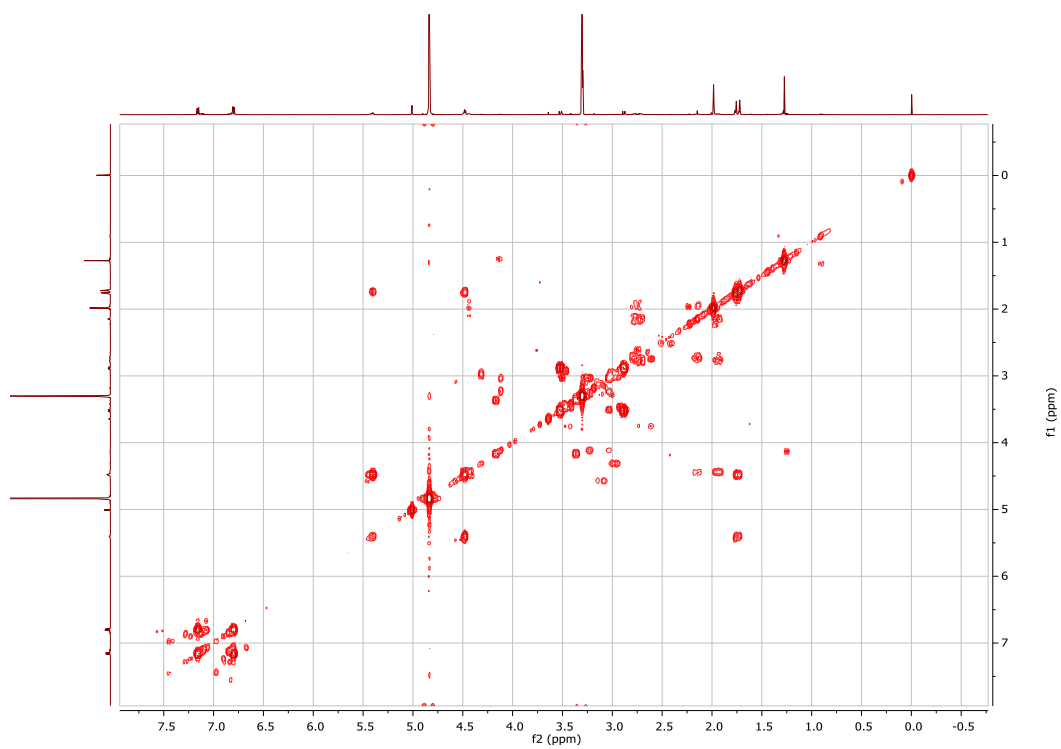**D**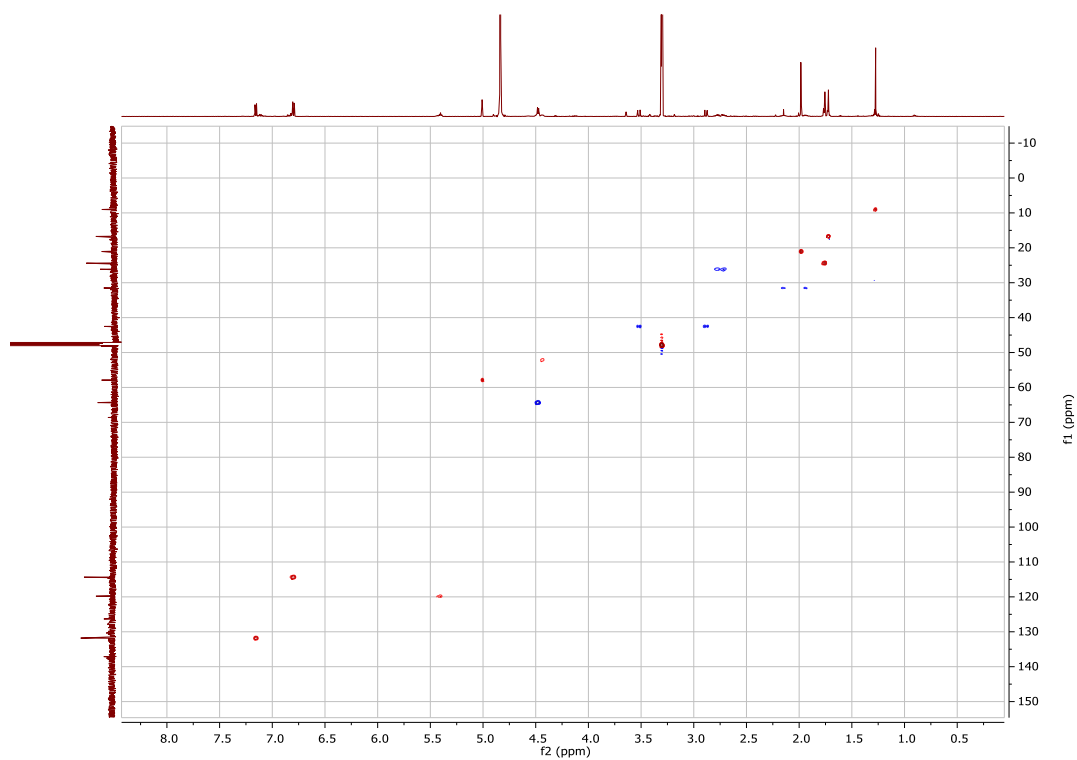

E

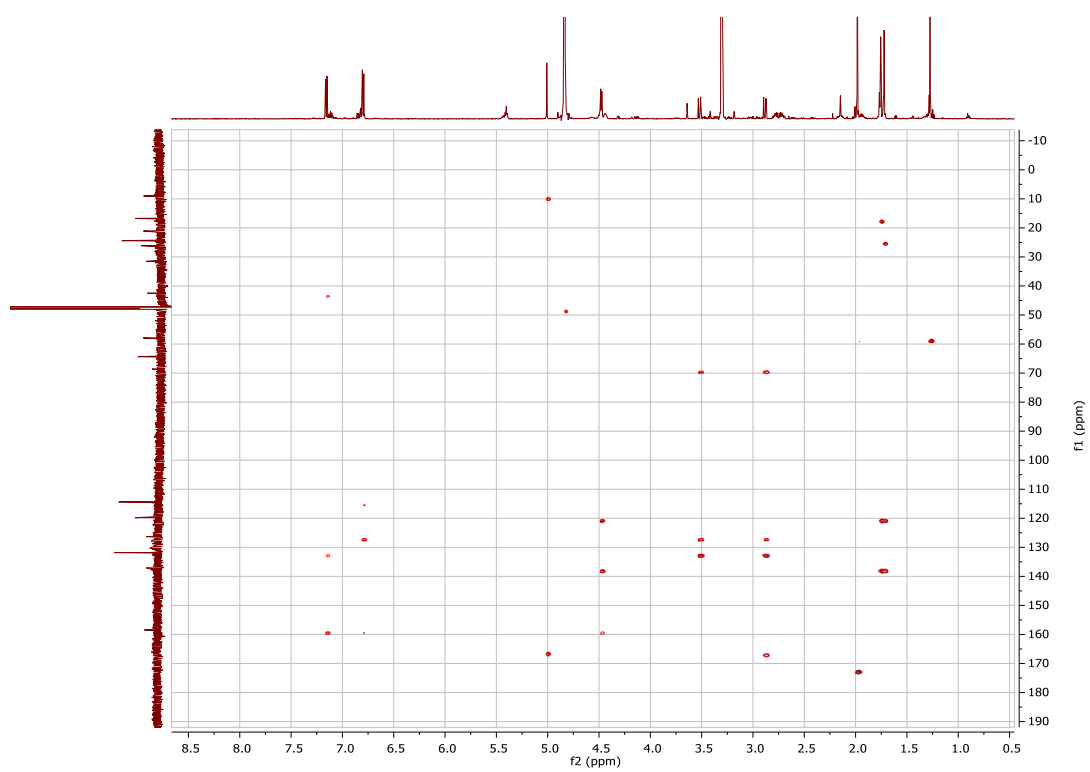

Supplement: S7 Fig — (A) 1H-NMR, (B) 13C-NMR, (C) H,H-COSY, (D) HSQC, (E) HMBC. (PDF) [file pone.0158945.s007.pdf]

**A**

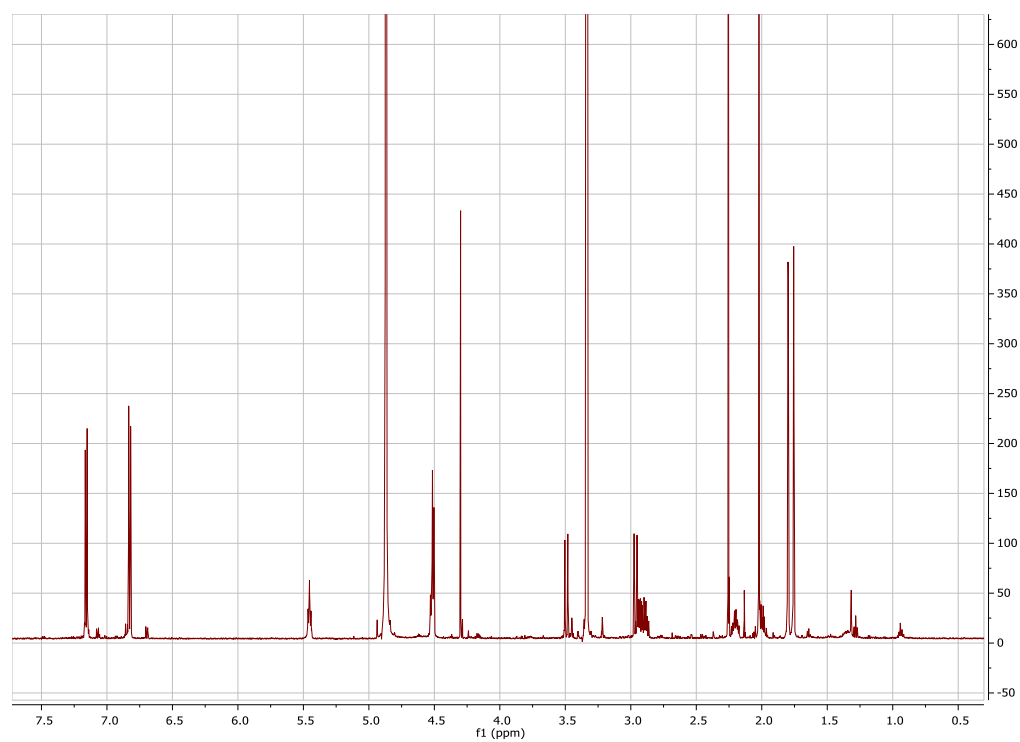

**B**

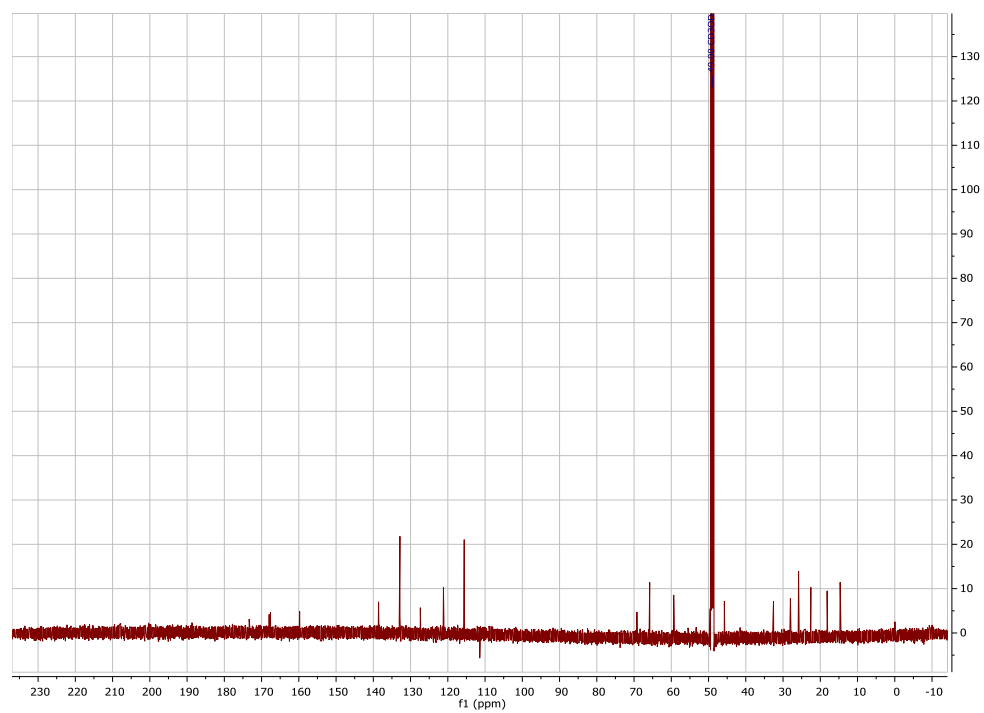

C

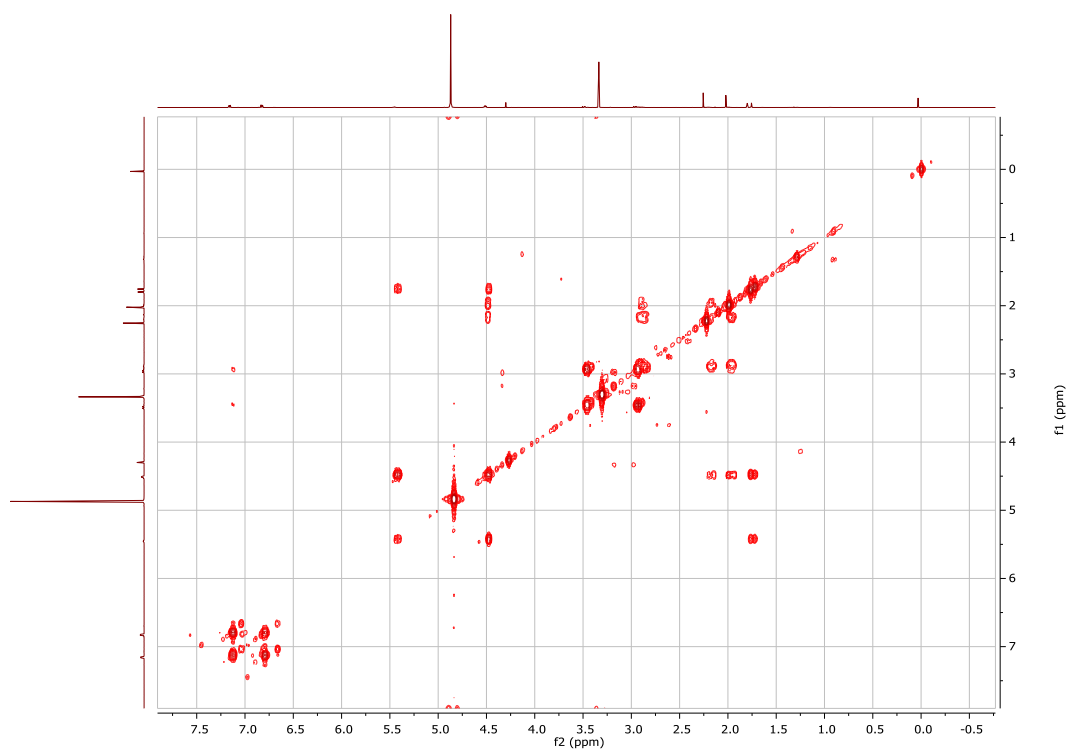

D

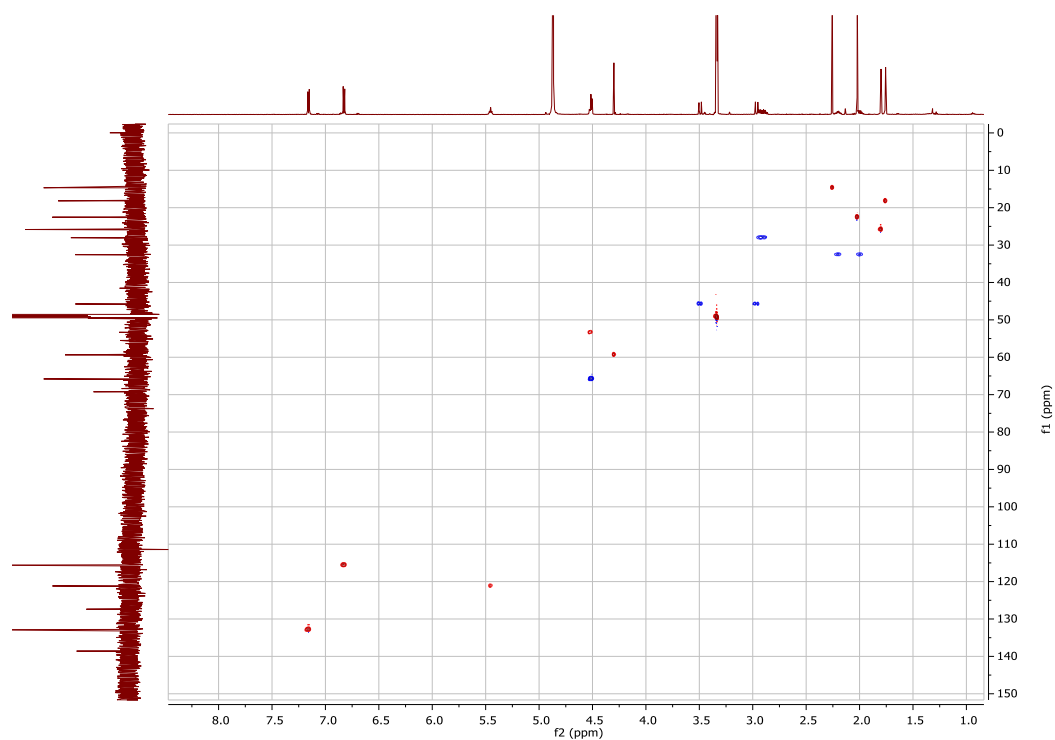

E

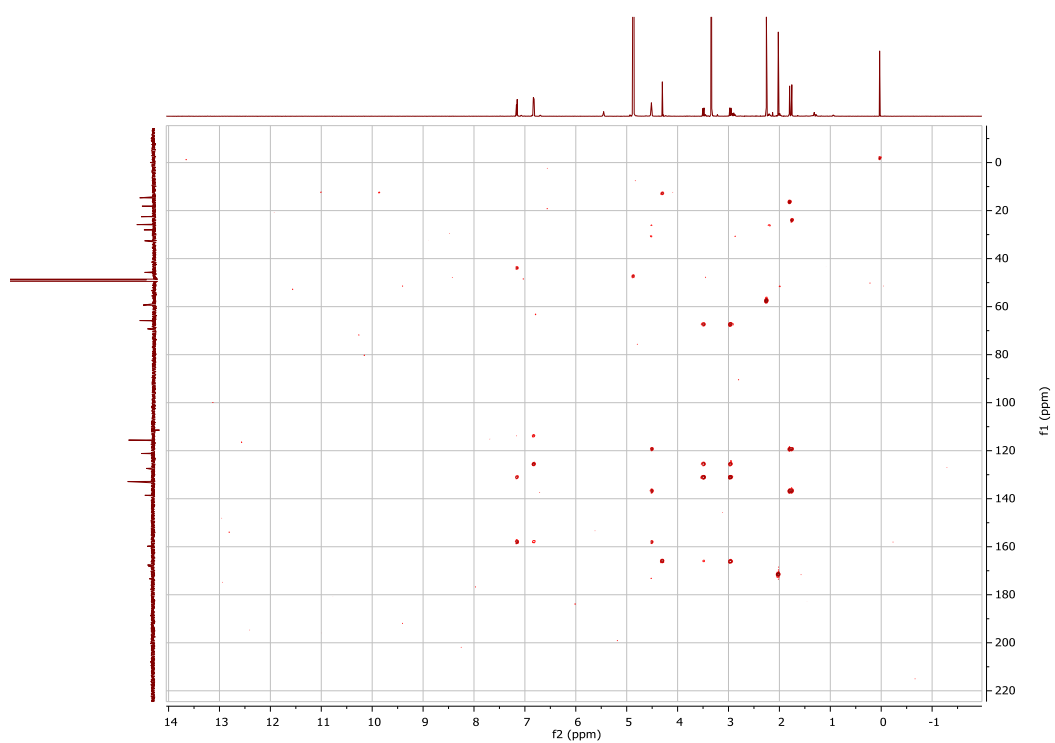

Supplement: S8 Fig — (A) 1H-NMR, (B) 13C-NMR, (C) H,H-COSY, (D) HSQC, (E) HMBC. (PDF) [file pone.0158945.s008.pdf]

**A**

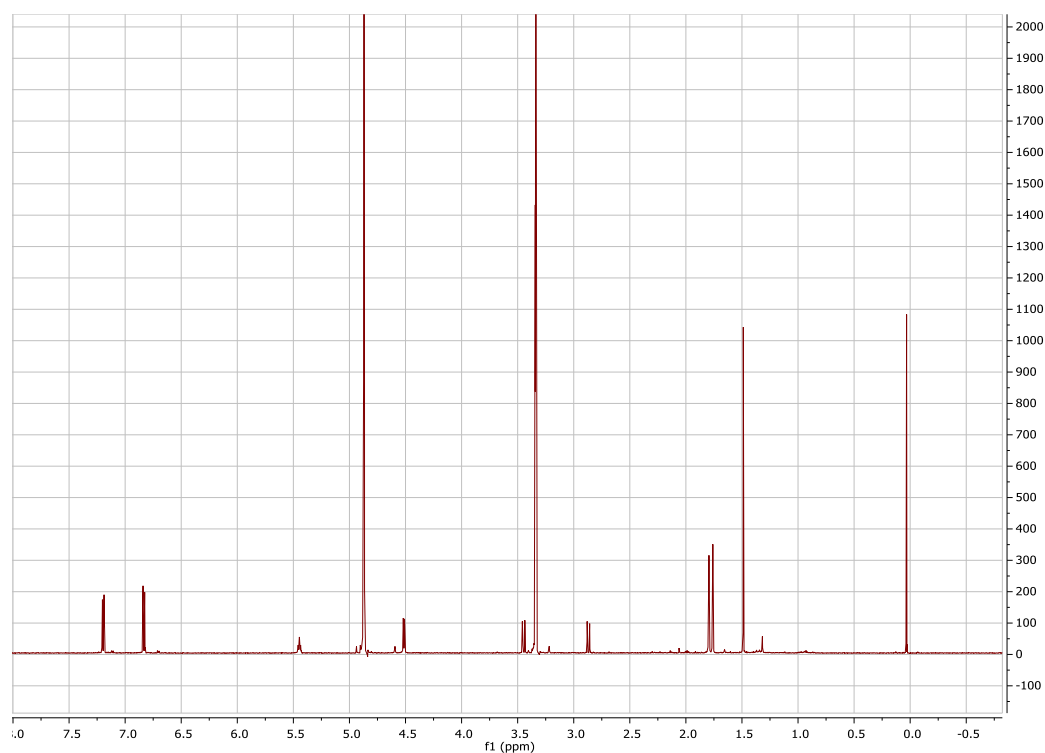

**B**

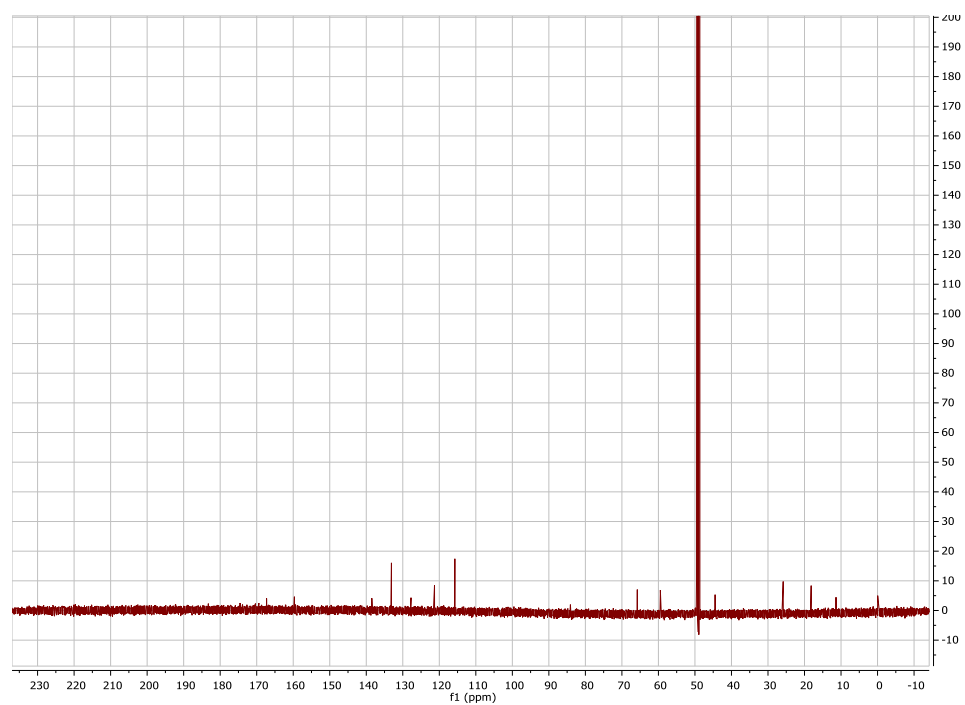

C

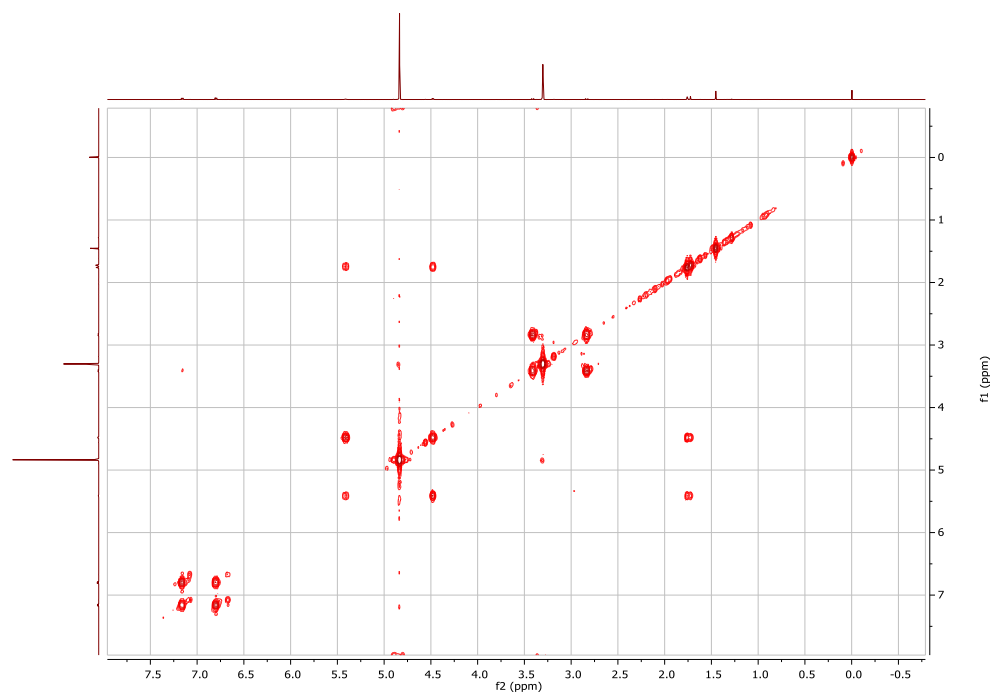

D

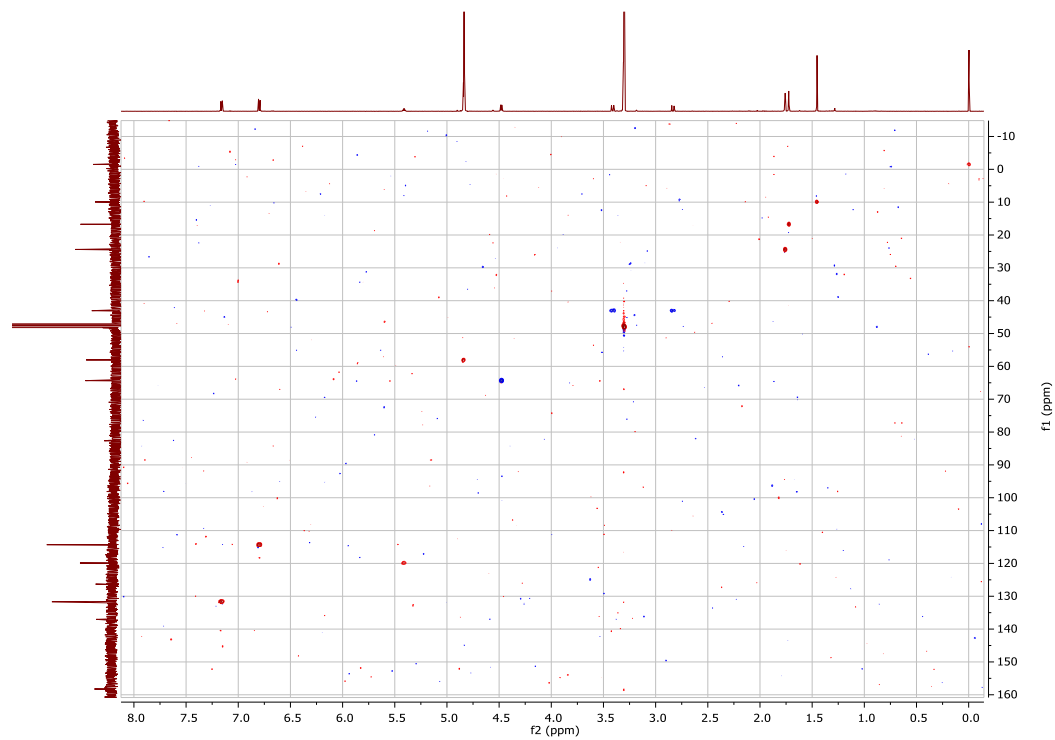

E

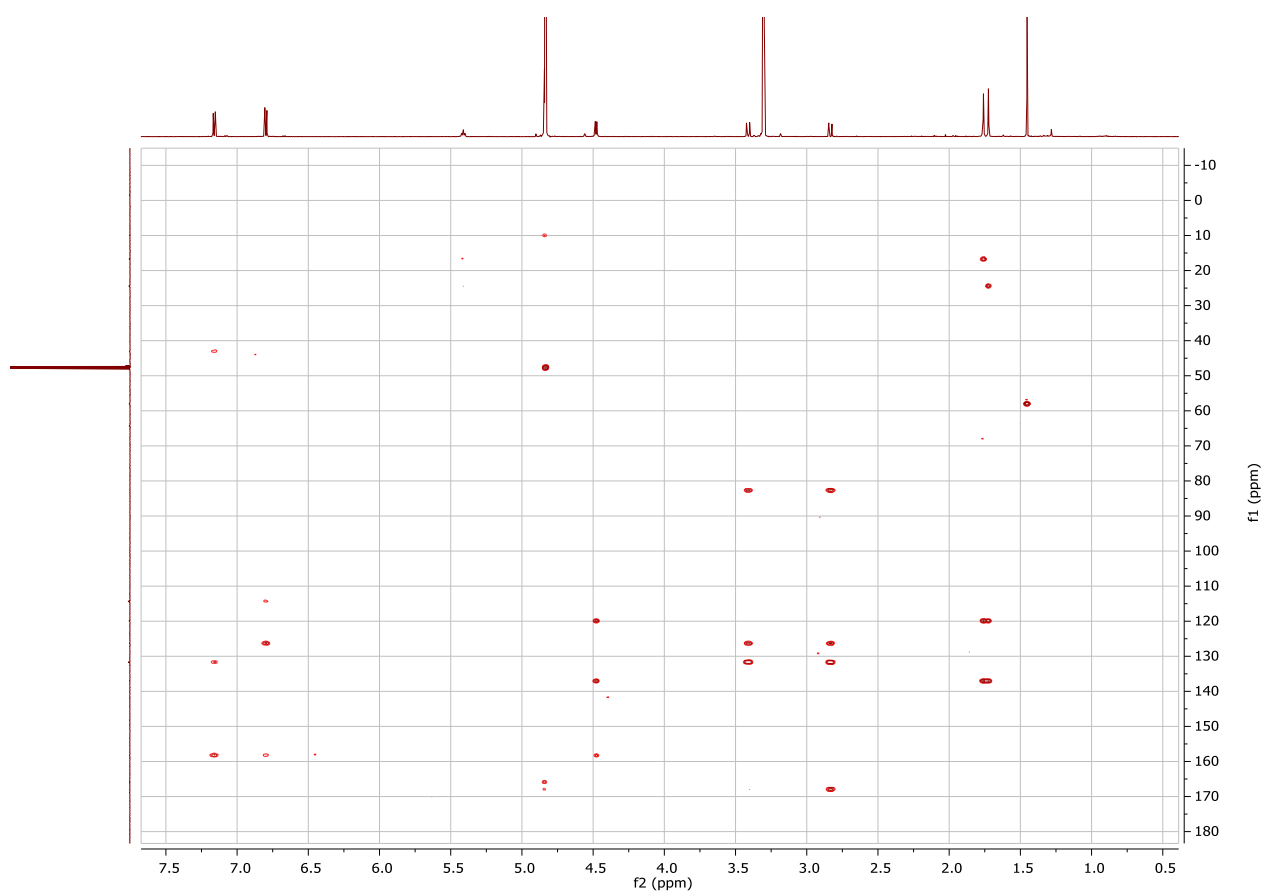

Supplement: S9 Fig — (A) 1H-NMR, (B) 13C-NMR, (C) H,H-COSY, (D) HSQC, (E) HMBC. (PDF) [file pone.0158945.s009.pdf]

**A**

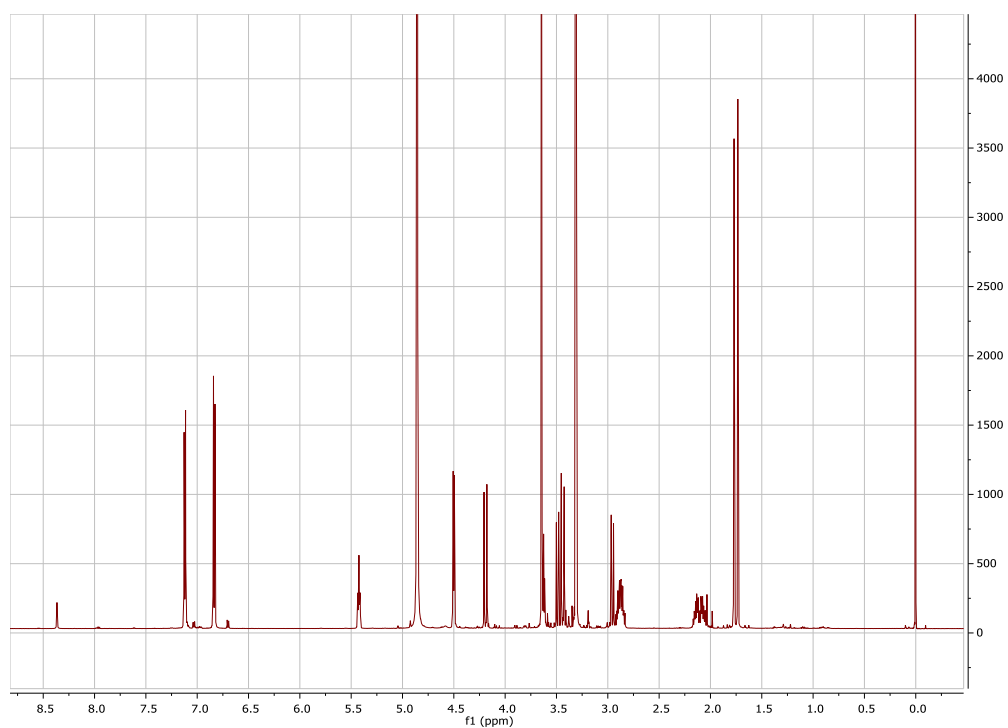

**B**

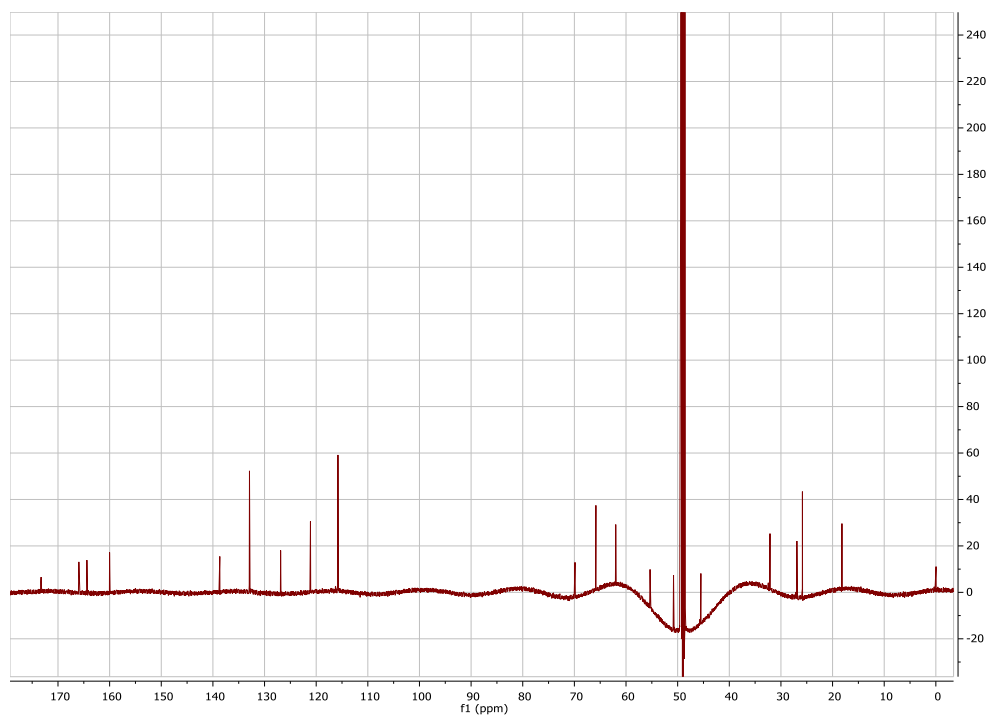

**C**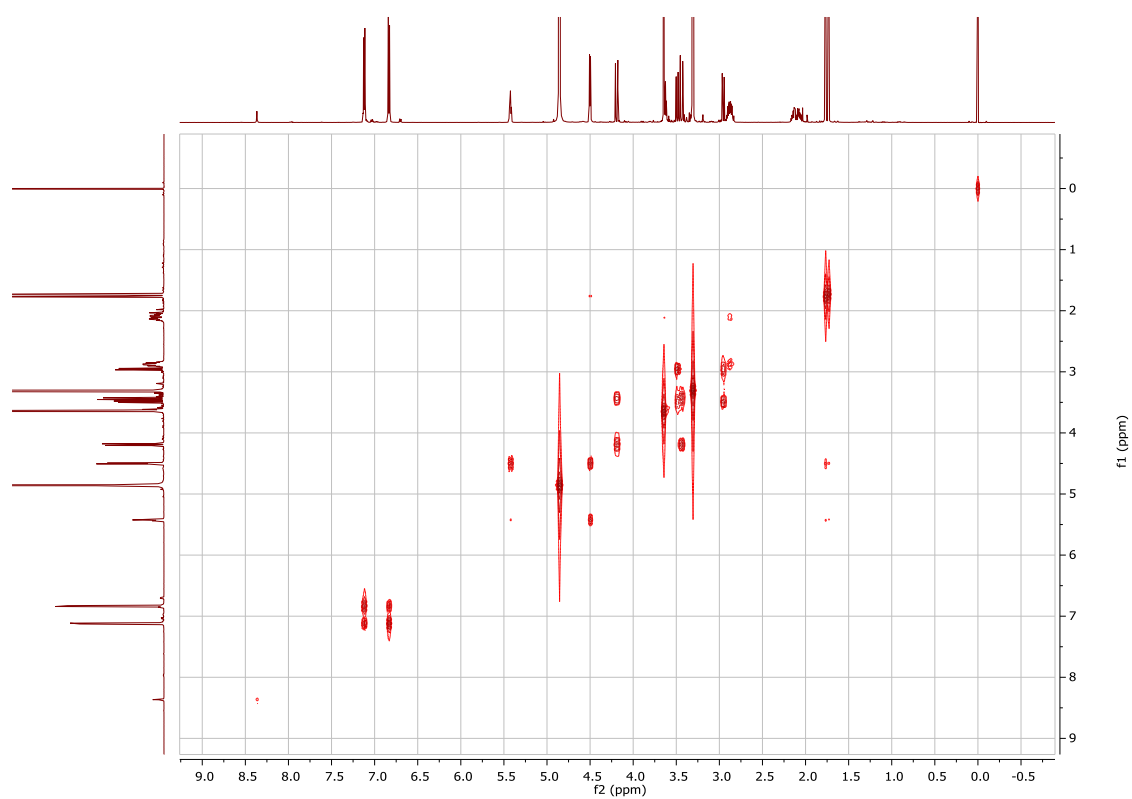**D**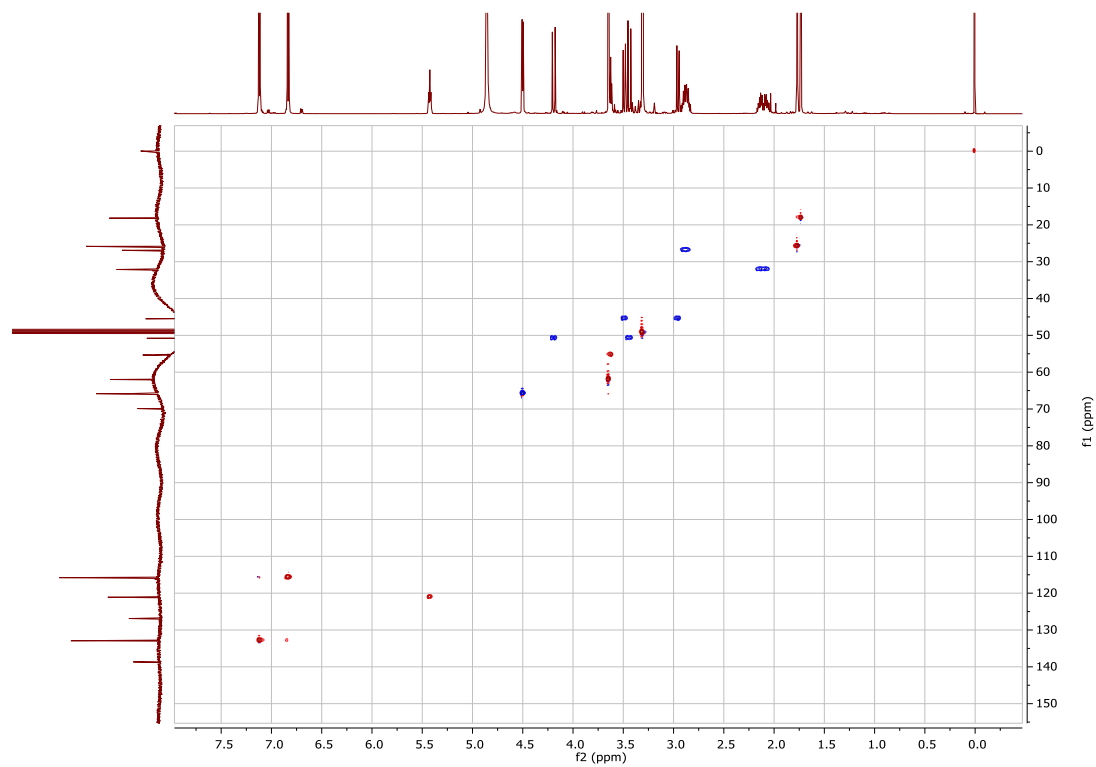

E

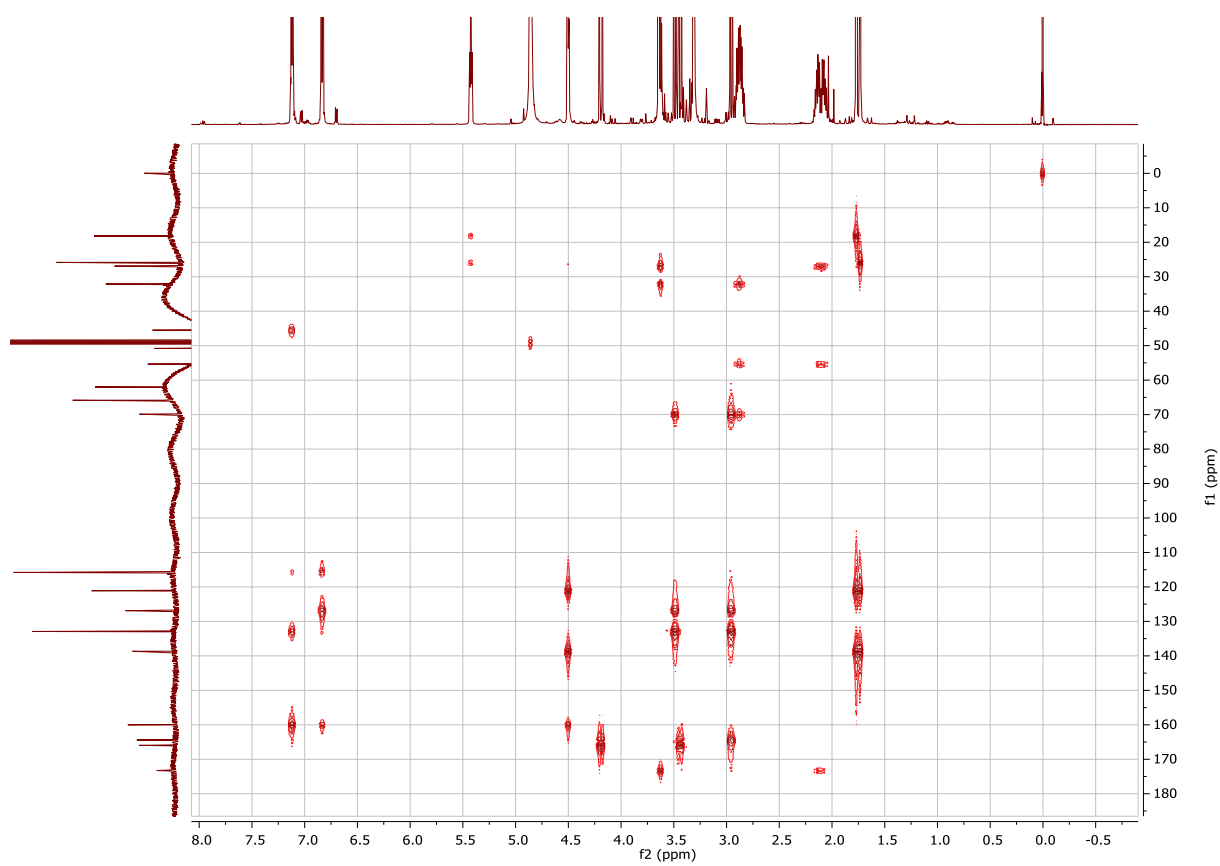

Supplement: S10 Fig — (A) 1H-NMR, (B) 13C-NMR, (C) H,H-COSY, (D) HSQC, (E) HMBC. (PDF) [file pone.0158945.s010.pdf]

**A**

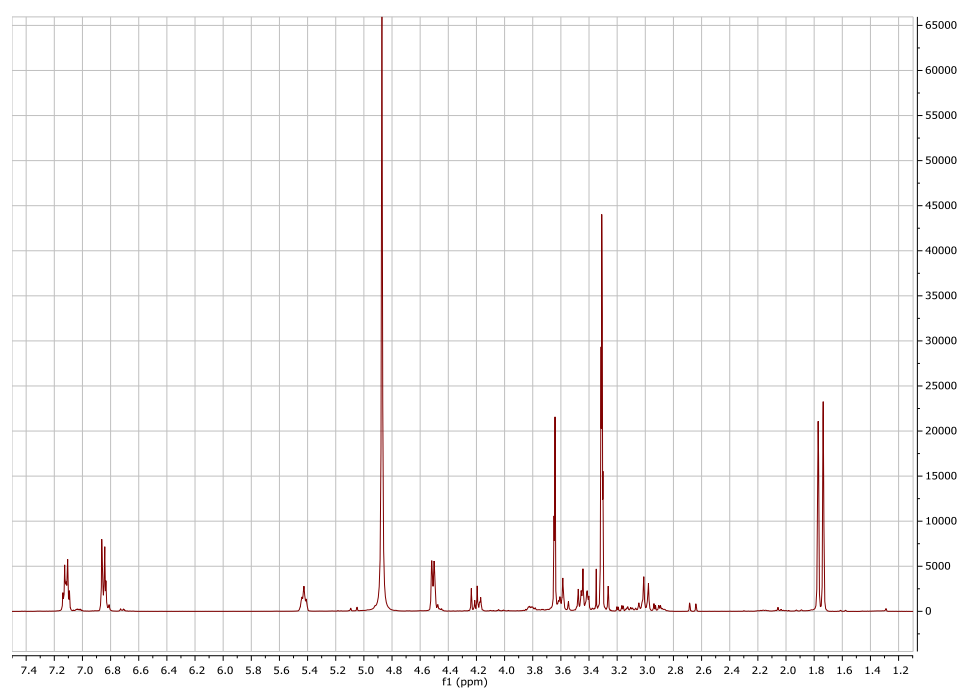

**B**

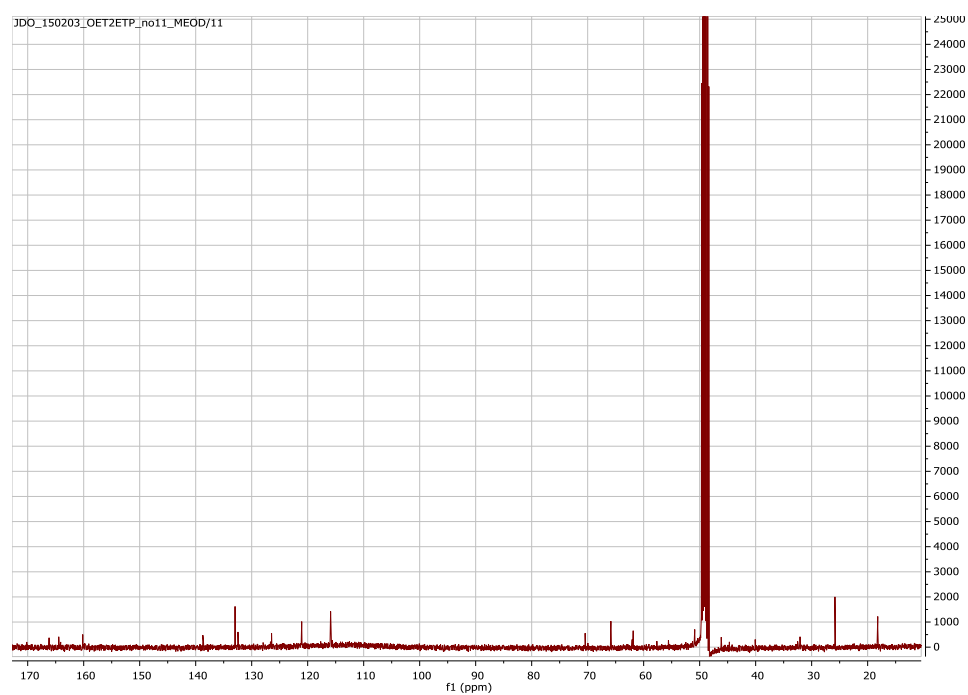

**C**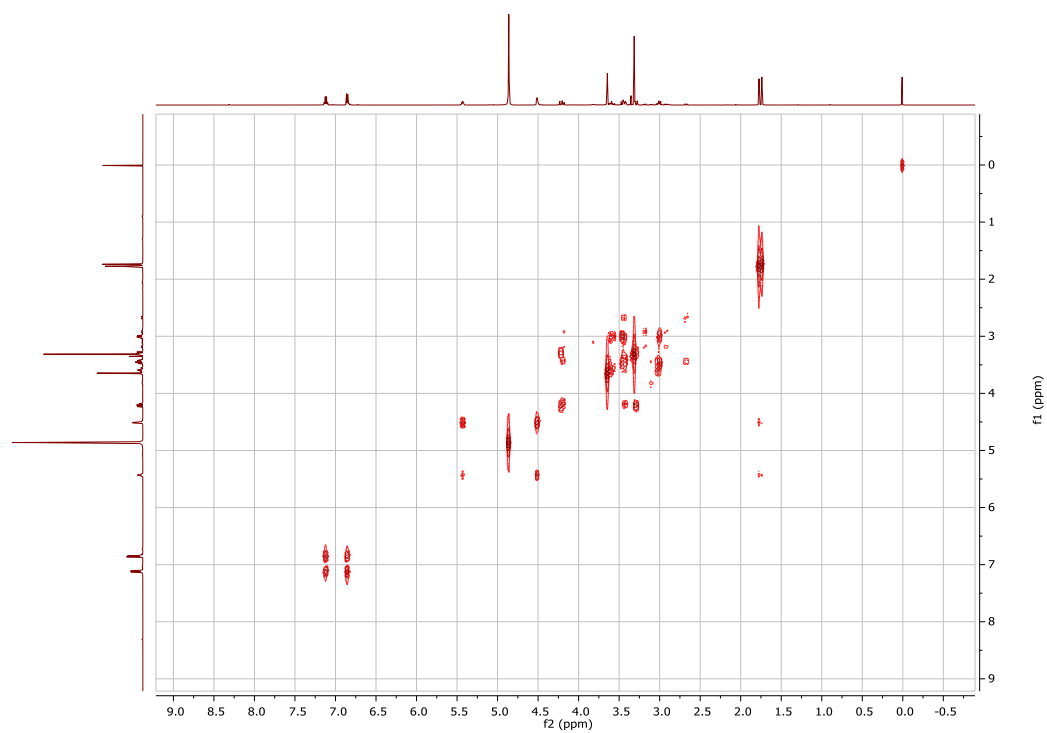**D**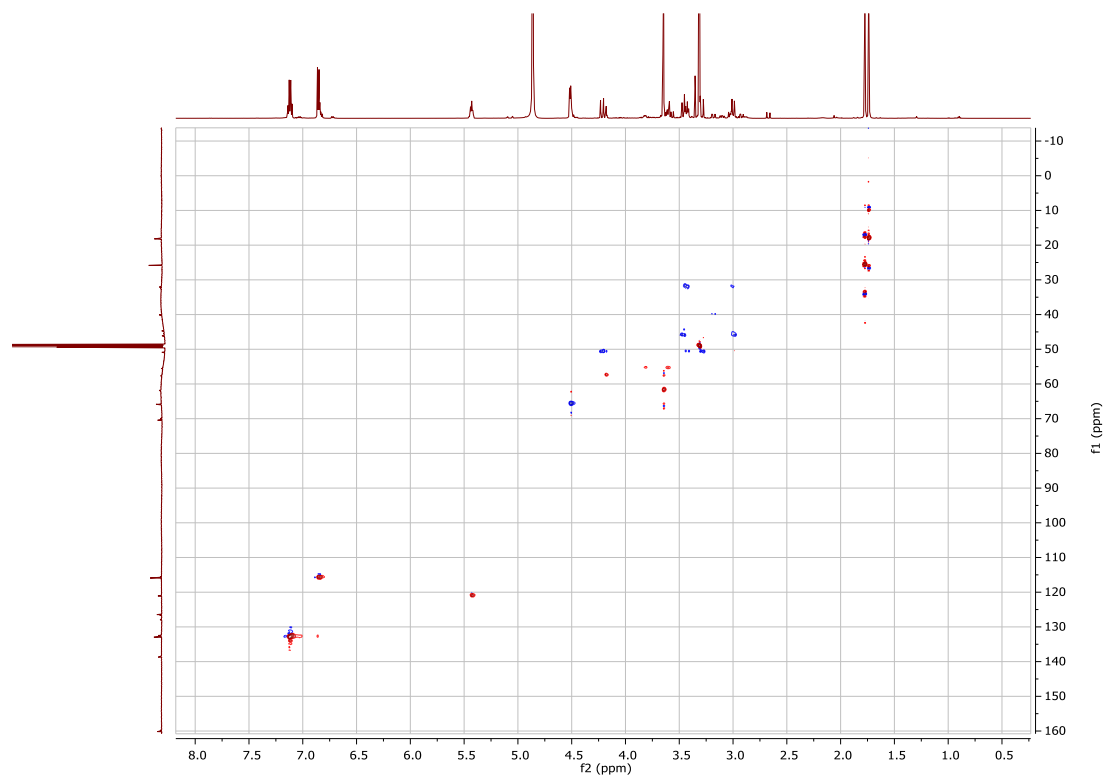

E

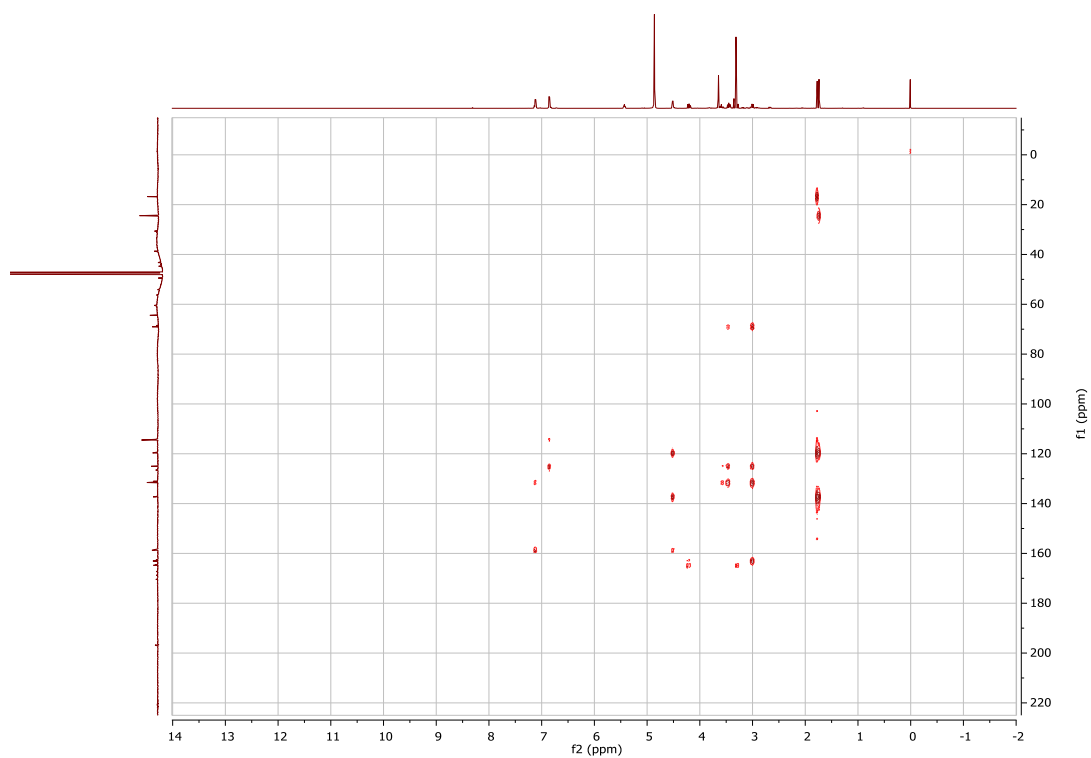

Supplement: S11 Fig — (A) 1H-NMR, (B) 13C-NMR, (C) H,H-COSY, (D) HSQC, (E) HMBC. (PDF) [file pone.0158945.s011.pdf]

**A**

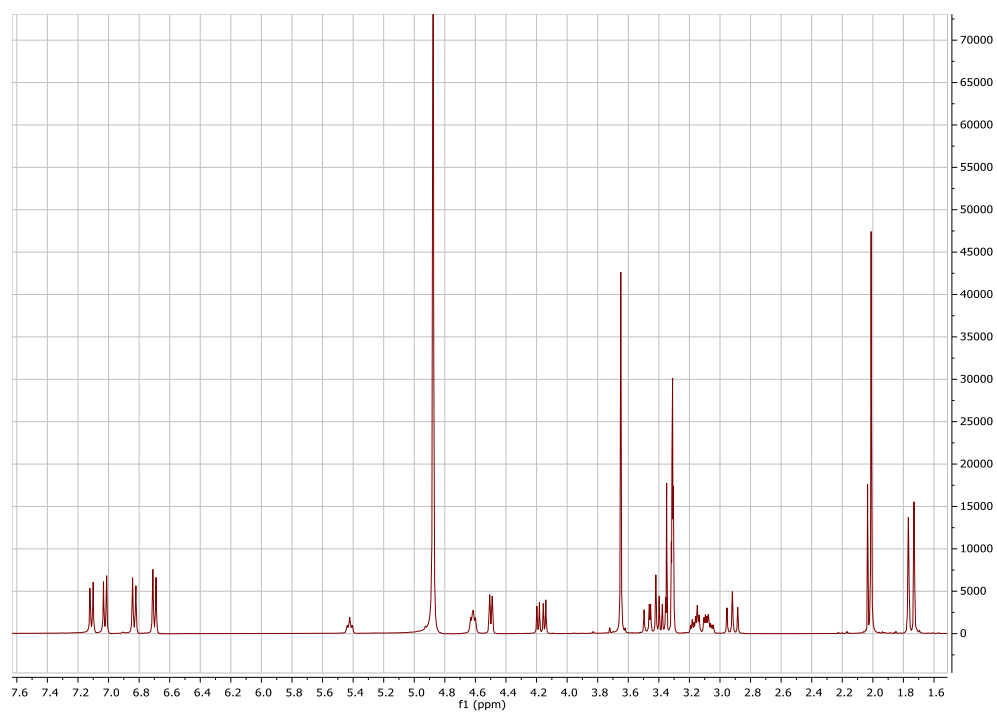

**B**

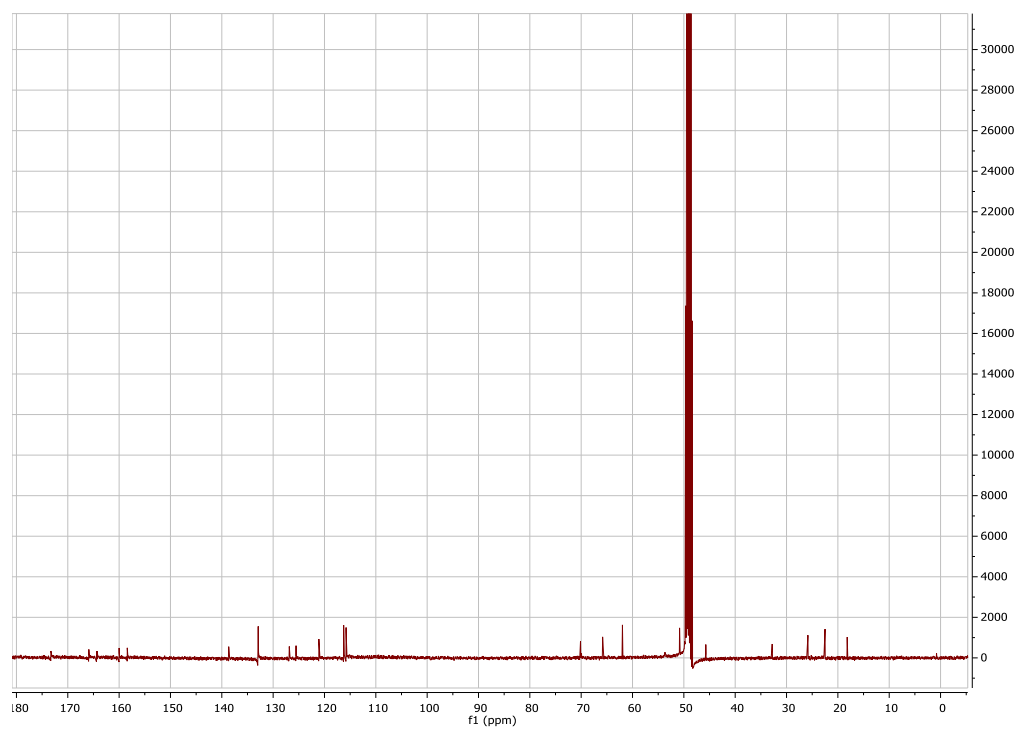

**C**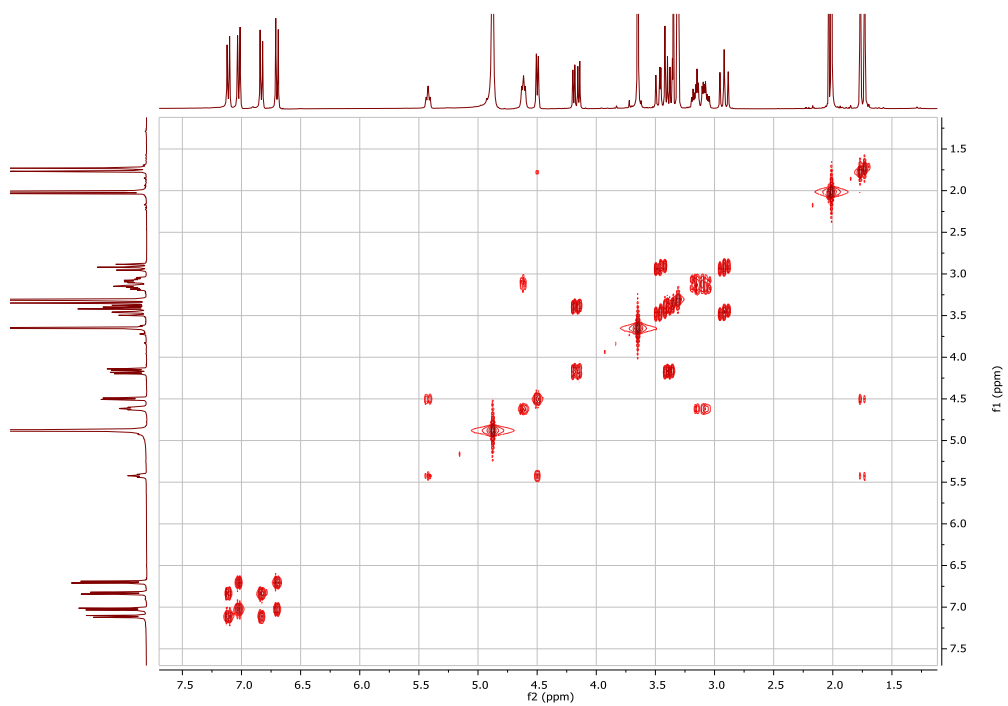**D**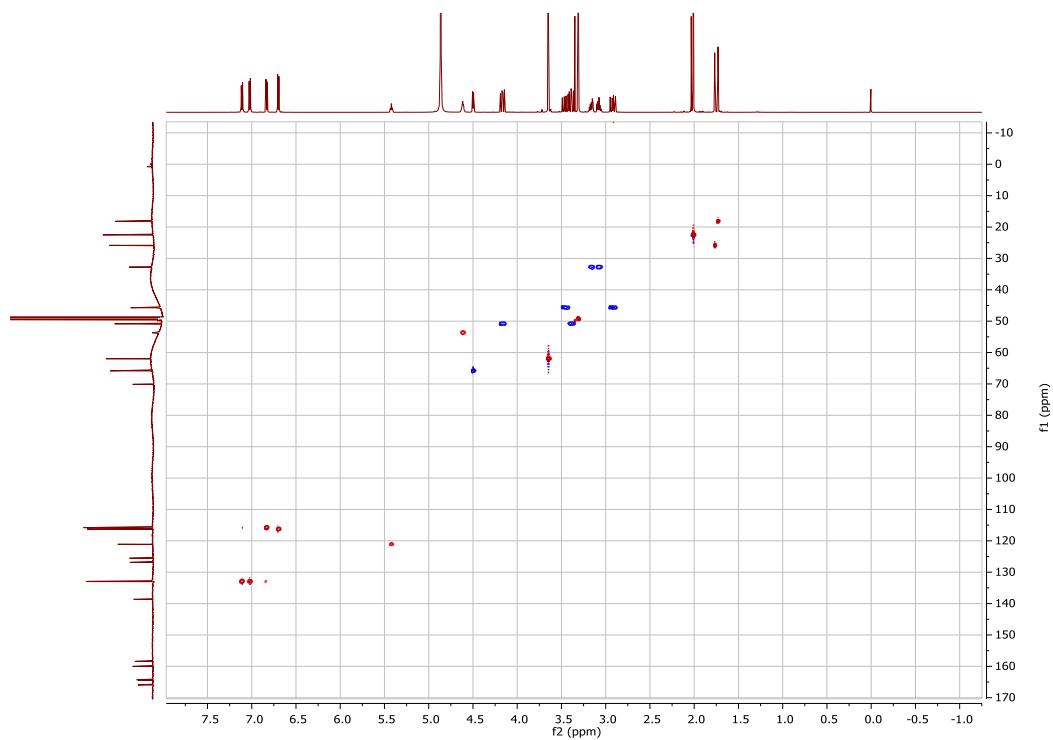

E

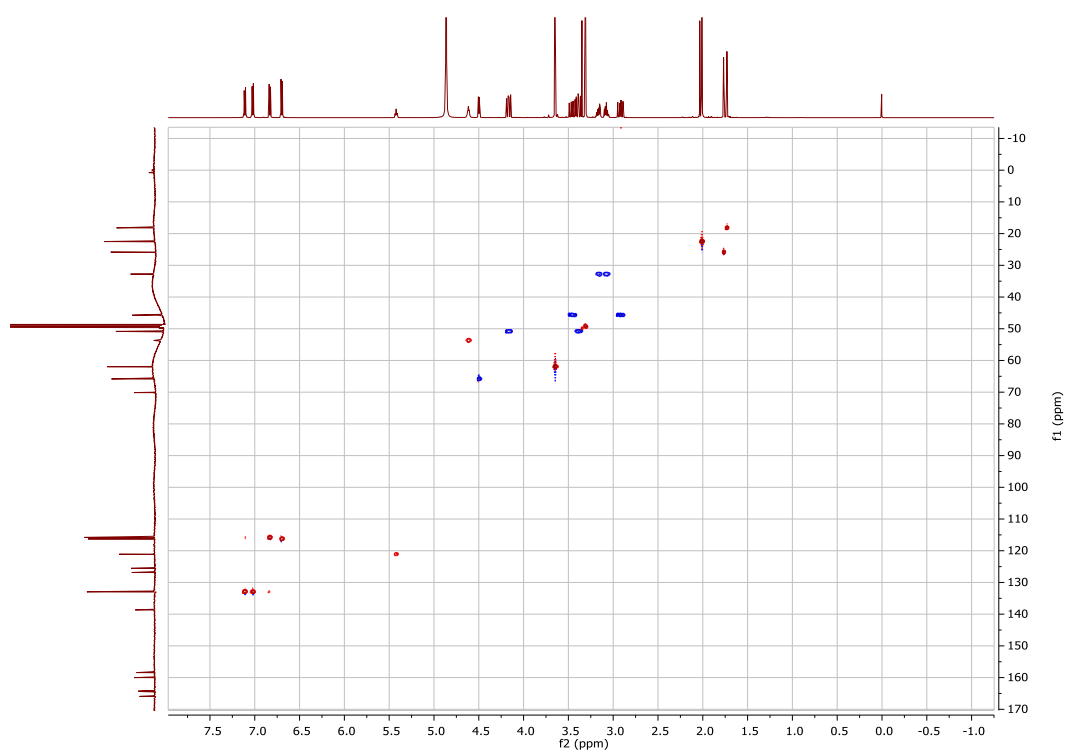

Supplement: S12 Fig — (A) 1H-NMR, (B) 13C-NMR, (C) H,H-COSY, (D) HSQC, (E) HMBC. (PDF) [file pone.0158945.s012.pdf]

**A**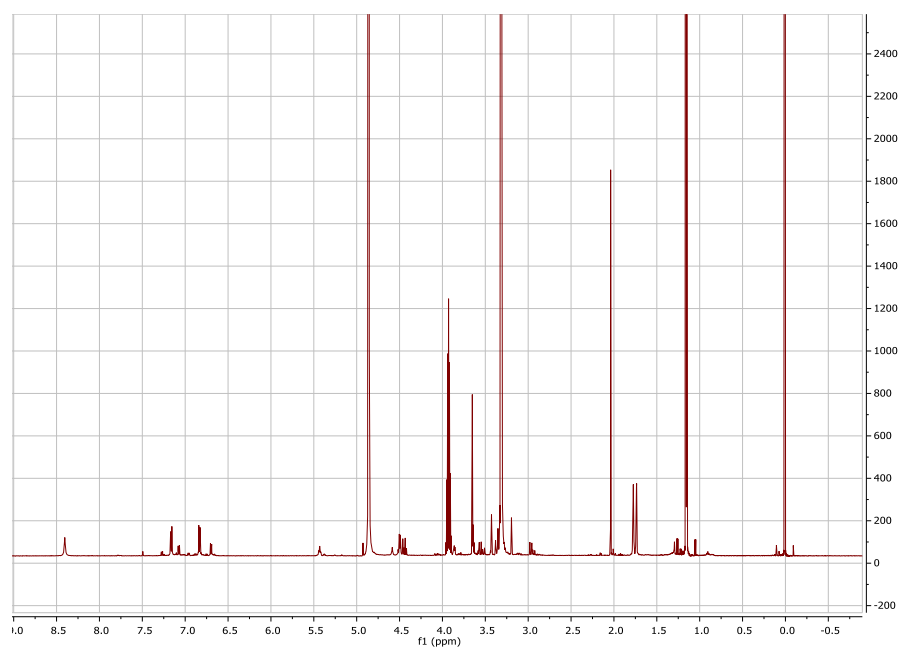**B**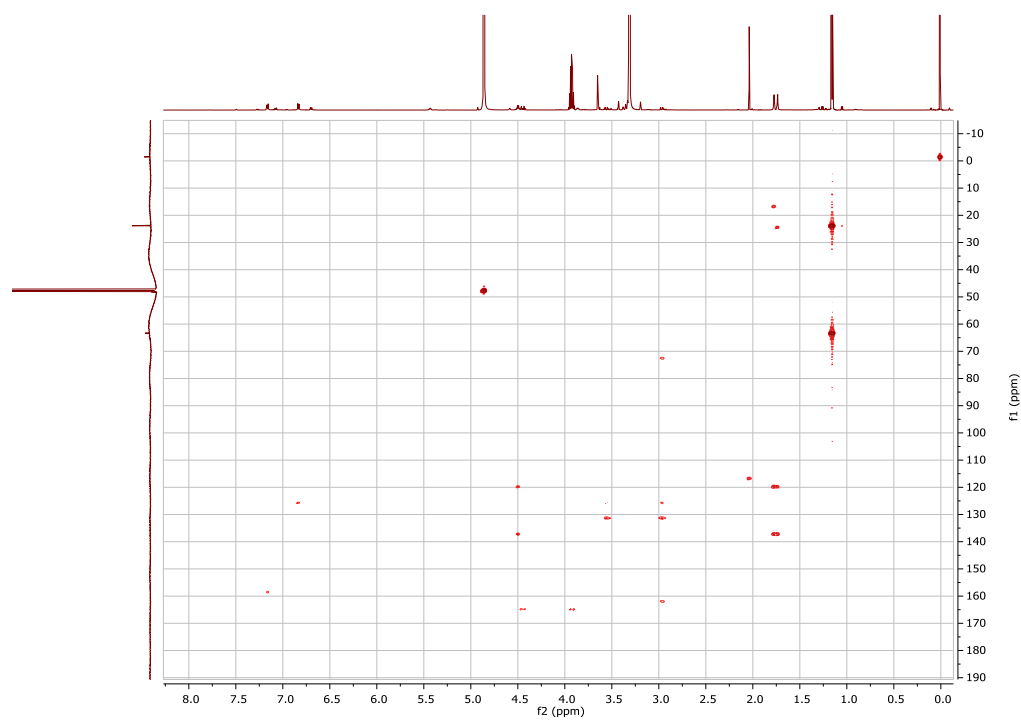

Supplement: S13 Fig — (A) 1H-NMR, (B) HMBC. (PDF) [file pone.0158945.s013.pdf]

**A**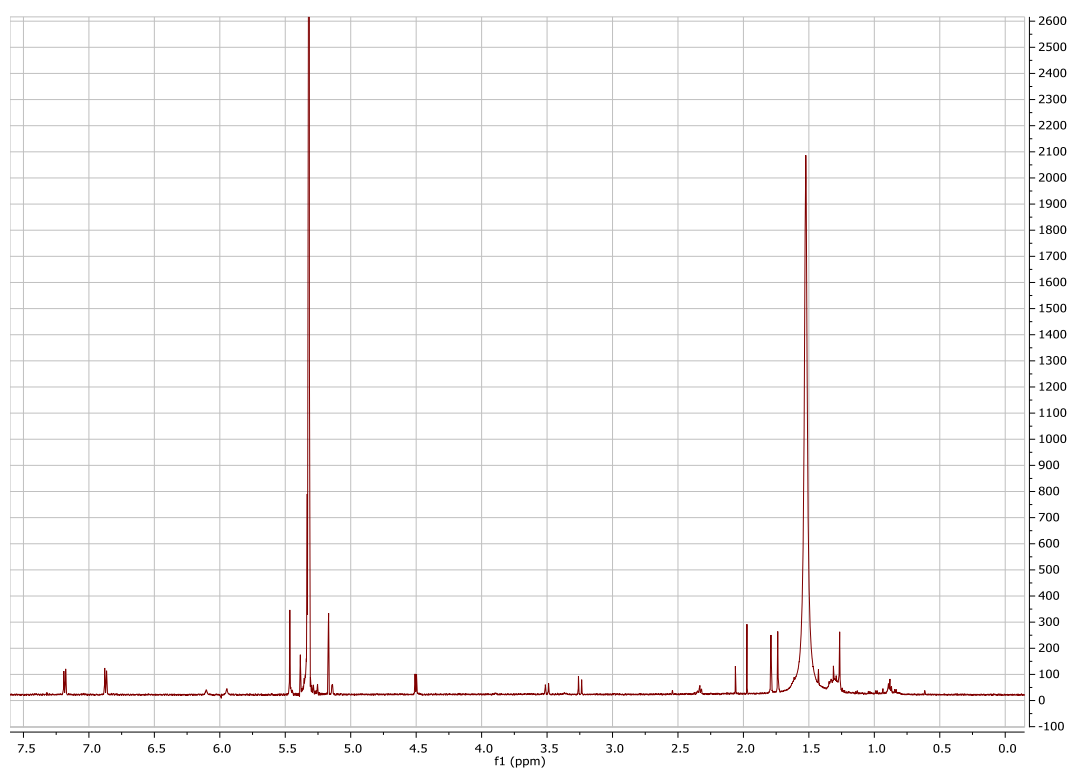**B**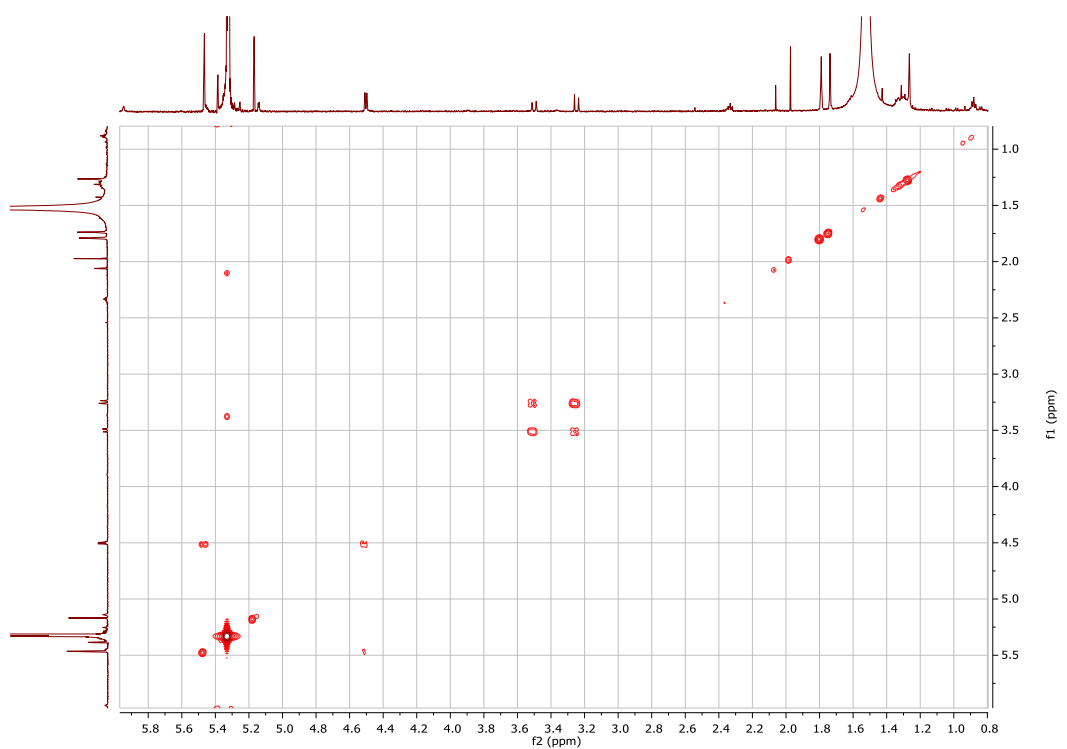

**C**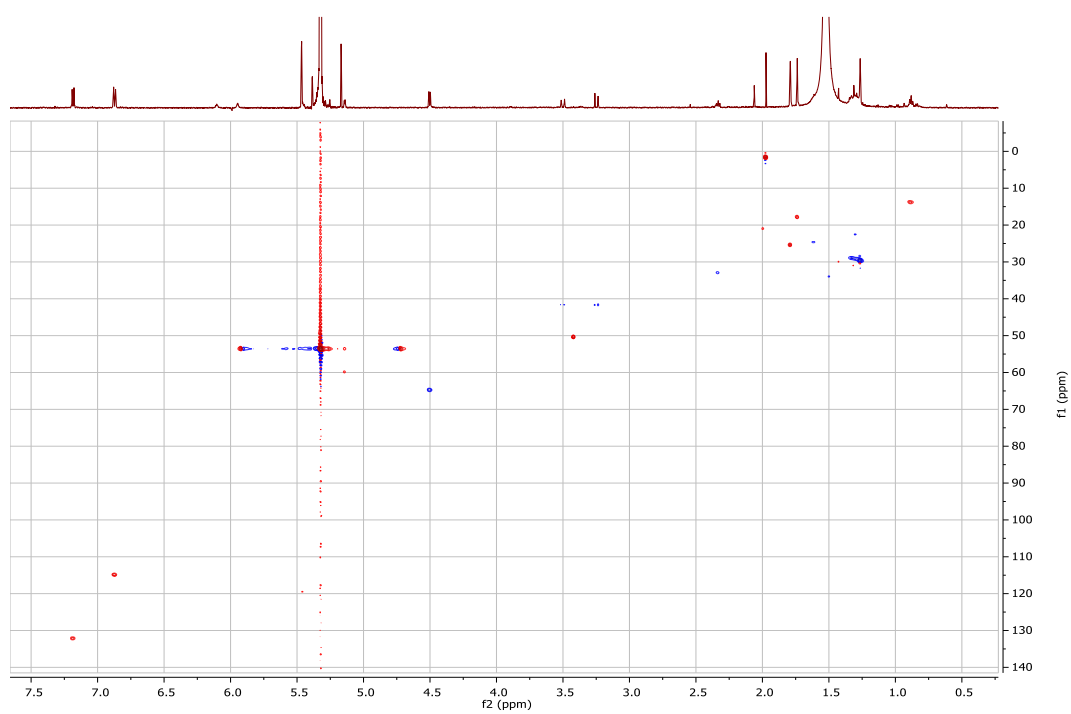**D**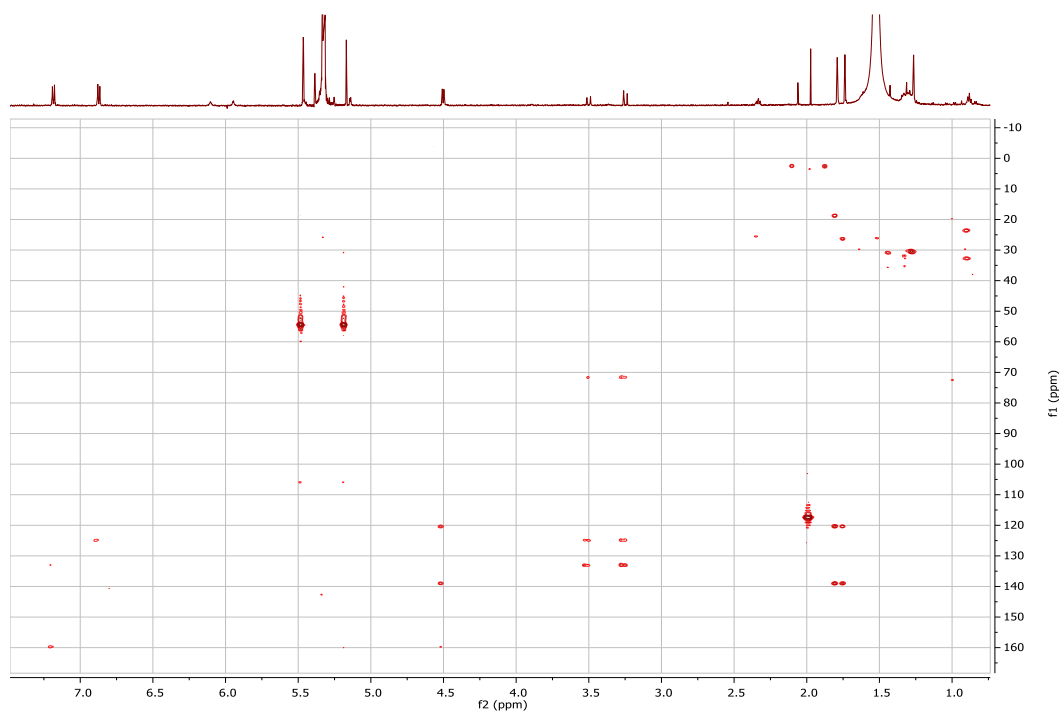

Supplement: S14 Fig — (A) 1H-NMR, (B), H-H COESY, (C) HSQC, (D) HMBC. (PDF) [file pone.0158945.s014.pdf]
