# Supplementary material for: The Epipolythiodiketopiperazine Gene Cluster in Claviceps purpurea: Dysfunctional Cytochrome P450 Enzyme Prevents Formation of the Previously Unknown Clapurines
Source: PLoS One. 2016 Jul 8;11(7):e0158945. doi: 10.1371/journal.pone.0158945 (PMC4938161; doi:10.1371/journal.pone.0158945)

HRMS^n analysis of compound **2a/b**

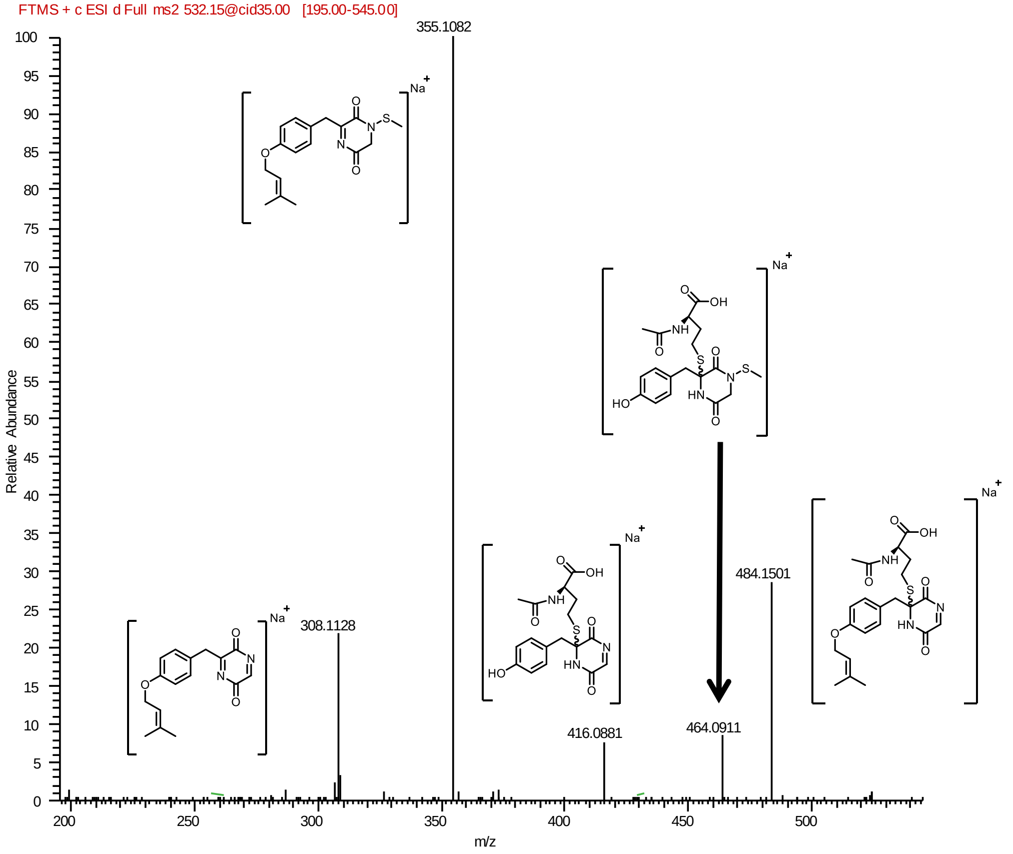

HRMS^n analysis of compound **3**

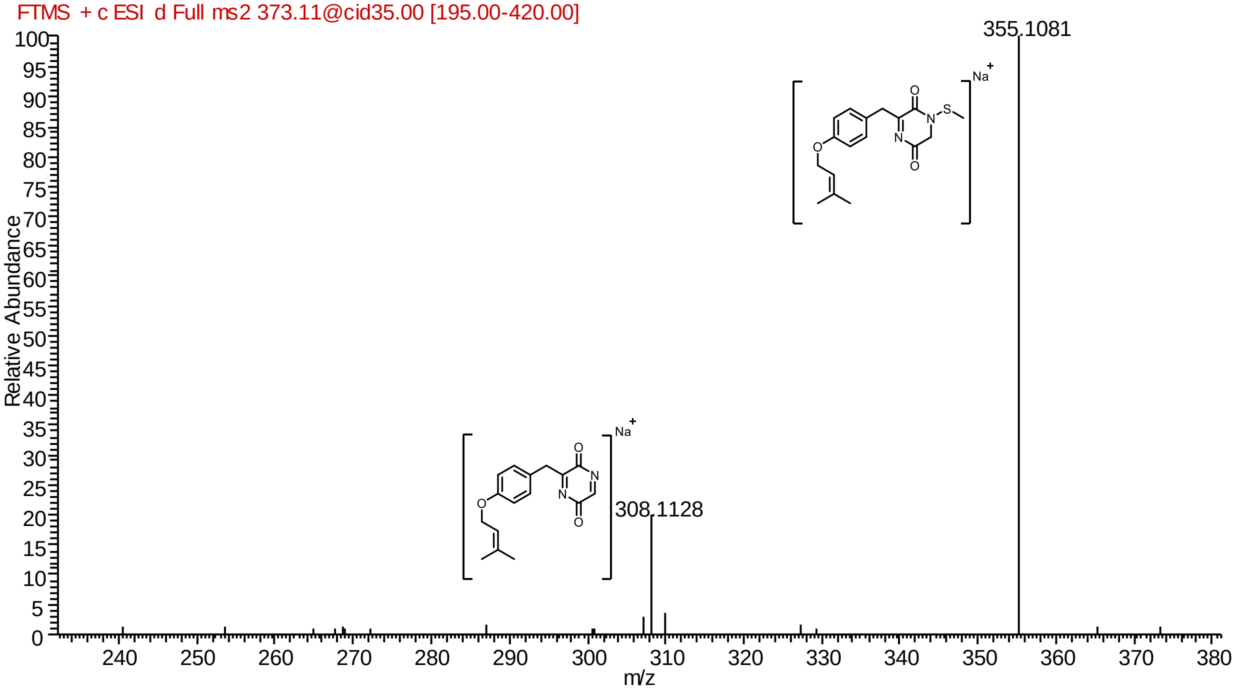

HRMS^n analysis of compound **5**

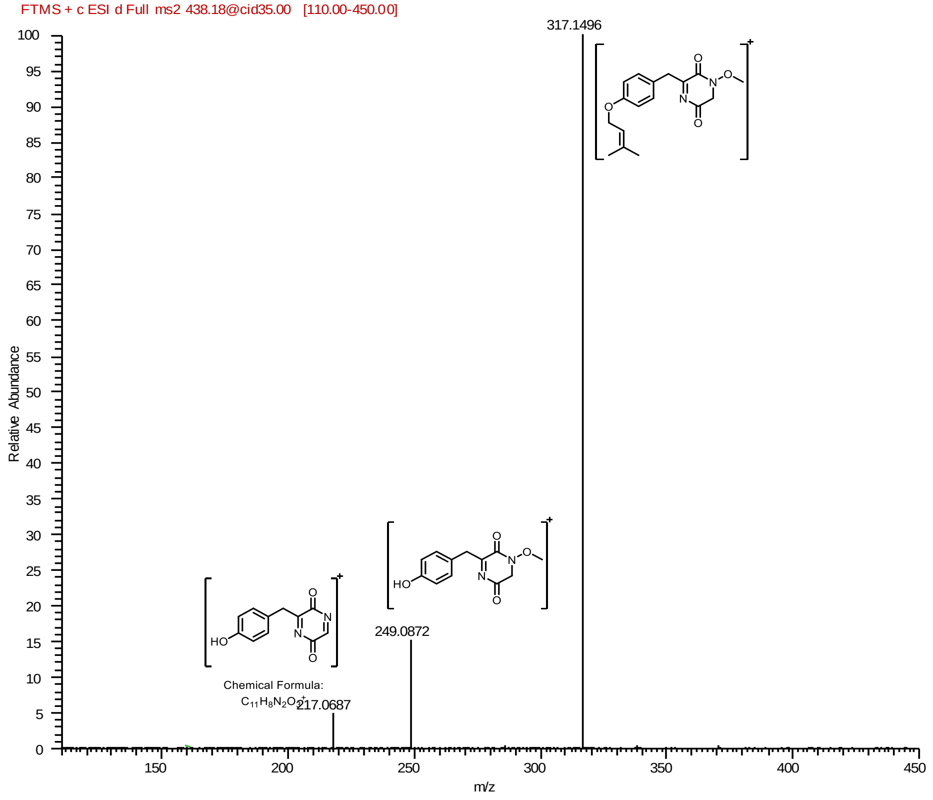

HRMS^n analysis of compound **6**

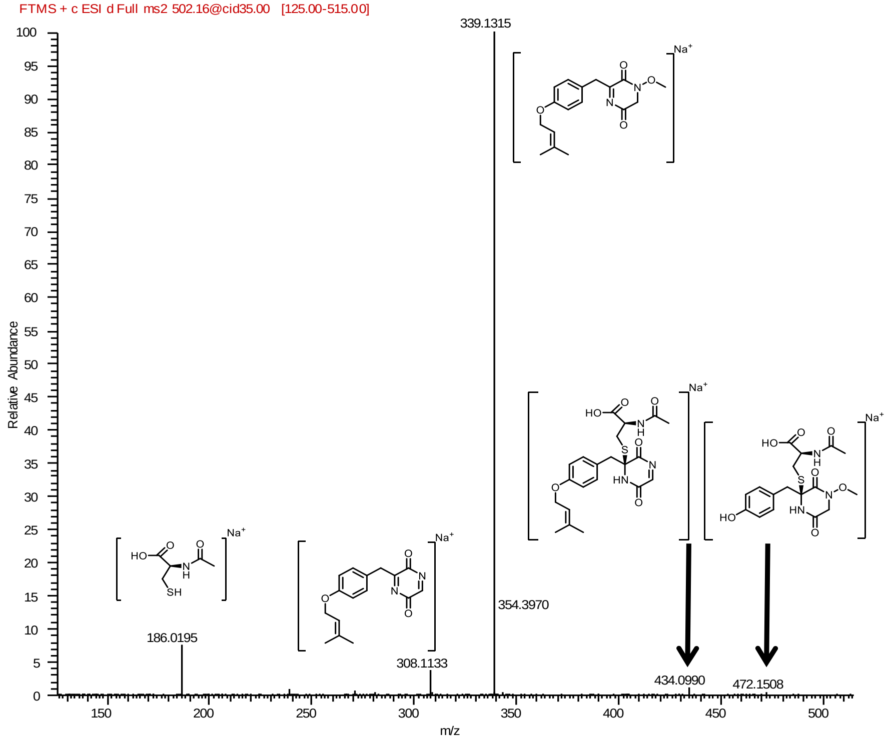

HRMS^n analysis of compound **7**

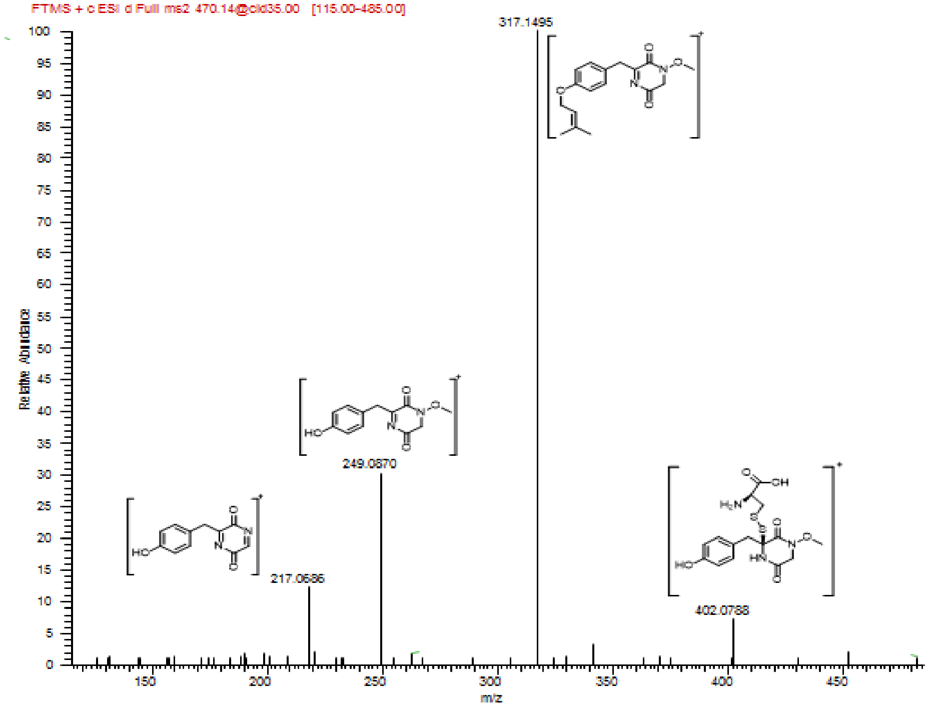

HRMS^n analysis of compound **8**

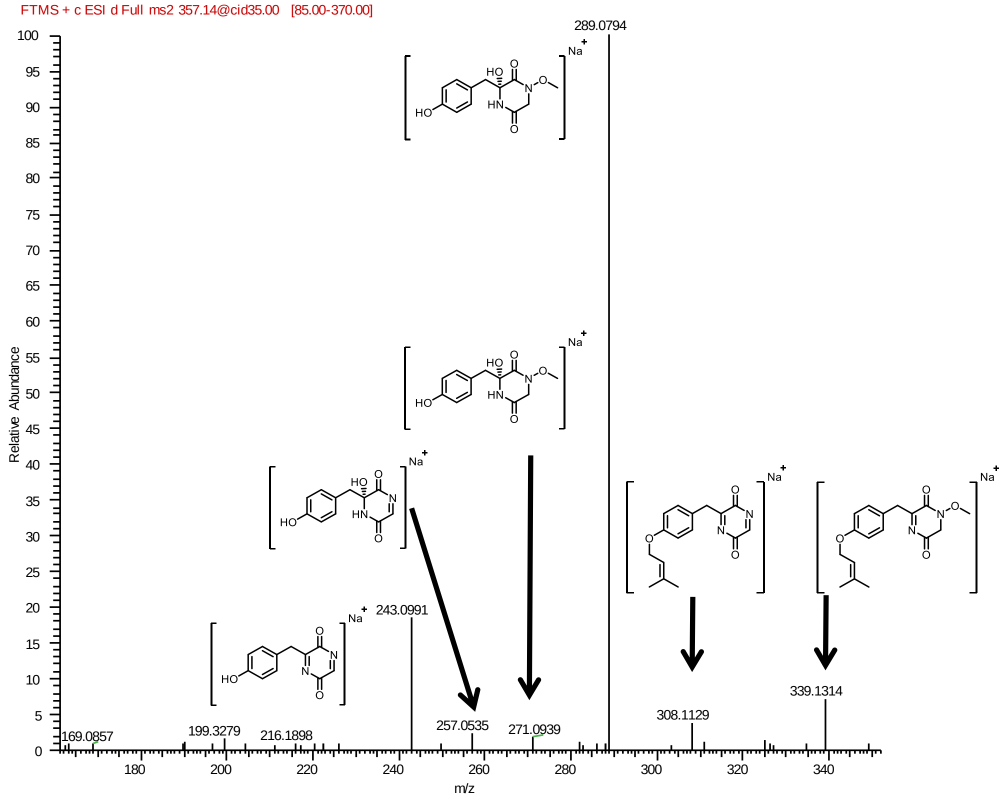

Supplement: S15 Fig — (PDF) [file pone.0158945.s015.pdf]
