# Supplementary material for: The Epipolythiodiketopiperazine Gene Cluster in Claviceps purpurea: Dysfunctional Cytochrome P450 Enzyme Prevents Formation of the Previously Unknown Clapurines
Source: PLoS One. 2016 Jul 8;11(7):e0158945. doi: 10.1371/journal.pone.0158945 (PMC4938161; doi:10.1371/journal.pone.0158945)
Supplement: S1 Table — (PDF) [file pone.0158945.s016.pdf]

Name	Sequence (5'-3')
**Overexpression vector construction**	
OE_Cp_2671_F	CACATCACAATCGATCCAACCATGATGAATCCGATGTTGACCAAC
OE_Cp_2671_R	CATCTTATCTACATACGCTAAGCCTACGGAAGGGTCGCAGTACCC
OE_Cp2672_F	TCACATCACAATCGATCCAACCATGGAGTCCGTCGTAGACGGAAG
OE_Cp2672_R	ATACATCTTATCTACATACGCTATCAGGTGCGCCGCCTGCCATACG
OE_sirC_R	ATACATCTTATCTACATACGCTAGGAGCCTCCTGACGGAACC
OE_sirC_F	TCACATCACAATCGATCCAAccATGGAGTCTATTGTCTACGAAACG
**Knockout vector construction**	
2681_5F	GCCAGGGTTTTCCCAGTCACGACGAAGCTTAGGTCTTCAGAATATTGAGG
2681_5R	ATCCACTTAACGTTACTGAAATCGATAAGGACCTGATTGCCGAG
2681_3F	CTCCTTCAATATCATCTTCTGTCAGTGTCTTCACCGTTCCAGC
2681_3R	AACAATTTCACACAGGAAACAGC AAGCTTCTTATGAGGTCGTGCACCG
CpBle1F	CGGAGACAGAAGATGATATTGAAGGAGCGATCGAGACCTAATACAGCCCC
CpBle1R	GTTGGAGATTTCAGTAACGTTAAGTGGGCATTGCAGATGAGCTGTATCTG
**Diagnostic PCRs**	
2681_diagn_5F	GACAAGGACATGTCTGGAAGG
2681_diagn_3R	TTCGAGATGGCCAAAGCCTC
Phleohi3F2	GTGTTCAGGATCTCGATAAGATACG
Phleo-hi3F	GGCTCAAGTCATGACCCTCTGGG
2681_F1	TCTGCAATTGTATTTGACAGC
2681_R1	CTGAGTAGAAGCCATCTGTCC
PoliC_F	GTCAAGATTTGCGTCCGAGG
Tgluc_R	CATATGGTAACGCCACATGAGG
cp2672 F1	ATGGAGTCCGTCGTAGACG
cp2672 R1	GATAATAGTACCACTGATTGC
sirC_R1	TAAGTCGTCGAGGTCTAACC
**Expression studies (qRT PCR)**	
RTq_LN3_F	TGTAATGGCATTCGCTTGGGAGC
RTq_LN3_R	ACGGTGTTGGTTCAGAAATTCCTCC
Actin uni	GCCGTTTTCCCCTCTATCGTC
Actin rev	ACATACGAGTCCTTCTGACCCAT
Tub uni	TACAATGGTACCTCGGAGCAAC
Tub rev	CCAGAGGCCTCATTGAAGTAGAC
Gpd uni	CCCGAATATGCTGCCTACATGCT
Gpd rev	CGTCCTTCTTGATCTCGCCCT
**Expression studies (Northern)**	
cp2670 F1	CAGGCAGGTCAATATCTTCG
cp2670 R1	GCTTGAAACATGGTCCACG
OE_Cp_2671_F	CACATCACAATCGATCCAACCATGATGAATCCGATGTTGACCAAC
OE_Cp_2671_R	CATCTTATCTACATACGCTAAGCCTACGGAAGGGTCGCAGTACCC
cp2672 F1	ATGGAGTCCGTCGTAGACG
cp2672 R1	GATAATAGTACCACTGATTGC
cp2673 F1	GGAATTGTACTCCGACAGG
cp2673 R1	GCTTTGTCCACAGTCGTCG
cp2674 F1	TCACCGTCCATCGCCTCG
cp2674 R1	CTCAGTCTTCATTGAGAGG
cp2675 F1	CATCACGAATGACTTCAAGG
cp2675 R1	GTCTCTCTCGTCCAGAACG
cp2676 F1	ATGGAGGCGCAACAAGAC

cp2676 R1	CATGCAGCTCAATCTCTTCC
cp2677 F1	GGAACACGATGGATCAAGG
cp2677 R1	CGATTGATCGAGTGGAAGC
cp2678 F1	TAGAGATGAGTTGGAGCTGG
cp2678 R1	TCAACCCCTTGAATACTGAC
cp2679_F3	CTTGATGGAAGAGCTGCTCC
RTq_LN3_R	ACGGTGTTGGTTCAGAAATTCCTCC
cp2680_F2	CTCATTAACCTCCTAGCACC
RTq_cp2680_R	GAGGGACTGAGTGAAACTCTC
cp2681 R1	CTGAGTAGAAGCCATCTGTCC
cp2681 F	ATGTCTACCAGCAACATTGTCG
cp2682 F	ATGGAGACCGTTTACATCA
cp2682 R	TCAGTCTTCCTTGTGATCT
