# Supplementary material for: The Epipolythiodiketopiperazine Gene Cluster in Claviceps purpurea: Dysfunctional Cytochrome P450 Enzyme Prevents Formation of the Previously Unknown Clapurines
Source: PLoS One. 2016 Jul 8;11(7):e0158945. doi: 10.1371/journal.pone.0158945 (PMC4938161; doi:10.1371/journal.pone.0158945)
Supplement: S2 Table — The structures are depicted in S4 Fig. (PDF) [file pone.0158945.s017.pdf]

δ$_C$ (ppm)										
	**1a**	**1b**	**2a**	**2b**	**3**	**4a**	**5**	**6**	**7**	**11**
**C-2**	165.05	165.14	165.48	165.36	165.45	165.96	165.84	165.94	165.90	163.56
**C-3**	49.80	49.77	49.11	49.18	49.75	45.80	45.65	45.84	45.76	60.01
**C-5**	164.14	164.10	165.30	165.37	165.20	164.40	164.32	164.26	164.39	164.80
**C-6**	65.30	65.26	65.93	65.90	75.90	69.88	69.84	69.96	72.30	72.05
**C-7**	44.42	44.48	44.37	44.36	45.77	45.50	45.56	45.59	45.70	45.56
**C-8**	126.34	126.33	126.79	126.82	126.74	126.89	126.87	126.86	126.84	126.84
**C-9**	132.78	132.71	132.81	132.74	132.95	132.91	133.00	132.98	132.96	132.24
**C-10**	115.65	115.64	116.12	116.08	115.77	115.79	115.74	115.80	115.75	115.79
**C-11**	160.41	160.42	160.15	160.07	159.87	160.01	159.92	160.00	160.04	159.86
**C-12**	115.65	115.66	116.12	116.13	115.77	115.79	115.74	115.780	115.75	115.86
**C-13**	132.78	132.79	132.81	132.82	132.95	132.91	133.00	132.98	132.96	132.29
**C-14**	65.54	65.55	65.80	65.82	65.69	65.83	65.85	65.82	65.80	65.64
**C-15**	120.90	120.91	121.06	121.36	120.98	121.11	121.16	121.09	121.07	121.30
**C-16**	138.54	138.59	138.60	138.78	138.56	138.59	138.61	138.66	138.64	138.62
**C-17**	25.64	25.42	25.74	25.72	25.84	25.84	25.80	25.84	25.83	25.91
**C-18**	18.08	18.25	18.19	18.18	18.19	18.18	18.18	18.19	18.18	18.31
**S-Me**	13.65	13.39	13.75	13.74	13.88	-	-	-	-	-
**O-ME**	-	-	-	-	-	61.99	62.62	61.80	61.59	-
**C-1`**	26.90	26.81	26.91	26.90	-	26.91	32.74	32.80	38.58	-
**C-2`**	32.09	32.22	32.12	32.09	-	32.12	50.68	50.82	51.45	-
**C-3`**	55.32	55.68	55.34	55.48	-	55.34	172.98	173.29	173.25	-
**C-4`**	173.17	173.19	173.27	173.18	-	173.27	-	170.44	-	-
**C-5`**	-		170.34	170.30	-	-	-	22.50	-	-
**C-6`**	-		22.75	22.76	-	-	-	-	-	-
