# Supplementary material for: The Epipolythiodiketopiperazine Gene Cluster in Claviceps purpurea: Dysfunctional Cytochrome P450 Enzyme Prevents Formation of the Previously Unknown Clapurines
Source: PLoS One. 2016 Jul 8;11(7):e0158945. doi: 10.1371/journal.pone.0158945 (PMC4938161; doi:10.1371/journal.pone.0158945)
Supplement: S1 File — (PDF) [file pone.0158945.s018.pdf]

The ^{1}H, ^{13}C, and 2D- NMR spectra were acquired on an Agilent VNMRS 600 (Agilent Technologies, Böblingen, Germany) NMR spectrometer including a cryoprobe. For structural elucidation and NMR signal assignment 2D- NMR experiments, such as (H,H)-correlated spectroscopy (H,H-COSY), Heteronuclear Single Quantum Correlence (gHSQC), heteronuclear multiple bond correlation (HMBC) and Constant time Inverse-detected Gradient Accordion Rescaled long-range Heteronuclear Multiple Bond Correlation (CIGAR-HMBC) were performed. Pulse programs for these experiments were taken from the software library. In case of low amounts and missing signals in the ^{13}C spectrum, we identified the signals in the more sensitive HMBC and HSQC experiment. ^{1}H NMR spectra were recorded at 600 MHz and the ^{13}C NMR experiment at 125 MHz. The ^{1}H NMR data of **1**-**7** and **11** are given below and the ^{13}C NMR data of compounds **1** - **7** as well as **11** are summarized in Table S2. Unfortunately, the NMR Data and subsequent RP-HPLC-HRMS approved a cleavage of the dimethyl allyl group during NMR measurements of compound **6**, resulting in a symmetrical doublet of signals for both compounds.

^{1}H NMR compound **1a** (600 MHz, Methanol-d_4) δ 7.83 (s, 1H), 7.82 (s, 1H), 6.84-6.80 (d, *J* = 2.0 Hz, 2H), 5.42 (tdq, *J* = 7.3, 3.0, 1.4 Hz, 1H), 4.52 – 4.47 (m, 1H), 4.50 – 4.43 (m, 2H), 4.19 (d, *J* = 16.8 Hz, 1H), 3.62 (d, *J* = 6.1 Hz, 1H), 3.49 (d, *J* = 13.5 Hz, 1H), 3.44 (d, *J* = 16.9 Hz, 1H), 2.94 (d, *J* = 13.3 Hz, 1H), 2.65 (qdd, *J* = 13.0, 9.0, 6.2 Hz, 2H), 2.17 – 2.03 (m, 2H), 2.0 (s, 3H), 1.76 (d, *J* = 1.4 Hz, 3H), 1.72 (d, *J* = 1.6 Hz, 3H).

^{1}H NMR compound **1b** (600 MHz, Methanol-d_4) δ 7.13 (s, 1H), 7.11 (s, 1H), 6.85 -6.80 (d, 2H), 5.42 (tdq, *J* = 7.3, 3.0, 1.4 Hz, 1H), 4.51 – 4.50 (m, 1H), 4.50 – 4.45 (m, 2H), 4.19 (d, *J* = 16.8 Hz, 1H), 3.62 (d, *J* = 6.1 Hz, 1H), 3.49 (d, *J* = 13.5 Hz, 1H), 3.44 (d, *J* = 16.9 Hz, 1H), 2.96 (d, *J* = 13.5 Hz, 1H), 2.88 (qdd, *J* = 13.0, 9.6, 6.2 Hz, 2H), 2.17 – 2.03 (m, 2H), 2.0 (s, 3H), 1.77 (d, *J* = 1.4 Hz, 3H), 1.73 (d, *J* = 1.3 Hz, 3H).

^{1}H NMR compound **2a** (600 MHz, Methanol-d_4) δ 7.20 (s, 1H), 7.15 (s, 1H), 6.78 (s, 1H), 6.76 (s, 1H), 5.44 (tdq, *J* = 7.0, 2.8, 1.2 Hz, 1H), 4.51 – 4.50 (m, 1H), 4.53 – 4.44 (m, 2H), 4.21 (d, *J* = 16.7 Hz, 1H), 3.65 (d, *J* = 6.1 Hz, 1H), 3.50 (d, *J* = 13.0 Hz, 1H), 3.42 (d, *J* = 16.9 Hz, 1H), 2.96 (d, *J* = 13.3 Hz, 1H), 2.88 (qdd, *J* = 13.0, 9.6, 6.2 Hz, 2H), 2.17 – 2.03 (m, 2H), 1.84 (s,3H), 2.01 (s, 3H), 1.68 (d, *J* = 1.5 Hz, 3H), 1.64 (d, *J* = 1.2 Hz, 3H).

^{1}H NMR compound **2b** (600 MHz, DMSO-d_6) δ 7.20 (s, 1H), 7.15 (s, 1H), 6.80 (d, *J* = 2.1 Hz, 1H), 6.76 (d, *J* = 2.0 Hz, 1H), 5.44 (tdq, *J* = 7.0, 2.8, 1.2 Hz, 1H), 4.51 – 4.50 (m, 1H), 4.53 – 4.44 (m, 2H), 4.21 (d, *J* = 16.7 Hz, 1H), 3.65 (d, *J* = 6.1 Hz, 1H), 3.50 (d, *J* = 13.0 Hz, 1H), 3.44 (d, *J* = 16.9 Hz, 1H), 2.96 (d, *J* = 13.5 Hz, 1H), 2.88 (qdd, *J* = 13.0, 9.6, 6.2 Hz, 2H), 2.17 – 2.03 (m, 2H), 1.84 (s,3H), 2.01 (s, 3H), 1.68 (d, *J* = 1.5 Hz, 3H), 1.64 (d, *J* = 1.3 Hz, 3H).

^{1}H NMR compound **3** (600 MHz, DMSO-d_6) δ 7.05 (d, *J* = 7.2 Hz, 2H), 6.91 – 6.86 (m, 2H), 5.43 (dt, *J* = 7.3, 3.7 Hz, 1H), 5.05 – 4.99 (m, 1H), 4.31 – 4.24 (m, 1H), 4.09 (d, *J* = 3.9 Hz, 2H), 3.16 (d, *J* = 12.4 Hz, 1H), 2.96 (d, *J* = 12.4 Hz, 1H), 2.00 (s, 3H), 1.72 (d, *J* = 1.5 Hz, 3H), 1.70 (d, *J* = 1.4 Hz, 3H).

^{1}H NMR compound **4a** (600 MHz, Methanol-d_4) δ 7.13 (d, *J* = 2.1 Hz, 1H), 7.11 (s, 1H), 6.84 (d, *J* = 2.1 Hz, 1H), 6.83 (d, *J* = 2.0 Hz, 1H), 5.42 (tdq, *J* = 7.0, 2.9, 1.4 Hz, 1H), 4.51 – 4.46 (m, 1H), 4.50 – 4.49 (m, 2H), 4.17 (d, *J* = 16.6 Hz, 1H), 3.65 (s, 3H), 3.62 (d, *J* = 6.1 Hz, 1H), 3.49 (d, *J* = 13.5 Hz, 1H), 3.44 (d, *J* = 16.9 Hz, 1H), 2.96 (d, *J* = 13.5 Hz, 1H), 2.88 (qdd, *J* = 12.4, 9.6, 6.2 Hz, 2H), 2.17 – 2.03 (m, 2H), 1.77 (d, *J* = 1.4 Hz, 3H), 1.73 (d, *J* = 1.3 Hz, 3H).

^{1}H NMR compound **5** (600 MHz, Methanol-d_4) δ 7.13 (d, *J* = 2.0 Hz, 1H), 7.12 (s, *J* = 2.1 Hz 1H), 6.82 (d, *J* = 2.0 Hz, 1H), 6.80 (d, *J* = 2.0 Hz, 1H), 5.42 (td, *J* = 6.2, 3.1 Hz, 2H)4.50 – 4.49 (m, 1H), 4.10 – 4.49 (m, 2H), 4.14 (d, *J* = 17.4 Hz, 1H), 3.64 (s, 3H), 3.61 (d, *J* = 6.1 Hz, 1H), 3.49 (d, *J* = 13.4 Hz, 1H), 3.44 (d, *J* = 16.4 Hz, 1H), 3.07 – 3.18 (m, 2H), 2.96 (d, *J* = 13.2 Hz, 1H), 1.73 (d, *J* = 1.3 Hz, 3H), 1.70 (d, *J* = 1.3 Hz, 3H).

^{1}H NMR compound **6** (600 MHz, Methanol-d_4) δ 7.13 (d, *J* = 2.0 Hz, 1H), 7.12 (s, *J* = 2.1 Hz 1H), 6.84 (d, *J* = 2.0 Hz, 1H), 6.82 (d, *J* = 2.0 Hz, 1H), 5.42 (td, *J* = 6.1, 3.0 Hz, 2H)4.50 – 4.48 (m, 1H), 4.50 – 4.49 (m, 2H), 4.16 (d, *J* = 17.2 Hz, 1H), 3.58 (s, 3H), 3.80 (t, *J* = 6.9 Hz, 1H), 3.51 (d, *J* = 13.4 Hz, 1H), 3.44 (d, *J* = 16.4 Hz, 1H), 3.07 – 3.18 (m, 2H), 2.96 (d, *J* = 13.2 Hz, 1H), 2.32 (s,3H), 1.75 (d, *J* = 1.4 Hz, 3H), 1.72 (d, *J* = 1.2 Hz, 3H).

^{1}H NMR compound **7** (600 MHz, Methanol-d_4) δ 7.09 – 7.05 (m, 2H), 6.85 – 6.82 (m, 2H), 5.43 (m, 1H), 5.15 – 5.10 (m, 1H), 4.66 – 4.59 (m, 1H), 4.09 (d, *J* = 3.6 Hz, 2H), 3.97 (t, *J* = 7.0 Hz, 1H), 3.58 (s, 3H), 3.48 – 3.42 (m, 1H), 3.17 (dd, *J* = 12.1, 7.0 Hz,

1H), 2.99 (d, *J* = 12.5 Hz, 1H), 2.92 (dd, *J* = 12.4 Hz, 1H), 1.73 (d, *J* = 1.5 Hz, 3H), 1.70 (d, *J* = 1.3 Hz).

^{1}H NMR compound **11** (600 MHz, Methylene Chloride-d_2) δ 7.22 – 7.17 (d, *J* = 8.54 Hz, 2H), 6.87 – 6.85 (d, *J* = 8.59 Hz, 2H), 6.10 (s, 1H), 5.95 (s, 1H), 5.39 (m, 1H), 4.52 – 4.50 (m, 2H), 5.20 (s, 1H), 3.50 (d, *J* = 14.73 Hz, 1H), 3.25 (d, *J* = 14.71 Hz, 1H), 1.79 (d, *J* = 1.4 Hz, 3H), 1.74 (d, *J* = 1.24 Hz).
